# Autism NPCs from both idiopathic and CNV 16p11.2 deletion patients exhibit dysregulation of proliferation and mitogenic responses

**DOI:** 10.1016/j.stemcr.2022.04.019

**Published:** 2022-05-26

**Authors:** Robert Connacher, Madeline Williams, Smrithi Prem, Percy L. Yeung, Paul Matteson, Monal Mehta, Anna Markov, Cynthia Peng, Xiaofeng Zhou, Courtney R. McDermott, Zhiping P. Pang, Judy Flax, Linda Brzustowicz, Che-Wei Lu, James H. Millonig, Emanuel DiCicco-Bloom

**Affiliations:** 1 Department of Neuroscience and Cell Biology, Rutgers Robert Wood Johnson Medical School, Piscataway, NJ, USA; 2 Graduate Program in Neuroscience, Rutgers University, Piscataway, NJ, USA; 3 Child Health Institute of New Jersey, Rutgers University, New Brunswick, NJ, USA; 4 Center for Advanced Biotechnology and Medicine, Rutgers University, Piscataway, NJ, USA; 5 Department of Molecular Biology and Biochemistry, Rutgers University, Piscataway, NJ, USA; 6 Department of Cell Biology and Neuroscience, Rutgers University, Piscataway, NJ, USA; 7 Department of Genetics, Rutgers University, Piscataway, NJ, USA; 8 Department of Obstetrics, Gynecology, and Reproductive Sciences, Rutgers Robert Wood Johnson Medical School, New Brunswick, NJ, USA; 9 Department of Pediatrics, Rutgers Robert Wood Johnson Medical School, New Brunswick, NJ, USA

**Keywords:** autism spectrum disorders, human iPSCs, human NPCs, proliferation, copy number variant, 16p11.2 deletion, phospho-ERK signaling, basic FGF, macrocephaly

## Abstract

Neural precursor cell (NPC) dysfunction has been consistently implicated in autism. Induced pluripotent stem cell (iPSC)-derived NPCs from two autism groups (three idiopathic [I-ASD] and two 16p11.2 deletion [16pDel]) were used to investigate if proliferation is commonly disrupted. All five individuals display defects, with all three macrocephalic individuals (two 16pDel, one I-ASD) exhibiting hyperproliferation and the other two I-ASD subjects displaying hypoproliferation. NPCs were challenged with bFGF, and all hyperproliferative NPCs displayed blunted responses, while responses were increased in hypoproliferative cells. mRNA expression studies suggest that different pathways can result in similar proliferation phenotypes. Since 16pDel deletes *MAPK3*, P-ERK was measured. P-ERK is decreased in hyperproliferative but increased in hypoproliferative NPCs. While these P-ERK changes are not responsible for the phenotypes, P-ERK and bFGF response are inversely correlated with the defects. Finally, we analyzed iPSCs and discovered that 16pDel displays hyperproliferation, while idiopathic iPSCs were normal. These data suggest that NPC proliferation defects are common in ASD.

## Highlights

• NPC proliferation defects are common in idiopathic and 16p11.2 CNV-deletion ASD
• All macrocephalic I-ASD and 16pDel individuals have hyperproliferative NPCs
• NPC proliferative responses to bFGF correlate inversely with P-ERK levels
• Both NPCs and IPSCs derived from 16pDel individuals exhibit hyperproliferation

## Teaser

In this article DiCicco-Bloom and colleagues show proliferation defects in neural precursor cells from idiopathic and 16p11.2-deletion forms of autism. Those exhibiting macrocephaly were hyperproliferative, while two idiopathic individuals displayed hypoproliferative phenotypes. Interestingly, 16pDel individuals also exhibited iPSC-hyperproliferative defects. These findings suggest that dysregulated proliferation may be a common phenotype in multiple forms of autism and span developmental stages.

## Introduction

Autism spectrum disorder (ASD) is a heterogeneous neurodevelopmental disorder characterized by difficulties with social interactions and communication and the presence of repetitive and restricted behaviors. Most ASD cases are idiopathic, having no known genetic cause, and only 15%–20% of ASD cases are caused by known mutations such as copy number variants (CNVs) or monogenic mutations (De La Torre-Ubieta et al., 2016). Although ASD neuropathology studies have not uncovered consistent defects, overall dysregulation of early brain development, including abnormal proliferation, has been implicated. Altered proliferation is consistent with reported changes in cortical and cerebellar neuronal numbers, macrocephaly, an imbalance in excitatory-inhibitory neurons, and focal cortical dysplasias (Amaral et al., 2008; Chomiak et al., 2013; Varghese et al., 2017). Further, several studies indicate that genetically identified autism risk genes are expressed in the midfetal cerebral cortex and specifically in radial glial cells, which are cortical neural precursor cells (NPCs; Willsey et al., 2013; Grove et al., 2019; Satterstrom et al., 2020). While cortical NPC proliferation abnormalities may contribute to ASD etiology, few studies have investigated this directly. Importantly, transcriptome studies indicate that induced pluripotent stem cell (iPSC)-derived NPCs resemble fetal forebrain, making them a relevant system to investigate this important question (Brennand et al., 2014, 2015).

Macrocephaly is observed in about 20% of idiopathic ASD cases and with certain CNVs, including 16p11.2 deletion. Structural imaging studies indicate that macrocephaly frequently results from increased brain mass, especially involving frontal and parietal lobes and the cerebellum. Individuals with 16p11.2 CNV deletion display macrocephaly ∼17% of the time (Steinman et al., 2016). The CNV either deletes or duplicates 28 genes, including *MAPK3*, which encodes ERK1. Both the deletion and the duplication are correlated with increased autism risk (Devlin and Scherer, 2012; Niarchou et al., 2019) and display mirror growth phenotypes. The deletion (16pDel) often leads to macrocephaly and macrosomia, while the duplication (16pDup) exhibits microcephaly and small stature (Shinawi et al., 2010; Qureshi et al., 2014). This correlation of CNV dosage to brain size suggests not only that the genes directly influence neurogenesis, but also that “too few” or “too many” neurons lead to atypical development. Previous mouse 16pDel studies do not demonstrate macrocephaly, but cortical NPCs do display hyperproliferation. This is correlated with phosphorylated ERK1 (P-ERK) signaling due to the deletion of *Mapk3* (Pucilowska et al., 2015, 2018). While iPSC models of idiopathic, macrocephalic ASD individuals have been reported, and they exhibit increased NPC proliferation (Mariani et al., 2015, Marchetto et al., 2017), these studies are not extensive and have not been extended to CNVs like 16pDel. Thus, studies utilizing human iPSC-derived NPCs from both idiopathic and genetically defined datasets like 16pDel would be ideal for studying possible proliferative phenotypes that may contribute to ASD.

In this study we investigated the proliferation of iPSC-derived NPCs from three idiopathic and two 16pDel ASD subjects and controls. Our findings indicate that proliferation is dysregulated in all five ASD individuals. We find hyperproliferation in macrocephalic individuals from both the 16pDel and the idiopathic (I-ASD) subgroups, while the other individuals exhibit hypoproliferation. Interestingly, the proliferation phenotype is inversely correlated with P-ERK levels and response to basic fibroblast growth factor (bFGF), a mitogen that stimulates the ERK pathway. In examining whether proliferation dysregulation is specific to the cells of the brain, we find that the I-ASD cohort shows no differences in their iPSCs. In contrast, iPSC proliferation in 16pDel ASD subjects parallels the increases seen in NPC proliferation. In aggregate, our findings suggest that idiopathic and 16pDel subgroups share a common phenotype of dysregulated NPC proliferation that for our datasets is inversely correlated with P-ERK levels and bFGF stimulation.

## Results

### Study design for robust and reproducible measures of proliferation

**Figure 1:**
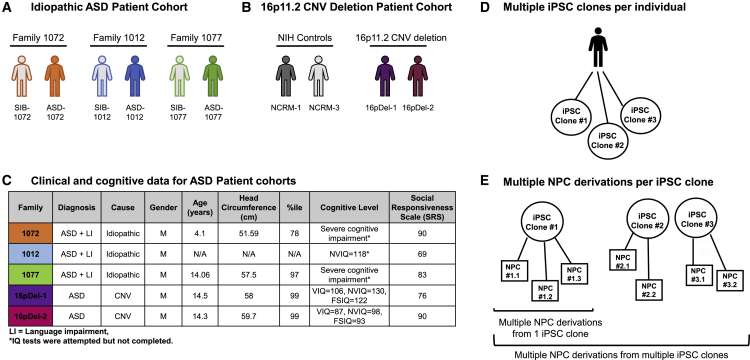
Patient datasets and study design (A) For the idiopathic autism (I-ASD) dataset, iPSCs were generated from three families (family 1072, orange; 1012, blue; and 1077, green). All families had one male child diagnosed with autism and a clinically determined unaffected brother (SIB). (B) For the 16pDel ASD dataset, iPSCs were obtained for two male individuals with autism bearing 16p11.2 deletion (purple and maroon). iPSCs from male unaffected individuals were obtained from the NIH (light gray and dark gray). (C) Clinical data for I-ASD and 16pDel individuals are shown, including patient diagnosis, age at cell collection, Social Response Scale score (SRS), cognitive level, patient head circumference (HC), and HC percentile. (D) The studies utilized between two and five randomly selected iPSC clones per individual. (E) Hypothetical example demonstrating how each iPSC clone was used to derive multiple NPC lines. LI, language impairment; N/A, not available. ^∗^See supplementary information for cognitive assessments. See Figure S1 for iPSC characterization and Figures S2 and S3 for NPC marker expression and quantification, respectively.

To generate an I-ASD dataset, male autism probands and sex-matched, unaffected siblings from three families were chosen from the larger New Jersey Language and Autism Genetics Study (NJLAGS) (Figure 1A) (Bartlett et al., 2012, 2014). NJLAGS families were recruited for family members with ASD, plus a separate family member with a language disorder called language-based learning impairment (LLI) that affects only language development. These families thus have one proband with ASD and another proband with LLI. This strategy of recruiting for two language disorders reduces phenotypic and potentially genetic heterogeneity. Importantly, all family members were phenotyped extensively and were evaluated for ASD and LLI by the same set of clinicians (see supplemental information). Thus, the unaffected sibling control for each family is diagnostically determined to not have ASD or LLI. Among the families, ASD-1072 has a normal head circumference, whereas ASD-1077 has a head circumference in the 97th percentile that is consistent with macrocephaly. No data were available for the head circumference of ASD-1012 (Figure 1C).

For the 16pDel cohort, we selected from RUCDR Infinite Biologics the only two deletion males diagnosed with autism by the Simons VIP cohort for whom iPSCs were created (see supplemental information). Both males (16pDel-1, 16pDel-2) had a head circumference at the 99th percentile (Figure 1C). For sex-matched controls, we selected two unrelated iPSC lines from the NIH Regenerative Medicine Program (NCRM-1, NCRM-3) (Figure 1B).

For each subject, multiple iPSC clones were generated. To ensure pluripotency and quality control, iPSC lines were karyotyped and assessed for expression of molecular makers of pluripotency (Figure S1). A minimum of two and up to five iPSC clones were used to induce into NPCs for each individual (Figure 1D), with the exception of NIH controls, for which only one iPSC clone was available. In addition, multiple NPC inductions were conducted for each iPSC clone (Figure 1E), especially when iPSC clone numbers were limited. To ensure quality control of NPCs, all lines were routinely immunostained for NPC markers (Nestin, SOX2, Pax6) prior to use for any experiments (see supplemental experimental procedures and Figures S2 and S3). NPC derivations were excluded from use if Nestin and/or Sox2 cell expression was <85% or Pax6 was <60%. Exact numbers of iPSC clones used to derive NPCs as well as numbers of NPC inductions per individual for all experiments are reported in Table S1.

### All I-ASD individuals display dysregulated proliferation

To rigorously define proliferation in human NPCs, we employed a multi-tiered strategy consisting of simple and reproducible assays followed by more in-depth analyses to further characterize neurogenesis. We examined multiple measures of cell proliferation and cell death: total DNA synthesis by [^3^H]thymidine labeling, S-phase labeling index, cell enumeration assays, and apoptotic marker cleaved caspase-3 immunocytochemisty (ICC). Using this strategy, we found that all I-ASD subjects display NPC proliferative differences compared with unaffected sibling controls. I-ASD individuals from two different families (families 1072 and 1012) exhibited reductions in NPC proliferation, whereas in the third macrocephalic ASD subject (family 1077), NPCs exhibited an increase in proliferation.

**Figure 2:**
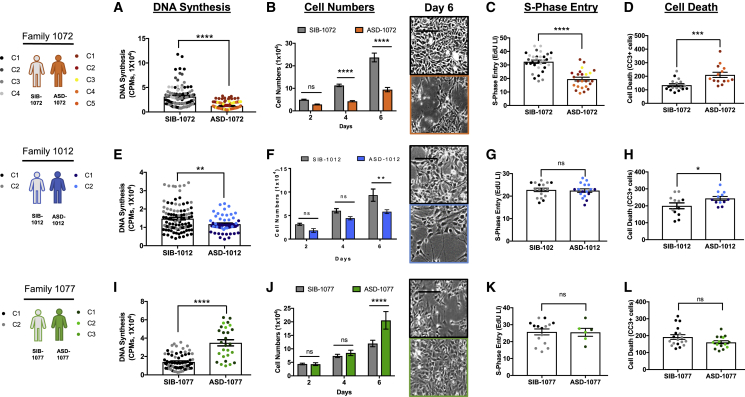
Idiopathic ASD NPCs from three families display dysregulated proliferation phenotypes (A–D) At 48 h, (A) Family 1072 ASD exhibited a 65% reduction in DNA synthesis in comparison to SIB and (B) significantly reduced cell numbers at 4 and 6 days. Representative images of day 6 cultures are shown. Scale bars: 50 μm. (C) Family 1072 ASD also displayed a 40% reduction in S-phase entry (labeling index) at 48 h (see Figure S4 for images), and (D) at 24 h, a 55% increase in number of cells expressing cleaved caspase-3 (CC3). Different colored data points represent different iPSC clones used for NPC derivations. (E–H) Family 1012 ASD exhibited (E) a 20% reduction in 48 h DNA synthesis and (F) at 6 days displayed a 40% reduction in cell numbers shown in representative images. (G) Family 1012 ASD exhibited no difference in S-phase entry at 48 h, while (H) at 24 h displayed a 22% increase in cells expressing CC3. (I–L) Family 1077 ASD, who has macrocephaly, exhibited (I) a 60% increase in DNA synthesis and (J) a 30% increase in cell numbers at day 6, shown in representative images. (K) Family 1077 ASD displayed no change in S-phase entry at 48 h, and (L) at 24 h, no difference in numbers of cells expressing CC3. For the three sib pairs, the sample sizes were number of individuals, number of iPSC clones, number of NPC derivations, number of experiments, and number of wells, and the ranges for each sib pair were, for Family 1072 (A–D), SIB, 1, 2–4, 2–5, 6–31, and 17–105, and ASD, 1, 2–5, 3–9, 5–36, and 14–118; for Family 1012 (E–H), SIB, 1, 1–2, 1–5, 2–21, and 5–106, and ASD, 1, 1–2, 1–8, 2–14, and 6–56; and for Family 1077 (I–L), SIB, 1, 2, 2–5, 5–25, and 15–74, and ASD, 1, 2–3, 2–4, 2–9, and 6–27. See Table S1 for iPSC and NPC sample size details. Data represent the mean ± SEM (^∗^p < 0.05, ^∗∗^p < 0.01, ^∗∗∗^p < 0.001, ^∗∗∗∗^p < 0.0001).

In the first I-ASD family examined, family 1072, we conducted numerous blinded experiments (31 for the sibling control and 36 for the ASD individual), comparing multiple separate derivations of NPCs derived from four sibling iPSC clones with those from five ASD iPSC clones. The ASD NPCs exhibited a significant 65% reduction in DNA synthesis at 48 h by ^3^H labeling (Figure 2A). To see if changes in DNA synthesis had an impact on cell production, cell numbers were enumerated and family 1072 ASD NPCs displayed 62% and 65% reductions at 4 and 6 days, respectively (Figure 2B). In examining mechanisms underlying reduced cell proliferation, decreases in total DNA synthesis were paralleled by a 40% reduction in cells entering S phase at 48 h (Figure 2C), and ASD NPCs displayed a 55% increase in number of cells expressing cleaved caspase-3 (CC3)^+^ at 24 h (Figure 2D). These data suggest that both a smaller proliferative population and an increase in cell death contribute to the NPC proliferation deficits in family 1072.

Similar to family 1072, ASD-1012 NPCs also exhibited a decrease in DNA synthesis, by 20% at 48 h (Figure 2E), and a 40% decrease in cell numbers at 6 days (Figure 2F). However, there was no difference in the proportion of cells entering S phase (Figure 2G), but ASD-1012 NPCs displayed a 22% increase in cell death (Figure 2H), suggesting potentially different mechanisms for the reduced cell numbers in families 1012 and 1072.

In marked contrast to these two hypoproliferative families, macrocephalic ASD-1077 NPCs displayed a 60% increase in DNA synthesis (Figure 2I) that was paralleled by a 30% increase in cell numbers at 6 days (Figure 2J). There were no differences in the proportion of cells entering S phase at 48 h (Figure 2K) nor in the proportion of cells dying at 24 h (p = 0.0771; Figure 2L). Since differences in cell numbers appeared only at 6 days despite increases in DNA synthesis at 48 h, these observations suggest that changes in longer-term cell survival or differentiation may play a role, which could be explored in future analyses.

In sum, all I-ASD NPCs display a proliferation phenotype (two hypoproliferative and one hyperproliferative) compared with their respective sibling. Notably, all three I-ASD individuals are not related to one another and remain genetically undefined, yet they converge on proliferative defects.

### Two macrocephalic autism 16pDel individuals exhibit NPC hyperproliferation

**Figure 3:**
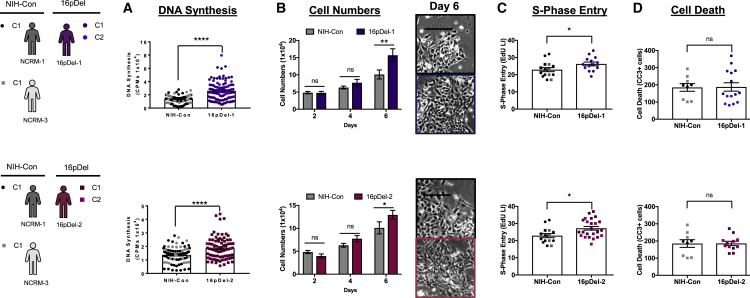
NPCs from two individuals with 16p11.2 CNV deletion (16pDel), autism, and macrocephaly exhibit hyperproliferation in comparison with NIH controls (A–D) 16pDel-1 NPCs (A) exhibited a 90% increase in DNA synthesis, whereas 16pDel-2 exhibited a 36% increase in DNA synthesis compared with NIH controls at 48 h. (B) After 6 days, 16pDel-1 and 16pDel-2 exhibited 55% and 28% increases, respectively, in cell numbers. Representative images are shown. Scale bars: 50 μm. (C) 16pDel-1 and 16pDel-2 exhibited 15% and 19% increases, respectively, in 48 h S-phase entry. (D) 16pDel-1 and 16pDel-2 exhibited no differences in cells expressing CC3 at 24 h. Data represent the mean ± SEM (^∗^p < 0.05, ^∗∗^p < 0.01, ^∗∗∗∗^p < 0.0001). See Figure S4 for Edu labeling and Figure S5 for NIH control and 16pDel comparisons. The sample sizes were number of individuals, number of iPSC clones, number of NPC derivations, number of experiments, and number of wells, and the ranges for each individual were 16pDel-1, 1, 2, 2–5, 4–31, and 10–108; NCRM, 2, 2, 2–9, 3–21, and 9–75. See Table S1 for iPSC and NPC sample size details.

To inquire if NPC proliferation is also altered in the 16pDel cohort, we compared NPCs from the 16pDel individuals with two sex-matched genetically typical controls. Both 16pDel individuals are macrocephalic and exhibit a significant increase in DNA synthesis at 48 h (Figure 3A). Specifically, a 90% increase was observed for 16pDel-1 and 36% increase for 16pDel-2. This 90% increase for 16pDel-1 was significantly different from that of 16pDel-2, suggesting that variability in phenotype can exist among individuals with the same CNV (Figure S5).

This increase in early DNA synthesis was paralleled by a 55% increase in cell numbers after 6 days for 16pDel-1, and 28% for 16pDel-2 (Figure 3B). Single-cell analysis indicated that both 16pDel-1 and 16pDel-2 NPCs exhibited increased S-phase entry at 48 h, with 15% and 19% increases, respectively, compared with NIH NPCs (Figure 3C). On the other hand, neither of the 16pDels’ NPCs exhibited differences in CC3^+^ cells compared with NIH controls at 24 h (Figure 3D). Our finding of increased NPC proliferation that correlates with macrocephaly supports previous reports (Mariani et al., 2015; Marchetto et al., 2017). These data also show for the first time that human NPCs from 16pDel individuals with macrocephaly display increased proliferation, evidenced by increases in DNA synthesis, cell numbers, and EdU labeling index.

### Dysregulation of proliferation in autism NPCs correlates inversely with mitogenic response to basic fibroblast growth factor

**Figure 4:**
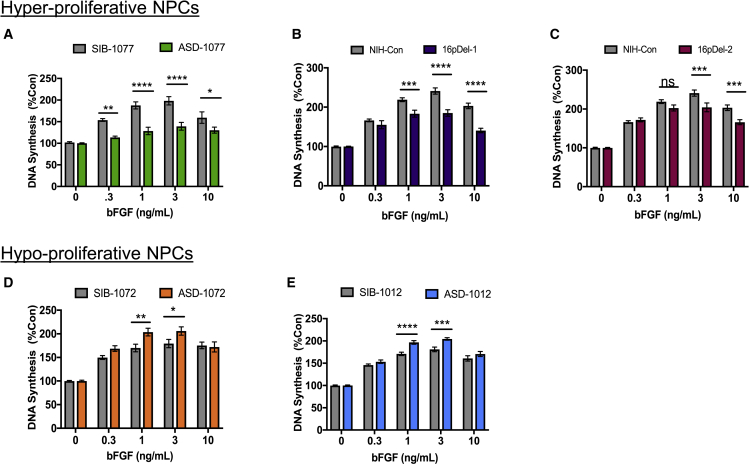
ASD NPCs exhibit altered mitogenic sensitivity and response to bFGF that correlates inversely with proliferation phenotypes DNA synthesis was assessed at 48 h of culture incubation. (A–C) Hyperproliferative NPCs exhibit reduced sensitivity and response to bFGF mitogenic stimulation. (A) Family 1077 ASD displayed ∼20%–30% reduction in responses to bFGF over the 0.3 to 3 ng/mL dose range. (B) 16pDel-1 displayed 16%–30% reduction in responses to bFGF over 1 to 10 ng/mL. (C) 16pDel-2 displayed 15%–18% reduction in responses to bFGF over 3 to 10 ng/mL. (D and E) Hypoproliferative NPCs exhibit enhanced sensitivity and response to bFGF mitogenic stimulation. (D) Family 1072 ASD displayed ∼20% increase in responses to bFGF over 1 to 3 ng/mL. (E) Family 1012 ASD displayed 15% increase in responses to bFGF over 1 to 3 ng/mL bFGF. The sample sizes were number of individuals, number of iPSC clones, number of NPC derivations, number of experiments, and number of wells, and the ranges for each individual were control, 1, 1–2, 2–3, 3–6, and 9–18; and ASD, 1, 2–3, 2–4, 5–6, and 14–21. See Table S1 for iPSC and NPC sample size details. Differences in bFGF responses did not reflect differential cell-cell contact under control or growth factor conditions; see Figure S6. Data represent the mean ± SEM (^∗^p < 0.05, ^∗∗^p < 0.01, ^∗∗∗^p < 0.001, ^∗∗∗∗^p < 0.0001).

Finding that both 16pDel and I-ASD cohorts exhibited altered proliferation, we tested if these phenotypes might be correlated with changes in response to mitogenic signaling, which affects both cell proliferation and death. We challenged NPCs with a developmentally relevant mitogen, bFGF (FGF2), that is required for normal control of cortical progenitor proliferation (Wagner et al., 1999; Li and DiCicco-Bloom, 2004; Thisse and Thisse, 2005; Stevens et al., 2010). Following stimulation of NPCs for 48 h with a range of bFGF concentrations (0.03–10 ng/mL), we measured total DNA synthesis. NPC response to bFGF is expressed as a percentage of control (Figure 4). While all individuals responded to bFGF stimulation and displayed a dose-response profile, differences in response magnitude were observed (Figure 4). Notably, NPCs that shared a hyperproliferative phenotype across both datasets (ASD-1077, 16pDel-1, 16pDel-2; Figures 2 and 3) exhibited a less robust response to bFGF stimulation. Specifically, the ASD-1077 NPC response to bFGF was blunted by ∼20%–30% compared with the sib control (Figure 4A). 16pDel-1 displayed an ∼15%–30% reduction in response and 16pDel-2 displayed ∼15%–20% diminished response to bFGF compared with NIH controls (Figures 4B and 4C).

In contrast, ASD NPCs that exhibited hypoproliferation (I-ASD-1072 and -1012) displayed an increase in DNA synthesis sensitivity and response to bFGF stimulation (Figures 4D and 4E). Both ASD-1072 and ASD-1012 displayed a 15%–20% larger response to bFGF in comparison with sibs. Together, these data indicate that NPC proliferation phenotypes are observed across our two ASD cohorts, and within our datasets, bFGF stimulation is inversely correlated with the proliferative defect. Differences in proliferation and mitogenic response may reflect alterations in the components of the FGF signaling pathway, including FGF receptors, receptor adaptor proteins, and activation of downstream kinases.

### Molecular analysis of I-ASD and 16pDel NPCs

#### mRNA expression analysis

**Figure 5:**
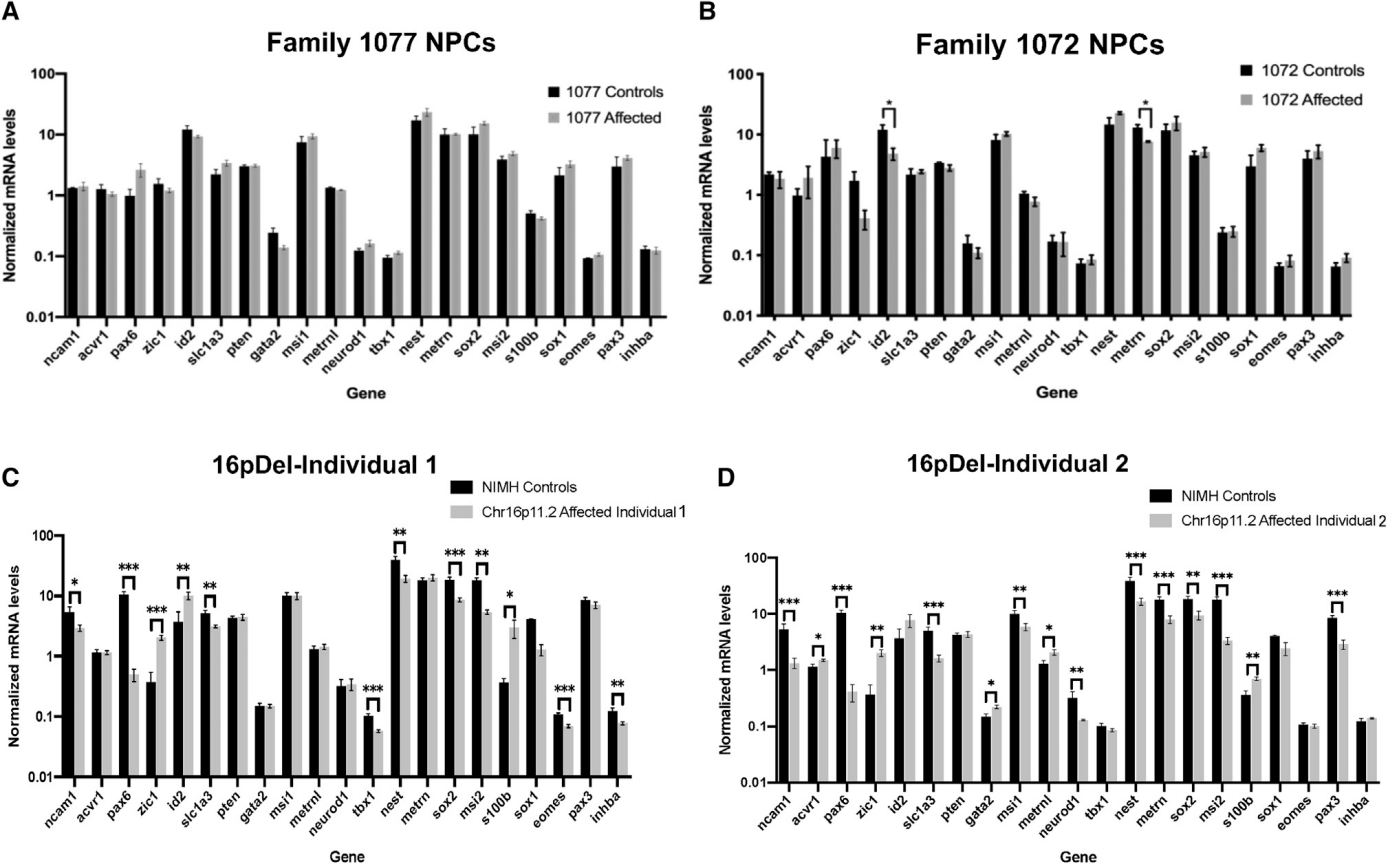
Gene expression of 24 NPC markers in I-ASD families and 16pDel NPCs (A–D) NPC mRNA levels were measured using a multiplex Quantiplex panel. (A) For Family 1077, no significant differences were observed compared with the sib control, while (B) for Family 1072, two minimally significant differences were observed. For both families, three iPSC clones for both sib and I-ASD were analyzed. For 16pDel NPCs, multiple significant changes in mRNA levels were observed compared with NIH controls. (C) For 16pDel-1, two iPSC clones were analyzed and two different NIH individuals were used as controls. (D) For 16pDel-2, three iPSC clones were analyzed and two different NIH individuals were used as controls (^∗^p < 0.05, ^∗∗^p < 0.01, ^∗∗∗^p < 0.001; Student t test). See Table S1 for iPSC and NPC details.

The above analysis suggests that there are overlapping proliferation phenotypes for 16pDel and I-ASD but also individual-specific differences, implying that underlying mechanisms may be different. To examine this further, we next measured the mRNA expression levels for a series of proliferative NPC markers. We investigated if each ASD individual had his own individualized gene expression pattern or, alternatively, if there were similarities among all ASD cases or within the subgroups (I-ASD and 16pDel). A multiplex, high-throughput Quantiplex panel was developed for 21 genes that are used as markers for human NPC proliferation. NPCs from the two I-ASD families with the greatest differences in proliferation (families 1072 and 1077) were examined. Family 1077 revealed no significant changes (Figure 5A), while family 1072 had only two minimally significantly differences (*ID2* and *METRN*) compared with sibling controls (Figure 5B). In contrast, the 16pDel NPCs displayed remarkable gene expression changes. Both 16pDel-1 and 16pDel-2 individuals had 12 and 15 significant differences, respectively, with eight mRNAs significantly changed in both 16pDel individuals (Figures 5C and 5D). Interestingly, 16pDel NPCs display significant mRNA reductions in both *NCAM* and *PAX6* as well as increases in *S100*. This may suggest an increased probability of glial fate. However, at 48 h, our cells immunostained only with NPC markers (Figures S2 and S3) and neither cells nor protein extracts expressed a glial marker, GFAP or S100, by ICC or western (data not shown). Thus, the marker analysis demonstrates similarities within groups but also individual differences, supporting the idea that the mechanisms underlying the common proliferation defects may vary between subgroups and individuals.

#### P-MAPK/ERK levels

**Figure 6:**
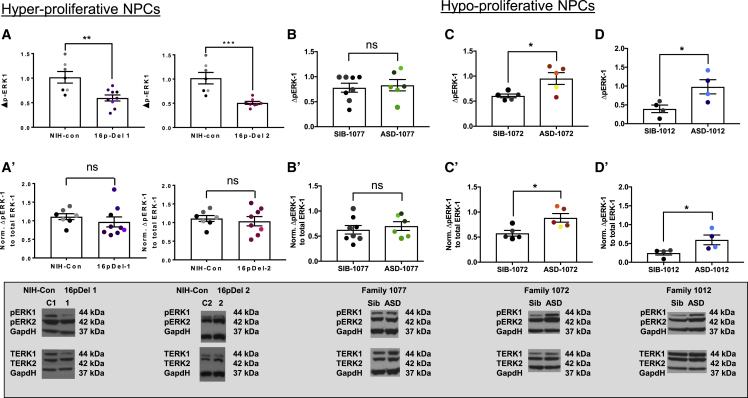
Absolute levels of intracellular P-ERK1 signaling inversely correlate with ASD NPC proliferation (A and A′) Hyperproliferative 16pDel-1 and 16pDel-2 displayed ∼50% reduction in P-ERK1/Gapdh levels. Normalized P-ERK1 levels for 16pDel-1 and 16pDel-2 exhibited no significant change. (B and B′) Family 1077 exhibited unchanged P-ERK1/Gapdh and normalized P-ERK1 levels. (C, C′, D, and D′) (C and C′) Hypoproliferative family 1072 and (D and D′) family 1012 exhibited increased P-ERK1/Gapdh and normalized P-ERK1 levels. Representative western blot images of phosphorylated ERK1/2, total ERK, and Gapdh loading control are provided for each control and ASD individual. Different colored data points represent different iPSC clones, as shown in Figures 2 and 3, and described in Figure 2 legend. Note: western blot films were cropped to align loading control and ERK bands. The sample sizes were number of individuals, number of iPSC clones, number of NPC derivations, number of experiments, and number of wells, and the ranges for each individual were control, 1–2, 2–3, 3–5, 2–4, and 4–8; and ASD, 1, 2–4, 2–4, 2–4, and 4–6. See Table S1 for iPSC and NPC sample size details. Data represent the mean ± SEM (^∗^p < 0.05, ^∗∗^p < 0.01, ^∗∗∗^p < 0.001).

Next, we investigated ERK levels and whether they correlated with the proliferation phenotypes. The 16p deletion includes *MAPK3*, which encodes ERK1. Given that ERK signaling affects cell growth and proliferation, we measured levels of phosphorylated and total ERK1 protein. We first examined NPCs with a hyperproliferative phenotype. As expected, NPCs from both 16pDel individuals exhibited a 50% reduction in total ERK1 protein and P-ERK1 (Figures 6A and 6A′). There was no difference in ERK2. Not surprisingly, when the ratio of P-ERK1 to total ERK1 was examined, it was not different between control and 16pDel NPCs. In family 1077 I-ASD, however, there were no differences in the levels of P-ERK1 (Figures 6B and 6B′), suggesting that other signaling mechanisms may contribute to this phenotype in this family. Nevertheless, for the two 16pDel individuals examined in this study, decreased ERK and P-ERK1 levels were correlated with 16pDel NPC hyperproliferation.

Given the reductions in P-ERK1 in hyperproliferative 16pDel NPCs, we wondered if there may be similar correlations in the hypoproliferative I-ASD families (ASD-1072 and -1012). Interestingly, the opposite signaling pattern was observed: a 50% increase in the levels of both total and normalized P-ERK1 (Figures 6C, 6C′, 6D, and 6D′). We further investigated the inverse correlation of the ERK pathway and proliferation by administration of small molecules. While there are no small molecules that work directly on the ERK pathway, other studies have used Fisetin as an agonist and PD98059 as an antagonist (Maher et al., 2011; Alessi et al., 1995). We titrated these small molecules on 16pDel and NIH control NPCs. No consistent change in P-ERK levels or proliferation was observed. These experiments do not support any causal effect between P-ERK levels and cell proliferation (data not shown), even though P-ERK levels are inversely correlated with the type of proliferation defect in four of the five autism NPCs across two different datasets.

### Dysregulation of proliferation extends only to 16pDel iPSCs and not to I-ASD iPSCs

**Figure 7:**
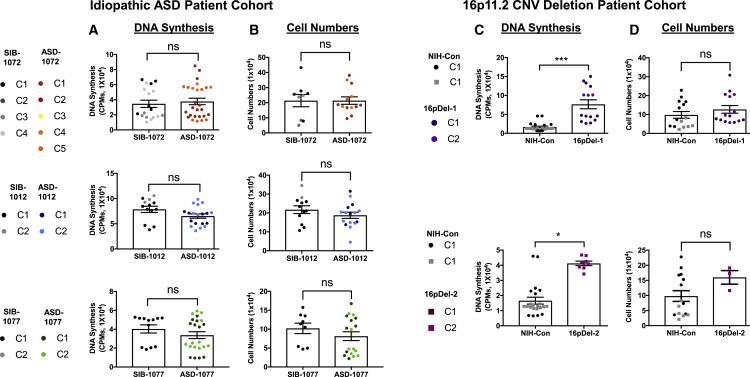
I-ASD iPSCs exhibit no difference in proliferation, whereas 16pDel iPSCs show an increase in DNA synthesis (A and B) Families 1072, 1012, and 1077 I-ASD iPSCs exhibit no differences (A) in DNA synthesis at 48 h (B) or in cell numbers at day 3. (C) 16pDel-1 iPSCs exhibited a 362% increase in DNA synthesis at 48 h, while 16pDel-2 showed a 148% increase in DNA synthesis. (D) Neither 16pDel-1 nor 16pDel-2 iPSCs displayed a significant difference in cell numbers at day 3. Data represent the mean ± SEM. The sample sizes were number of individuals, number of iPSC clones, number of experiments, and number of wells, and the ranges for each individual were control, 1, 1–4, 3–10, and 9–33; and ASD, 1, 1–4, 1–11, and 3–42. See Table S1 for iPSC sample size details (^∗^p < 0.05, ^∗∗∗^p < 0.001).

Finally, we assayed for proliferation phenotypes in additional cell types such as iPSCs. Interestingly, none of the three I-ASD iPSCs displayed significant changes in DNA synthesis at 48 h (Figure 7A) nor in cell numbers at 3 days (Figure 7B) compared with sibling control iPSCs. This suggests that the I-ASD dysregulation of proliferation is not global.

In contrast, proliferation of the iPSCs from the two 16pDel individuals exhibited increased DNA synthesis at 48 h, with 16pDel-1 increasing by 360% and 16pDel-2 by 148% (Figure 7C). This is similar to the 16pDel NPC results (Figure 3A). However, unlike the NPCs, we did not observe an increase in iPSC cell numbers at 3 days in culture (Figure 7D), suggesting that more complex mechanisms may be involved. These data indicate that the 16p deletion affects proliferation more broadly, unlike I-ASD, where the proliferative defects have been observed only in NPCs.

## Discussion

### Dysregulated proliferation is a common phenotype in our sample of five ASD individuals

In this study, we employed a rigorous approach to assess neurogenesis utilizing 53 NPC derivations from 24 distinct iPSC lines of 10 individuals, to compare the proliferation phenotypes between an idiopathic ASD subgroup and the genetically defined 16pDel model. Proliferation defects were observed in all five ASD NPC lines across both subgroups, which is interesting in light of their clinical and genetic heterogeneity. We also uncovered hyperproliferation defects in macrocephalic individuals from both subgroups, with this study being the first to report proliferative defects in 16pDel NPCs. These data suggest that dysregulation of proliferation is a common defect in ASD, manifesting as either “too little” or “too much.”

Our proliferation results are supported by other studies. Proliferation defects have been reported previously using iPSC-derived NPCs for both idiopathic ASD and other CNVs, such as 7q11.23 (Mariani et al., 2015; Marchetto et al., 2017; Chailangkarn et al., 2016; Li et al., 2017; Turkalj et al., 2020; Prem et al., 2020; see Connacher et al., 2018, for a review). Consistent with these data, numerous genetic studies have suggested that altered proliferation of cortical NPCs may be a potential cellular mechanism for autism risk (Krishnan et al., 2016; Packer, 2016; Grove et al., 2019; Satterstrom et al., 2020). For example, 102 high-confidence autism risk genes have been identified, and a majority of them are expressed in embryonic forebrain NPCs at 23 weeks of gestation (Satterstrom et al., 2020). Furthermore, altered proliferation has recently been reported to be a common phenotype when the neurodevelopmental functions of multiple ASD high-confidence risk genes were examined (Willsey et al., 2021). Our study now adds further evidence that NPC proliferation dysregulation is observed in ASD across multiple forms of ASD.

While we observe consistent proliferation phenotypes, the cellular mechanisms are likely to be different between subgroups and ASD individuals. I-ASD NPCs from two families (1072, 1012) with normal head circumference exhibited significantly decreased proliferation. Family 1072 NPCs demonstrated a much greater decrease in proliferation that was paralleled by a reduction in S-phase entry and an increase in cell death, while for family 1012, a smaller decrease in proliferation correlated with a significant increase in cell death, with no difference in cells engaged in S phase. Understanding the mechanisms responsible for the decrease is an issue for future study.

Importantly, our results also add to the mounting evidence that autism NPCs from those with macrocephaly exhibit a hyperproliferation phenotype. While macrocephaly is well described in individuals who carry the 16pDel, this is the first evidence that their derived NPCs exhibit a proliferation phenotype. While both males have the same CNV deletion, they exhibit differing levels of increased proliferation, highlighting the heterogeneity of autism even within a genetically defined subgroup. Although the macrocephalic I-ASD family 1077 individual also displayed hyperproliferation, the mechanism is likely different. Indeed, compared with respective controls, family 1077 NPCs had no changes in the levels of P-ERK1 nor in NPC molecular markers, while 16pDel exhibited many mRNA differences and decreased P-ERK. Further, our findings of increased proliferation in 16pDel NPCs are in contrast to a previous study that did not find changes in proliferation or S-phase entry (Deshpande et al., 2017). While exact causes remain undefined, there are a variety of methodological differences between the studies, including iPSCs from different 16pDel individuals, NPC derivation protocols, culture duration/passage, and medium composition, that could explain the difference.

The idea that either too little or too much of a neurodevelopmental process may contribute to autism neuropathogenesis has broad support across multiple studies. For example, both deletion and duplication lead to neurodevelopmental phenotypes of Rett syndrome and MECP2 duplication syndrome (Ramocki et al., 2009); the FXS-related gene, FMR1 (Auerbach et al., 2011; Hickey et al., 2013; Arsenault et al., 2016); and the 16p11.2 CNV (Horev et al., 2011; Niarchou et al., 2019). Further, mirror cellular and synaptic phenotypes have also been commonly reported in autism. For example, bidirectional changes in both dendritic spine densities and cell numbers in specific brain regions have been shown in multiple neuropathological studies of idiopathic autism (Amaral et al., 2008; Hutsler and Zhang, 2010; Wegiel et al., 2010, 2014; Varghese et al., 2017).

### Altered mitogenic responses correlate inversely with baseline proliferation

NPCs from all idiopathic and 16pDel ASD cases displayed altered mitogenic responsiveness to bFGF, an important regulator of neurodevelopment (Tao et al., 1996; Wagner et al., 1999; Cheng et al., 2001, 2002; Li and DiCicco-Bloom, 2004; Stevens et al., 2010). Notably, the altered mitogenic response was inversely correlated with baseline NPC proliferation. In the hypoproliferative group, ASD individuals (1072, 1012) displayed an increased sensitivity and response to bFGF stimulation, whereas faster proliferators (16pDel-1 and -2, family 1077) displayed a blunted response to bFGF stimulation. This may suggest a couple of different mechanisms: (1) a ceiling effect whereby hyperproliferating NPCs are already proliferating at a high rate and cannot be further stimulated or (2) alterations in the FGF receptors or downstream signaling cascade that could either enhance or dampen pathway activation. bFGF has pleiotropic effects during development and on stem cell proliferation that can depend on growth factor concentrations (Garcia-Maya et al., 2006). One target of FGF receptor activation, membrane-linked docking/scaffolding adaptor protein FRS2α, determines the degree to which FGF activates MAP/ERK kinases and PI3K pathways. This protein is essential for FGF-induced MAP/ERK kinase stimulation and proliferation in response to low (but not high) growth factor concentrations (Hadari et al., 2001), and it regulates neurogenesis in the embryonic forebrain ventricular zone (Sato et al., 2010). These and other downstream regulators of bFGF activity will be fertile ground for future studies of mechanisms underlying NPC mitogenic responses and proliferation.

### Potential role of levels of phosphorylated ERK1 in proliferative phenotypes

Alterations in signaling pathways often underpin proliferative changes in neurodevelopmental disorders. While many pathways are relevant, gene ontology studies from the Simons Foundation Autism Research Initiative (SFARI) and others have identified calcium and MAPK signaling as central nodes in ASD (Wen et al., 2016; Levitt and Campbell, 2009; Packer, 2016). Mutations in components of the MAPK signaling pathway, termed the RASopathies, produce numerous neurodevelopmental disorders, including autism (Adviento et al., 2014; Levitt and Campbell, 2009), and the *MAPK3* gene has been suggested to contribute to the head size changes in 16p11.2 individuals (Shinawi et al., 2010).

Given the proposed role of *MAPK3*/ERK1 deletion in the 16p11.2 CNV phenotype, we examined potential contributions to ASD NPC proliferation by measuring P-ERK1/2 protein levels. Our studies indicate that in four of the five ASD individuals, the absolute levels of P-ERK1 correlated inversely with the levels of NPC proliferation. In hypoproliferative I-ASD NPCs, we observed increased levels of P-ERK1 protein at baseline, a change that may underlie their increased sensitivity to bFGF stimulation. Conversely, in hyperproliferative NPCs from 16pDel individuals, we observed ∼50% reduction in P-ERK1 levels, as expected given the hemizygous genetic status of CNV deletion carriers. This reduction in absolute P-ERK1 protein levels may potentially contribute to the blunted responses to bFGF in the 16pDel cohort. It is notable that, unlike the 16pDel mouse model (Pucilowska et al., 2015), we did not detect a change in the P-ERK1/total ERK1 protein ratio. Classically, the tyrosine kinase field considers intracellular signaling to depend primarily on the ratio of P-ERK protein to total ERK protein (normalized to total ERK), when describing levels of activity. However, conceptually, cellular signaling and response may also depend on the absolute amount of P-ERK1, because this may have an impact on signaling dynamics and kinetics, as well as complex interpathway interactions (Ersahin et al., 2015). Of note, additional experiments using ERK agonist Fisetin or the ERK antagonist PD98059 did not alter P-ERK levels in a predictable way and did not support the concept that alterations in P-ERK1 levels directly contribute to dysregulated proliferation in the ASD cases examined. However, this is only one of many mechanisms by which proliferation could be altered. Within 16pDel, several other genes (e.g., KCTD13, MVP) have also been suggested to play roles (Golzio et al., 2012; Escamilla et al., 2017; Arbogast et al., 2019; Kizner et al., 2020). Future studies using pharmacologic and molecular tools can investigate where the signaling dysregulation originates and the potential functional significance of P-ERK1 differences to the NPC proliferation phenotypes.

### Proliferative abnormalities are differentially expressed in the iPSCs of distinct ASD subgroups

While we observed proliferative dysregulation in all NPCs, this was not the case for the iPSC clones from which the NPCs were derived. In the three I-ASD families, iPSC proliferation was not different between ASD probands and unaffected sibling controls. In contrast, in 16pDel families, iPSC DNA synthesis was increased compared with the controls. While sample sizes were limited, these initial studies raise the possibility that molecular mechanisms that contribute to ASD may be active at different stages during development yet produce a common final phenotype. That is, in some cases, the defects may manifest within the neural lineage, such as suggested here for I-ASD, whereas in other cases, such as the 16p11.2 CNV, abnormalities may affect multiple developmental stages, cell lineages, and organ systems. Such multi-cell lineage dysfunction is also supported by clinical studies. In 16pDel individuals, ASD, epilepsy, spinal anomalies, diverse organ system congenital defects, and obesity are highly penetrant comorbid features (Shinawi et al., 2010; Steinman et al., 2016), suggesting this CNV affects multiple cell lineages and types.

### Conclusions

In summary, we used a multi-tiered strategy and a rigorous and reproducible sample design to characterize neurodevelopment in ASD patient-derived cells. We identified alterations in proliferation and FGF mitogenic responses in NPCs from both idiopathic and genetic autism cohorts, suggesting that dysregulated proliferation may be a common phenotype in multiple forms of autism. Further, we identified for the first time dysregulation of proliferation in 16pDel NPCs, and provided additional evidence supporting a correlation of hyperproliferation in iPSC-derived cells with patient macrocephaly. The 16p11.2 genetic form of ASD may also have a more broadly disrupted process of proliferation, as abnormalities are observed in both iPSCs and NPCs, results that differ from those in I-ASD. Overall, these observations suggest that disruption of proliferation control during development may be one mechanism contributing to ASD pathogenesis.

## Experimental procedures

### iPSC generation and culture conditions

I-ASD iPSC lines were created by Dr. Lu as reported previously (Yi et al., 2012; Wu et al., 2012). CD4^+^ T cells were infected and reprogrammed using standard Sendai methods (Seki et al., 2012; see supplemental information for details).

### iPSC-derived neural precursor cell generation and culture conditions

NPCs were generated using neural expansion medium (Thermo Fisher, A1647801) as detailed in Williams et al. (2018) (see supplemental information for details). To verify NPC derivation, newly induced NPCs from each clone were routinely immunostained for precursor markers (Sox2, Pax6, Nestin) (see Figure S2) before use and were excluded if Nestin and/or Sox2 expression was < 85%, Pax6 was < 60%, or NPCs expressed the iPSC marker Oct4 or glial proteins (GFAP, S100b).

### Proliferation assays and protein quantitation

Proliferation and apoptosis assays (^3^H labeling, cell counting, EdU, and activated caspase-3 ICC) were performed as detailed in Williams et al. (2018). All NPC line comparisons, which used multiple iPSC clones and NPC derivations, were performed in parallel cultures or in the same week, using the same reagents and starting cell densities, to minimize technical variations (see supplemental information for details).

Western blotting was performed as described previously (Mairet-Coello et al., 2009). NPCs (1 × 10^6^ cells/dish) from passages P3 to P8 were plated in 35 mm dishes and analyzed at 48 h (details provided in the supplemental information).

### NPC mRNA Quantiplex expression analysis

RNA was isolated from I-ASD, 16pDel, and control NPCs using standard protocols, and 250 ng of total RNA was used for mRNA expression analysis. A QuantiGene Plex Assay that included 24 genes (21 genes involved in NPC proliferation and 3 controls) was designed and ordered through Invitrogen and used with standard protocols on triplicate samples (see supplemental information).

### Statistics

Statistical Package GraphPad Prism version 7.0d (GraphPad Software, San Diego, CA, USA) was employed. Statistical testing involved two-tailed Student’s t tests or two-way ANOVA with Sidak’s multiple comparison test where specified. Data are expressed as the mean ± SEM Outliers were determined with the ROUT test and removed from analysis. In each figure legend, the number of iPSC clones used, number of total NPC derivations, number of experiments, and number of wells are detailed for each individual. For all statistical tests, sample size was the number of individual data points including all technical and biological replicates (total number of wells).

### Data and code availability

Approved researchers can obtain the Simons Searchlight population dataset described in this study (https://www.sfari.org/resource/simons-searchlight/) by applying at https://base.sfari.org.

## Author contributions

Conceptualization, J.H.M., E.D.-B., J.F., L.B., C.W.L., and Z.P.P.; methodology, J.H.M., P.M., P.L.Y., E.D.-B., and M.W.; formal analysis, M.W., R.C., S.P., P.M., P.L.Y., and M.M.; investigation, R.C., M.W., S.P., M.M., P.L.Y., C.W.L., A.M., C.P., X.Z., and C.R.M.; writing – original draft, M.W., R.C., M.M., J.H.M., and E.D.-B.; visualization, M.W., R.C., S.P., and M.M.; writing – review & editing, M.W., R.C., M.M., J.H.M., and E.D.-B.; funding acquisition, J.H.M. and E.D.-B.; resources, J.F., L.B., C.W.L., and P.L.Y. created and validated all I-ASD and sib iPSC lines used in this study, and NPC lines were generated by P.L.Y., P.M., M.M., S.P., M.W., and R.C.; supervision, J.H.M., E.D.-B., P.M., and C.W.L.

## CONFLICT OF INTEREST

The authors declare no competing interests.

## Supplemental information

Document S1. Figures S1–S6, Table S1, and supplemental experimental procedures

Document S2. Article plus supplemental information
