# Autism NPCs from both idiopathic and CNV 16p11.2 deletion patients exhibit dysregulation of proliferation and mitogenic responses

**DOI:** 10.1016/j.stemcr.2022.06.007

**Published:** 2022-07-12

**Authors:** Robert Connacher, Madeline Williams, Smrithi Prem, Percy L. Yeung, Paul Matteson, Monal Mehta, Anna Markov, Cynthia Peng, Xiaofeng Zhou, Courtney R. McDermott, Zhiping P. Pang, Judy Flax, Linda Brzustowicz, Che-Wei Lu, James H. Millonig, Emanuel DiCicco-Bloom

## Main Text

(Stem Cell Reports *17*, 1380–1394; June 14, 2022)

In the Highlights section of our originally published manuscript, highlight number 3 was incorrect upon initial submission, whereas the manuscript itself was and remains otherwise entirely correct. This highlight stated, "NPC proliferative responses to bFGF correlate inversely with P-ERK levels." This bullet point was intended to highlight the inverse correlation of proliferation and P-ERK levels and has been changed to, "NPC proliferation correlates inversely with P-ERK levels."

