# Supplementary material for: Autism NPCs from both idiopathic and CNV 16p11.2 deletion patients exhibit dysregulation of proliferation and mitogenic responses
Source: Stem Cell Reports. 2022 May 26;17(6):1380–94. doi: 10.1016/j.stemcr.2022.04.019 (PMC9214070; doi:10.1016/j.stemcr.2022.04.019)
Supplement: Document S1. Figures S1–S6, Table S1, and supplemental experimental procedures [file mmc1.pdf]

Stem Cell Reports, Volume *17*

Supplemental Information

Autism NPCs from both idiopathic and CNV 16p11.2 deletion patients exhibit dysregulation of proliferation and mitogenic responses

Robert Connacher, Madeline Williams, Smrithi Prem, Percy L. Yeung, Paul Matteson, Monal Mehta, Anna Markov, Cynthia Peng, Xiaofeng Zhou, Courtney R. McDermott, Zhiping P. Pang, Judy Flax, Linda Brzustowicz, Che-Wei Lu, James H. Millonig, and Emanuel DiCicco-Bloom

SUPPLEMENTAL INFORMATION

Supplemental Figures

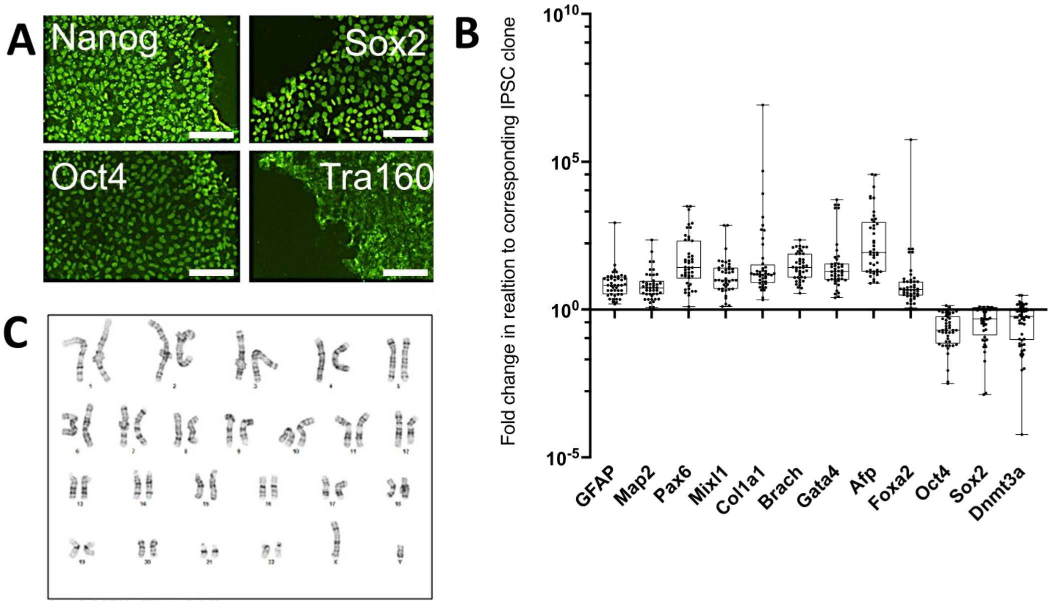

Figure S1. Characterization of iPSCs. Related to Figure 1: A) iPSCs colonies were immunostained for pluripotency markers (Tra-160, Nanog, Oct4, Sox2) as shown for a representative I-ASD individual. Scale bars: 50 µm B) Embryoid body (EB) Quanti-Plex panel demonstrates that cells express mature lineage markers for endo-, meso- and ectoderm (*GFAP*, *MAP2*, *PAX6*, *MIXL1*, *COL1A1*, *BRACH*, *GATA4*, *AFP*, *FOXA2*) and down-regulate pluripotency genes (*DNMT3A*, *OCT4*, *SOX2*). These results indicate that the iPSC clone is pluripotent. C) Representative normal karyotype of I-ASD iPSC clone.

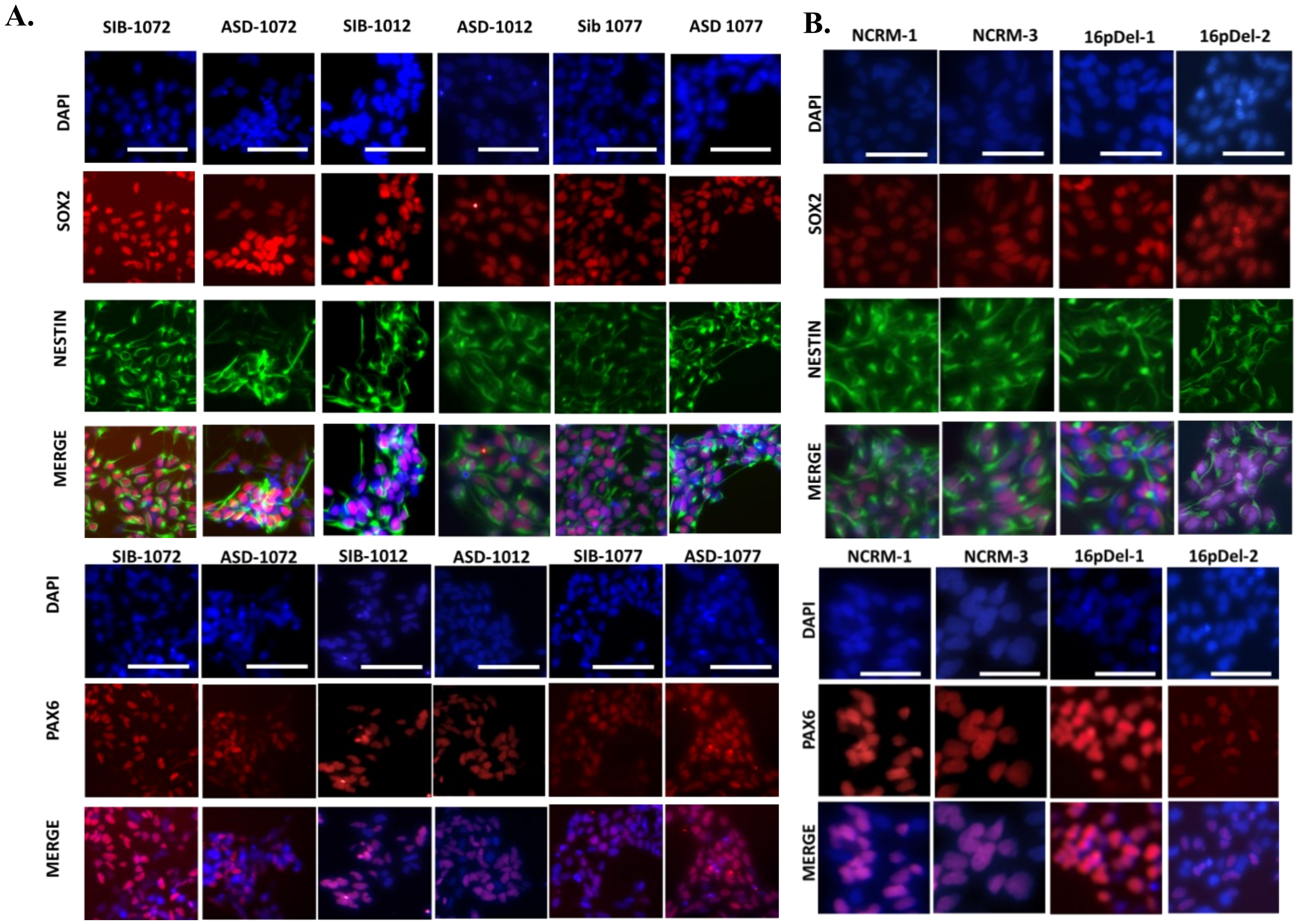

Figure S2. Representative ICC images of NPCs from control and ASD NPCs characterizing precursor marker expression. Related to Figure 1:

A and B) To ensure rigor and reproducibility, each NPC induction was routinely immunostained (100,000 NPCs per 24-well plate) with a series of NPC markers (Sox2, NESTIN, PAX6). In A, 20x magnification representative images of I-ASD affected and control sibs for families 1072, 1012, and 1077 are shown. Images for all three markers are presented along with DAPI nuclear staining and appropriate merged images. In B, identical immunostainings are shown for the 16pDel datasets (16pDel-1 and -2) and NIH controls (NCRM-1 and -3). Note: Brightness of images was increased to better visualize cells. Scale bars: 50 µm.

A.

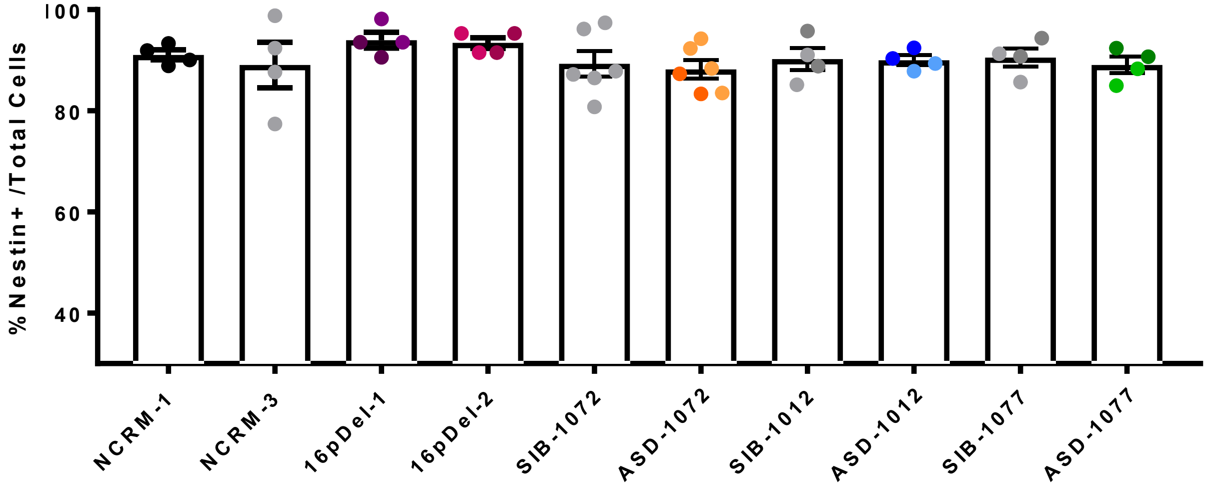

B.

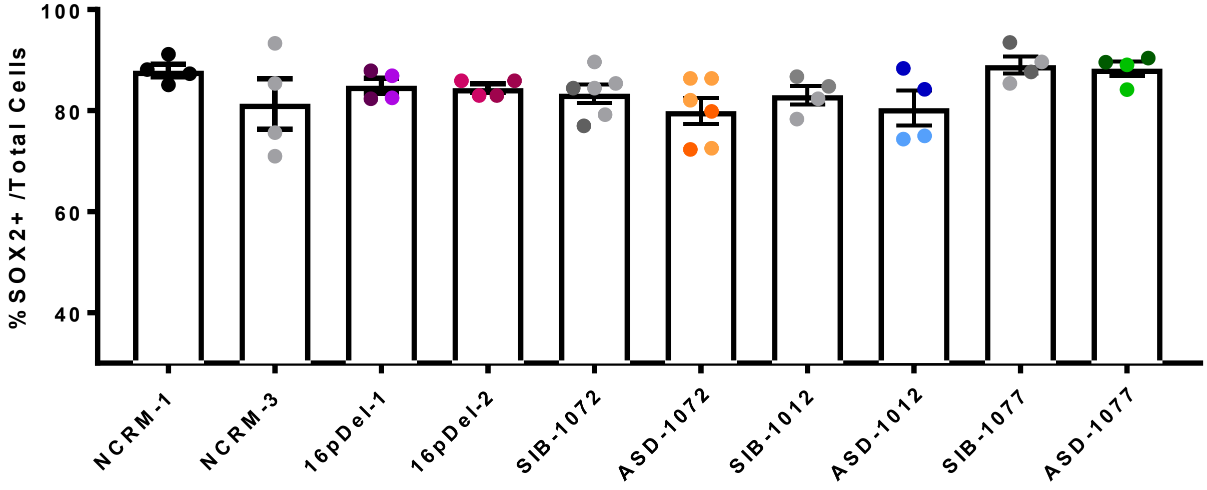

Figure S3. ICC Quantification for Nestin and SOX2 expression in control and ASD NPCs. Related to Figure 1 & Table S1:

A) Quantification of ICC images stained for Nestin. B) Quantification of ICC images stained for SOX2. All experiments were immunostained 48 hrs. Assessment of ICC stain from 35 mm dishes (50,000 cells/dish) was conducted for two clones per person and two dishes per clone for a total of 4 dishes per individual. NCRM-1 and NCRM-3 were limited to 1 clone per person and served as normal controls for 16pDel individuals. Cell counts were assessed at 20x magnification in 3 - 1cm horizontal strips across the upper, middle, and lower thirds of each dish with the 3 values analyzed as mean +/- SEM. See **Table S1** for iPSC and NPC sample size details.

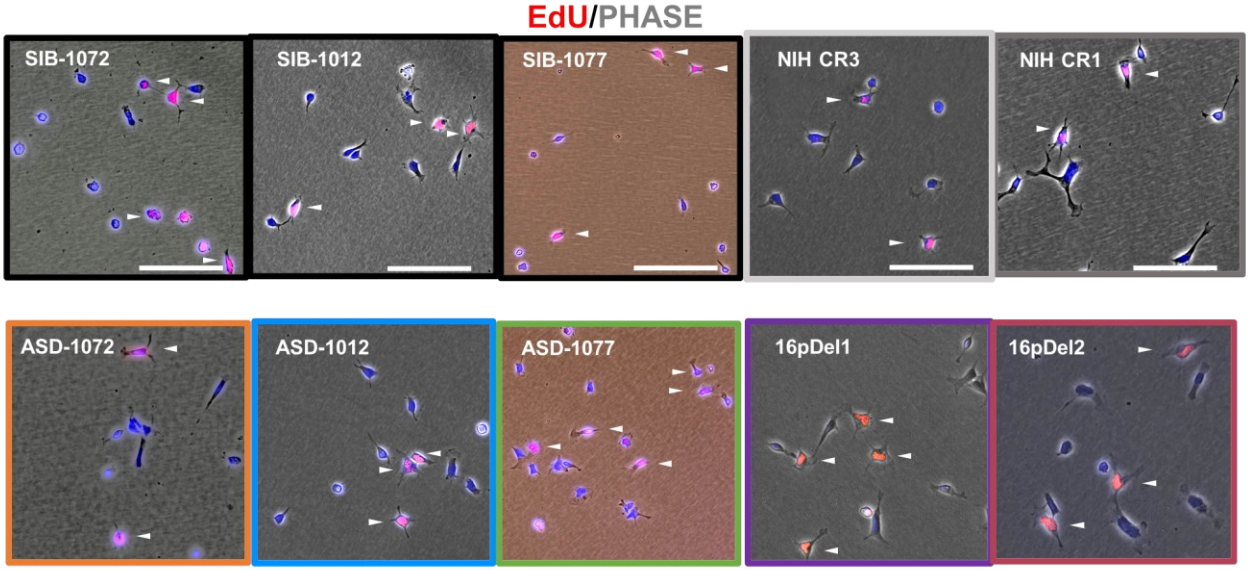

Figure S4. Representative phase/ EdU images from control and ASD NPCs. Related to Figures 2, 3 & Table S1.

Images of merged Phase/EdU labeling for Sib control, I-ASD, NIH control, and I6pDel NPCs demonstrating altered S phase labeling for I-ASD and 16pDel NPCs (bottom row) compared to controls (top row)(I-ASD: Families 1072, 1012 and 1077; 16pDel: 16pDel-1 &-2) in 35 mm plates (100,000 cells/ dish) at 48 hrs. White arrowheads denote viable EdU (Red) positive NPCs in each image, counterstained with DAPI (Blue). Note: Brightness of images was increased to better visualize cells. Scale bars: 50 µm. Please refer to Figure 2 C, G, K and Figure 3C for corresponding quantification of EdU experiments. See **Table S1** for iPSC and NPC sample size details.

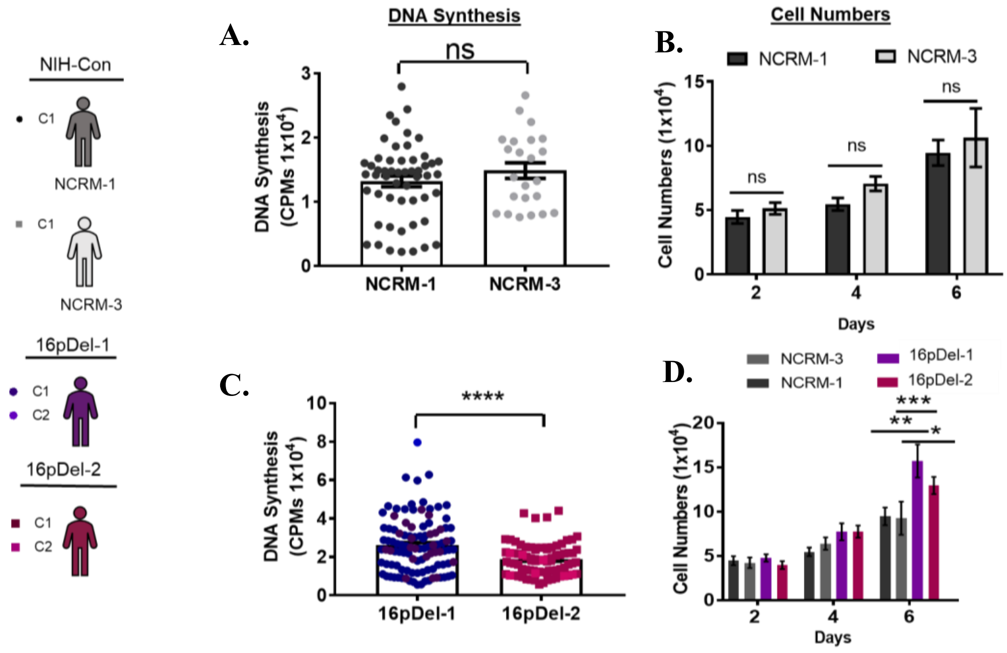

<u>Figure S5. No differences in proliferation observed between NPCs from two NIH controls yet patient specific differences are observed in 16p11.2 CNV deletion NPCs. Related to Figure 3 and Table S1:</u>

A) NPCs derived from NIH controls lines (NCRM-1 and NCRM-3) display non-significant differences in DNA synthesis and B) Enumeration of cell numbers at 48h. C) 16pDel-1 and 16pDel-2 NPCs exhibit a significant difference at 48h in DNA synthesis under untreated culture conditions D) Enumeration of cell numbers at 48h reveal significant increases of 16pDel NPCs to individual controls and between 16pDel-1 and 16PDel-2 by 6 days. See **Table S1** for iPSC and NPC sample size details.

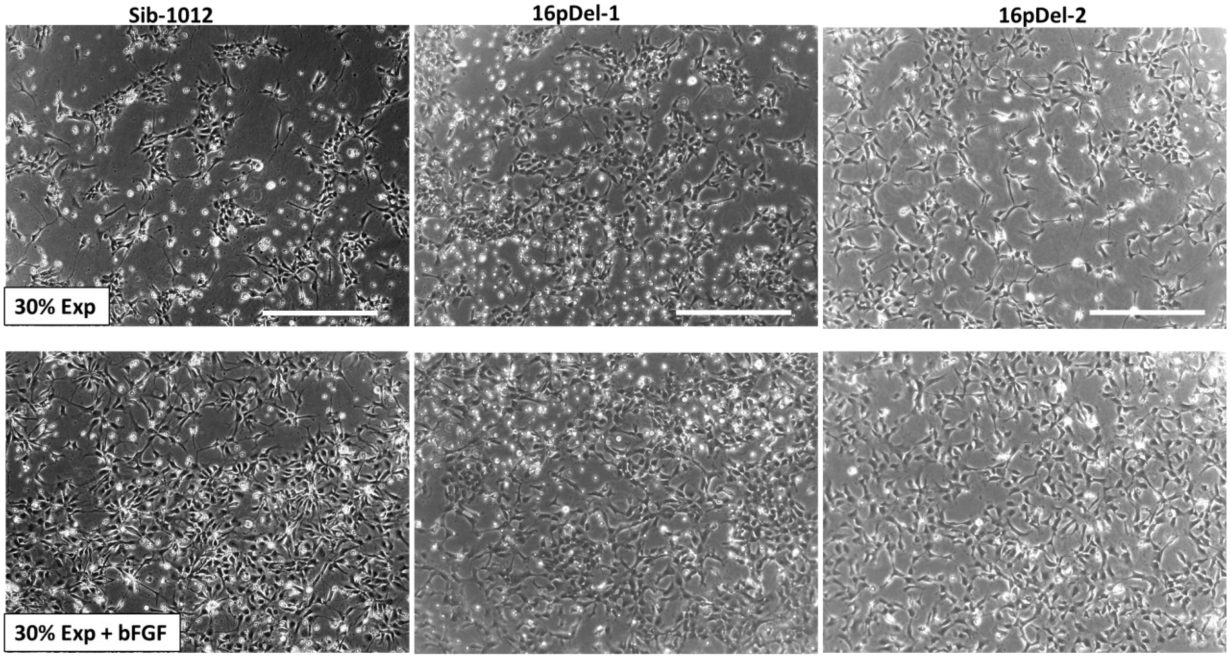

Figure S6. Representative phase images from control and ASD NPCs demonstrating that the cells do not reach confluency in the absence or presence of bFGF prior to 3H incorporation analysis. Related to Figure 4.

Phase images of Sib and 16pDel NPCs grown in 24 well plates are shown at 48hrs, demonstrating NPC density immediately prior to harvesting to assess 3H incorporation levels. Representative phase images of Sib-1012, 16pDel-1 and 16pDel-2 NPCs demonstrate that NPCs did not reach confluency +/- bFGF in 30% Expansion media. Scale bars: 250 µm

Supplemental Table 1:

Figure	Subfigure				
	**A**	**B**	**C**		
**2**	SIB-1072<br>n=1/4/5/31/105	SIB-1072<br>n=1/4/5/18/39	SIB-1072<br>n=1/4/5/11/32		
	ASD-1072<br>n=1/5/9/36/118	ASD-1072<br>n=1/5/9/24/51	ASD-1072<br>n=1/4/5/9/24		
	**D**	**E**	**F**		
	SIB-1072<br>n=1/2/2/6/17	SIB-1012<br>n=1/2/5/21/106	SIB-1012<br>n=1/2/5/9/18		
	ASD-1072<br>n=1/2/3/5/14	ASD-1012<br>n=1/2/8/14/56	ASD-1012<br>n=1/2/6/7/14		
	**G**	**H**	**I**		
	SIB-1012<br>n=1/1/1/2/5	SIB-1012<br>n=1/2/3/4/12	SIB-1077<br>n=1/2/5/25/74		
	ASD-1012<br>n=1/1/1/2/6	ASD-1012<br>n=1/2/3/4/12	ASD-1077<br>n=1/3/4/9/27		
	**J**	**K**	**L**		
	SIB-1077<br>n=1/2/2/12/24	SIB-1077<br>n=1/2/2/5/15	SIB-1077<br>n=1/2/2/6/18		
	ASD-1077<br>n=1/3/4/8/16	ASD-1077<br>n=1/2/2/2/6	ASD-1077<br>n=1/2/2/5/15		
**Figure**	**Subfigure**				
	**A**	**B**	**C**	**D**	**E**
**3**	NIH Con<br>n=2/2/9/21/75	NIH Con<br>n=2/2/5/8/16	NIH Con<br>n=2/2/4/5/15	NIH Con<br>n=2/2/2/3/9	
		(NCRM-1 n= 1/1/3/4/8, NCRM-3 n= 1/1/2/4/8)			
	16pDel-1<br>n=1/2/3/31/108	16pDel-1<br>n=1/2/4/7/14	16pDel-1<br>n=1/2/3/5/14	16pDel-1<br>n=1/2/2/5/15	
	16pDel-2<br>n=1/2/4/23/81	16pDel-2<br>n=1/2/3/5/10	16pDel-2<br>n=1/2/5/8/24	16pDel-2<br>n=1/2/3/4/12	
**4**	SIB-1077<br>n=1/2/3/6/16	NIH Con: NCRM-1<br>n= 1/1/2/3/10	NIH Con:<br>NCRM-1 n=<br>1/1/2/3/10	SIB-1072<br>n=1/2/2/4/13	SIB-1012<br>n=1/2/2/6/18
		NIH Con: NCRM-3<br>n=1/1/2/3/9	NIH Con:<br>NCRM-3<br>n=1/1/2/3/9		
	ASD-1077<br>n=1/2/3/5/14	16pDel-1<br>n=1/2/3/6/23	16pDel-2<br>n=1/2/3/6/23	ASD-1072<br>n=1/3/4/5/14	ASD-1012<br>n=1/2/2/5/21
**5**	SIB-1072: 3 iPSC clones	SIB-1072: 3 iPSC clones	NIH: 2 individuals	NIH: 2 individuals	
	ASD-1072: 3 iPSC clones	ASD-1072: 3 iPSC clones	16pDel-1: 2 iPSC clones	16pDel-2: 3 iPSC clones	
**6**	NIH Con<br>n=2/2/4/2/4	SIB-1077 n=1/2/5/4/8	SIB-1072<br>n=1/3/3/4/5	SIB-1012<br>n=1/2/3/2/4	

	16pDel-1 n=1/2/2/4/6	ASD-1077 n=1/4/4/4/6	ASD-1072 n=1/4/4/4/5	ASD-1012 n=1/2/4/2/4	
	16pDel-2 n=1/2/2/4/6				
**7**	SIB-1072 n=1/4/10/33	SIB-1072 n=1/4/11/23	NIH Con n=2/2/7/27	NIH Con n=2/2/5/15	
	SIB-1012 n=1/2/4/13	SIB-1012 n=1/2/4/12			
	SIB-1077 n=1/1/4/12	SIB-1077 n=1/1/3/9	16pDel-1 n=1/2/4/15	16pDel-1 n=1/2/5/15	
	ASD-1072 n=1/4/11/42	ASD-1072 n=1/4/10/21			
	ASD-1012 n=1/2/5/19	ASD-1012 n=1/2/5/16	16pDel-2 n=1/1/2/8	16pDel-2 n=1/1/1/3	
	ASD-1077 n=1/2/6/23	ASD-1077 n=1/2/6/18			

Table S1. Tabulation of NPC and IPSC experiments. Relating to Figures 2-7: For cells relating Fig 2-6, The n-values represent the numbers of individuals/# of iPSC clones/# of NPC derivations/# of experiments/# of wells. For cells relating to Fig 7, The n-values represent the numbers of individuals/# of iPSC clones /# of experiments/# of wells. Bolded cells indicate Figures and Subfigures.

Supplemental Experimental Procedures

Dataset information

Idiopathic Autism (I-ASD)

I-ASD iPSCs were derived from the New Jersey Language and Autism Genetics Study (NJLAGS) dataset generated by the lab of Linda Brzustowicz MD. Recruited NJLAGS families have at least one family member diagnosed with autism and at least one other family member diagnosed with Language-based Learning Impairment (LLI). Delays in other domains are not observed clinically. All family members were diagnosed by the same clinical team using the same validated clinical instruments. Autism was diagnosed by ADOS and ADI-R and DSM-IV, while LLI was diagnosed by Clinical Evaluation of Language Fundamentals-4, Comprehensive Test of Phonological Processing, Gray Oral Reading Tests-4, Woodcock Reading Mastery Tests-Revised and Weschler Abbreviated Scale of Intelligence or the Developmental Abilities Scale (Bartlett et al., 2012, Bartlett et al., 2014). Unaffected family members were clinically evaluated but did not reach diagnostic criteria for autism or LLI.

In examination of clinical phenotypes, ASD-1072 has severe cognitive impairment; an IQ test was attempted but not completed. ADOS and ADI-R revealed language comprehension limited to a small number of single words as well as occasional echolalic/scripted speech. The Social Response Scale (SRS) score was 90 denoting a severe social impairment. ASD-1072 had a head circumference at the 78th percentile at the time of measurement (4.1 years) indicating a head size in the normal range. For Family 1012, ASD-1012 has comprehension limited to a small number of single words, a nonverbal IQ (NVIQ) of 118, and an SRS score of 69 (mild/moderate range of social impairment). No data was available for the head circumference of Family 1012 proband (Figure 1C). For Family 1077, ASD-1077 has severe cognitive impairment; an IQ test was attempted but not completed. ADOS and ADI-R revealed language comprehension limited to single words and directions that are part of his routines with almost no language production. The SRS score was 83 (severe social impairment). ASD-1077 had a head circumference of 97th percentile at the time of measurement (14.06 years), which is consistent with a diagnosis of macrocephaly. Unaffected individuals were diagnostically determined to not have any language/learning impairment or ASD phenotype.

16pDel Dataset

For the 16pDel cohort, iPSCs were available from RUCDR Infinite Biologics for two males. These two male individuals were chosen from a larger cohort of 115 deletion carrier families within the Simons Foundation Autism Research Initiative (SFARI), Simons Variation in Individual project (Simons VIP) collection (Simons Vip, 2012), now renamed as Simons Searchlight. ASD inclusion criteria for 16pDel probands required they meet ADI-R and ADOS score cutoff criterion for autism spectrum disorder or autism (some individuals were clinically assessed using DSM-IV criteria). The probands were assessed on verbal and nonverbal cognitive abilities as described previously (Simons Vip, 2012). The first male (16pDel-1) exhibited Autism Spectrum Disorder (Asperger's Disorder), with a full scale IQ (FSIQ) of 122, (non-verbal IQ [NVIQ] 130, verbal IQ [VIQ] 106), head circumference at the 99th percentile at 14.5 years, consistent with macrocephaly, and comorbid expressive language disorder, anxiety, and microphthalmia with a SRS score of 76. The second male (16pDel-2) exhibited autism, with FSIQ score of 93 (NVIQ 98, VIQ 87) and a head circumference at the 99th percentile at age 14.3 years which is consistent with a macrocephaly diagnosis. He also displayed numerous other developmental phenotypes including coordination disability, developmental delay, cerebral palsy, ADD/ADHD, articulation disorder, and repetitive/expressive language disorder and a SRS score of 90.

Two iPSC clones per 16pDel individual were obtained from RUCDR Infinite Biologics. Given that genetically matched siblings for 16pDel individuals were not available, two sex matched research grade iPSC control stem cell lines (NCRM-1 and NCRM-3) were obtained from the NIH Regenerative Medicine Program (RMP) via RUCDR Infinite Biologics. iPSCs were generated from CD34+ umbilical cord blood cells from individuals who were karyotypically normal at birth, using episomal plasmid reprogramming methods. Only one iPSC clone was available for each NIH control, but a minimum of two NPC inductions were conducted for rigor and reproducibility. Subsequent cellular analyses for all assays revealed that NCRM-1 and NCRM-3 were not statistically different from one another (Supplemental Figure 5). Thus, all 16pDel comparison studies in this paper were made against a compilation of both NIH controls, specified as "NIH-Con".

iPSC generation and culture conditions

To generate I-ASD iPSCs, cryopreserved CPLs from the NJLAGS dataset were obtained from RUCDR. CPLs were thawed, grown in RPMI, 15% heat inactivated FBS, 1x glutamine and 1x Pen/Strep. $CD4^+$ T cells were negatively selected using Dynabead Untouched Human CD4 T cell kit (Life Technologies). The remaining $CD4^+$ T cells were then activated and expanded with CD3/CD28 Dynabeads (Life Technologies) with 250U/ml of IL2 for 2-3 days. 80,000 $CD4^+$ T cells were then infected and reprogrammed using a non-integrating Sendai virus expressing hOct4, hSox2, hKlf4, and hc-Myc (CytoTune) as specified by manufacturer's instructions (Seki et al., 2012). Infection occurred for 24 hours and then infected cells were plated onto irradiated MEFs and grown in KOSR media (DMEM/F12, Knockout serum replacement, non-essential amino acids, 1x glutamine and 20ng/ml human bFGF) for 2-4 weeks.

After 2-3 weeks iPSC colonies were readily visible for the affected and unaffected same sex sibling for the three I-ASD families (Family 1072, Family 1077, and Family 1012). Colonies were then picked and plated in 1x Geltrex (Thermofisher) coated wells, cultured in mTeSR media (Stemcell Technologies), passed two times and then cryopreserved. 5-50 clones were frozen for each of the six individuals. For subsequent phenotyping/neural differentiation, 3 clones from each individual were expanded, cells undergoing spontaneous differentiation were removed from each clone and the clones were passed for at least 10 passages (P10) to ensure removal of the non-integrating reprogramming Sendai virus.

To maintain iPSC lines, cells were cultured on Matrigel (Corning, 354277) and in mTeSR™1 media (Stem Cell Technologies, 85850) that was changed daily. After cells reached 70–90% confluence, they were treated with 0.5 mM EDTA diluted in 1XPBS for a minimum of 10 min. When iPSCs lifted, they were centrifuged at 150xg for 5 min, re-suspended in media, and plated at 250,000 cells/6 well plate. For the first 24h, cells were incubated with 10µM ROCK Inhibitor, Y27632 solution (Stem Cell Technologies, 72302).

To verify the pluripotency of the iPSC lines, we first performed live Tra-160 ICC after P5 followed by staining for additional iPSC markers (Tra-160, Sox2, Nanog, Oct4) on fixed cells after P10 (see Immunocytochemistry section). EB assays were then performed using standard procedures. Briefly, EBs were formed using aggre-well plates (Stem Cell Technology) and then transferred to untreated tissue culture wells. EBs were grown in EB formation media (Stem Cell Technology) and differentiated for 2-3 weeks. Next QuantiPlex mRNA expression analysis was performed for ectoderm, mesoderm, endoderm and iPSC markers (*GFAP*, *MAP2*, *PAX6*, *MIXL1*, *COL1A1*, *BRACH*, *GATA4*, *AFP*, *FOXA2*, *OCT4*, *SOX2*, *DNMT3A*). Clones were karyotyped or CGH arrays were performed and demonstrated no microscopic rearrangements are observed. For our studies iPSC clones only up to P20 were used.

16p11.2 iPSC lines were derived from CPLs and fibroblasts and RUCDR reprogrammed these cells using episomal (16pDel-1) or Sendai virus 2.0 methods (16pDel-2)(Simons Vip, 2012) respectively. Given that genetically matched siblings for 16pDel individuals were not available, sex matched iPSC lines were obtained from the NIH Regenerative Medicine Program.

Generation of hiPSC-derived neural precursor cells and culture maintenance
To generate NPCs from iPSCs, 2.5 – 3.0 x 10^4 cells/cm^2 iPSCs were plated in one well of a 6 well plate in mTeSR™1 media with 5 µM Y27632 solution for 24h. After 24h, media was replaced with Neural Induction Media (ThermoFisher Scientific, A1647801). Media was changed every other day for 7 days. After 7 days, cells were passaged and considered passage 0 (P0). For more information see manufacturers protocol and (Williams et al., 2018): ThermoFisher: GIBCO Induction of Neural Stem Cells from Human Pluripotent Stem Cells Using PSC Neural Induction Medium: https://tools.thermofisher.com/content/sfs/manuals/MAN0008031.pdf

In order to maintain lines, cells were cultured on Matrigel and maintained in Neural Expansion Media (ThermoFisher Scientific, A1647801). Media was changed every 48h until cells reached confluence at which time they were dissociated using Accutase (Gibco, A11105-01) at 37°C for 10 min, re-suspended with 1XPBS (3 – 5 mL), centrifuged at 300×g for 5 min, re-suspended in media, and counted before plating at a density of 1.0 - 1.5x10^5 cells/cm^2 per well of a 6 well plate. For the first 24h of passages 0 – 3, cells were incubated with 5 µM Y27632 solution.

In order to establish Quality Control (QC) of the iPSC-derived NPCs, we routinely performed immunostaining for Nestin, Pax6, and Sox2 for each induction and clone as a requirement for using them in experiments. QC was performed on passage 3 cells plated at 100,000 cells/24 well plates, as shown in Figures S2. Additional QC of NPCs

was also performed by quantifying cells at low density (50,000 cell/ 35 mm dish), as shown as S3 above (see ICC below for protocol). NPCs were discarded if marker immunostaining revealed Nestin or Sox2 <85% or Pax6 < 60%. Quantification of total cells (DAPI positive) as well as cells immunopositive for aforementioned marker(s) was assessed at 20x magnification in 3 – 1 cm horizontal strips across the upper, middle, and lower thirds of each dish/well. A mean +/- SEM was then calculated. For approved clones, continued monitoring of NPCs was performed and if changes in cellular morphology or cell growth were observed, then the ICC staining was conducted again at the later passage. Any NPCs failing the above immunostaining criterion were discarded.

In order to differentiate NPCs into neurons, oligodendrocytes, or astrocytes, NPCs were cultured according to manufacturers instructions www.thermofisher.com/us/en/home/references/protocols/neurobiology/neurobiology-protocols/differentiating-neural-stem-cells-into-neurons-and-glial-cells.html
Briefly NPCs were differentiated into neurons by culturing in 1x Neurobasal media, 2% B-27 serum free supplement and 2mM GlutaMAX-I supplement on PDL coated wells. NPCs were differentiated into oligodendrocytes by culturing in 1x Neurobasal media, 2% B-27 serum free supplement, 2mM GlutaMAX-I supplement and 30 ng/ml of T3 on PDL coated wells. NPCs were differentiated into astrocytes by culturing in 1x DMEM, 1% N-2 supplement, 2mM GlutaMAX-I supplement and 1% FBS on Matrigel coated wells. Successful differentiation into the 3 lineages was assessed by ICC to confirm cell identity: TuJ1, Tau, MAP2 (neurons), GFAP (astrocytes), Olig2 and O4 (oligodendrocytes). After culture, cells were fixed and underwent ICC experiments for assessment of appropriate differentiation markers and absence of pluripotent markers (Please see below for ICC protocols).

NPC mRNA QuantiPlex expression analysis.
RNA was extracted from multiple clones for I-ASD, 16pDel, and control NPCs using AMRESCO's RiboZol™ RNA Extraction Reagent using the protocol for adherent cells. Total RNA was quantified and diluted to 250 ng using RNase-free water. A QuantiGene Plex Gene Expression Assay was designed and ordered through Invitrogen with the capability to assay up to 50 mRNAs at the same time for multi-plexing and high throughput capability. The panel includes 24 genes: 3 standards for normalizing mRNA expression (Ubc, B2m, Hprt) and 21 genes involved in NPC neurogenesis (Ncam1 (Neural Cell Adhesion Molecule 1), Acvr1 (Activin A Receptor Type 1), Pax6 (Paired Box 6), Zic1 (Zic Family Member 1), Id2 (Inhibitor Of DNA Binding 2), Slc1a3 (Solute Carrier Family 1 Member 3), Pten (Phosphatase And Tensin Homolog), Gata2 (GATA Binding Protein 2), Msi1 (Musashi RNA Binding Protein 1), Metrnl (Meteorin Like, Glial Cell Differentiation Regulator), NeuroD1 (Neuronal Differentiation 1), Tbx1 (T-Box Transcription Factor 1), Nes (Nestin), Metrn (Meteorin, Glial Cell Differentiation Regulator), Sox2 (SRY-Box Transcription Factor 2), Msi2 (Musashi RNA Binding Protein 2), S100b (S100 Calcium Binding Protein B), Sox1 (SRY-Box Transcription Factor 1), Eomes (Eomesodermin), Pax3 (Paired Box 3), and Inhba (Inhibin Subunit Beta A).

To quantify the mRNA, the following protocol was followed (http://assets.thermofisher.com/TFS-Assets/BID/Reference-Materials/MAN0017862_quantigene-plex-gene-expression-assay-user-guide.pdf). All samples were run in triplicate and all reagents were prepared on the day of the experiment. For each experiment the transcript count was averaged for the triplicate technical replicates, which was then normalized to the geometric mean of the 3 standard controls (Ubc, B2m, Hprt). For each run, comparative analyses of Family 1072 or 1077 I-ASD families were made to their same-sex sibling controls. The standard error of the mean (SEM) was then calculated on the normalized technical replicates for each mRNA. Biological replicates (different clones) were then averaged and SEM determined. Finally, paired Student's t-test was used to calculate significance between ASD and control NPCs.

In addition, the Coefficient of Variation (CV), a measure of assay precision, was calculated for the technical replicates of each mRNA. Briefly, CV is the average background-subtracted signal (AVG) divided by the standard deviation (SD). High CV% (over 15%) is indicative of low assay precision so these samples were removed from the final analysis. Only 5 of the 441 samples tested were removed for poor quality and high CV values. Samples within the range of 0-14% CV were included in the analysis.

Immunocytochemistry (ICC)
After 4% PFA fixation for 20 min at RT, NPCs were permeabilized with 0.3% Triton X-100 in PBS for 10 min. Then NPCs were blocked with 5% normal goat serum (NGS) for 1h before overnight incubation with primary antibodies specific to: pluripotent stem cells: Sox2 (1:1000, Abcam, ab92494); Oct4 (1:250, Santa Cruz, Sc-5279), Tra-160 (1:100 ThermoFisher), Nanog (1:100 ThermoFisher); neural precursor_markers: Nestin (1:2000–1:5000,

R&D Systems, MAB1259), Pax6 (1:300, Covance, PRB-278P), neuronal markers: β-III tubulin (TuJ1, 1:2000–1:5000, Covance, MMS-435P), Tau (1:500, Santa Cruz, Sc-5587), oligodendrocyte markers: Olig2 (1:200, Santa Cruz, sc-293163,); GalC (1:1000, Abcam, ab137750), and astrocyte markers: Glial fibrillary acidic protein (GFAP, 1:1000, Dako, G9269). Staining was visualized by using FITC- or Texas Red-conjugated fluorescent secondary antibodies (Mairet-Coello et al., 2009).

NPC culture conditions

NPC culture conditions are described in Materials and Methods and in Williams et al., 2018 but some additional experimental procedures are detailed below. 30% Expansion Media was prepared by diluting 100% Expansion Media (ThermoFisher-GIBCO: Induction of Neural Stem Cells from Human Pluripotent Stem Cells Using PSC Neural Induction Medium) by 70%, using 1:1 DMEM/F12 + Neurobasal solution (Williams et al., 2018). Basic Fibroblast Growth Factor (bFGF; FGF2; Peprotech, 100-18B) was added directly to media at plating. For coating plates, 24 well plates (Nunc, ThermoFisher, 142475) or 35 mm dishes (Corning, CLS430165) were coated with 0.1 mg/mL filter-sterilized poly-D-lysine (PDL, Sigma, P0899) for 20 min at room temperature (RT) before washing twice with dH2O for 5 min each. Then dishes were incubated overnight at RT or for 1h in 37°C incubator with 5 μg/mL laminin (LN, Invitrogen, 23017-015) diluted in 1XPBS. After incubation, dishes were washed twice with 1XPBS for 5 min each before adding appropriate media without or with growth factors. All plates and dishes for experiments were coated under these PDL/LN. Proliferation assays involving iPSCs used Matrigel coated plates according to manufacturer's protocol: https://www.stemcell.com/coating-plates-with-matrigel-for-pluripotent-stem-cell-culture.html https://www.stemcell.com/coating-plates-with-matrigel-for-pluripotent-stem-cell-culture.html

For each NPC assay comparison, cells derived from an individual came from at least 2 NPC inductions, and 3 experiments performed across passages 3 to 6. For each iPSC assay comparison, cells derived from 2-5 clones were tested except for the NIH controls where only one clone was available. To control for potential batch variability, ASD/control experiments were run either in parallel sister cultures or contemporaneously, employing the same reagents within the same week, allowing for comparisons of I-ASD or 16pDel to their respective controls. Additionally, comparisons of control and I-ASD or 16pDel NPCs were routinely set up on the same week, often the same day and after counting dissociated cells for subsequent assays, cells were plated at same starting concentration (100,000 cells/well in wells containing 30% Expansion media, or 30% Expansion media containing 10mg/ml bFGF).

DNA synthesis assay using tritiated [^{3}H]-thymidine incorporation

NPCs or iPSCs ($1x10^5$ cells/well and $2.5x10^4$ cells/well, respectively) from control and ASD subjects were plated in triplicate or quadruplicate into 24 well plates coated with poly-D-Lysine/laminin or Matrigel, respectively. 48h post-plating, cells were incubated with 0.5 μCi/mL of tritiated [^{3}H]-thymidine (PerkinElmer, NET027E001) for the final 2h. An automatic harvester collected cells onto glass fiber filters and tritiated [^{3}H]-thymidine incorporation was assessed using scintillation spectroscopy (Lu and DiCicco-Bloom, 1997).

In parallel cultures with the DNA synthesis assay, NPC's were routinely plated at $1x10^5$ cells/well in 24 well plates and incubated at 37C for 48 hrs. These ICC experiments were routinely conducted to confirm quality of NPC cultures based on appropriate expression of NPC markers (Please see ICC protocols above).

Enumeration of cell numbers

NPCs or iPSCs ($5x10^4$ cells/well and $2.5x10^4$ cells/well, respectively)were plated in duplicate or triplicate wells of a 24 well plate. To perform counting, cells were enzymatically dissociated with Accutase and quantified every 2 days for a 6-day period (or for iPSCs, once a day for 3 days), via hemocytometer in the presence of Trypan Blue (1:10, Sigma, 15250061) to ensure only live cells were included.

NPC S-phase entry using EdU incorporation

In parallel cultures with the DNA synthesis assay, cells were plated at $5x10^5$ cells/cm^2 in 35 mm dishes and incubated at 37C for 48 hrs. At 46 h cells were incubated with 5 mM EdU (ThermoFischer, C10337) for 2 h, dissociated using Accutase, and re-plated at $1x10^4$ cells/ cm^2 in new coated 35 mm dishes to allow for single cell analysis. 2 h post-plating, cells were fixed with 4% paraformaldehyde (PFA), assayed using an EdU Click-It reaction, and imaged using fluorescence microscopy. The mitotic index was assessed blind in 10 systematically random fields (10X), counting between 150-250 cells per dish in three dishes per group (Williams et al., 2018).

Cell death or Cleaved Caspase 3 expressing cells

$5x10^4$ cells/well were plated in triplicate in a 24 well plate and fixed with 4% PFA at 24 h. ICC for apoptotic marker, cleaved caspase-3 (CC-3, 1:5000, Cell Signaling Technology, 9661), was performed and visualized using biotinylated secondary antibody and Vectastain ABC Kit (1:100; Vector Laboratories, PK-4000). The horseradish peroxidase reaction was detected with 0.05% diaminobenzidine (DAB) and 0.02% H_2O_2. The reaction was stopped by washing 2X with 1XPBS. Cell death was assessed by systematically counting the total number of CC-3 positive cells in 3 X 0.3 cm rows per well via bright field microscopy at 32X.

Protein Collection and Western Blotting of human NPCs

P3-P8 NPCs were plated in 35 mm dishes at a density of $1X10^6$ cells/dish and incubated. At 48h, cells were washed twice with ice-cold PBS followed by addition of M-PER (ThermoFisher, 78501) lysis buffer containing 1x HALT protease inhibitor consisting of AEBSF, aprotinin, bestatin, E-64, leupeptin and pepstatin A. Lysed samples were sonicated on ice using an Ultrasonics Sonicator (Qsonica, LLC, Newtown CT) and subsequently spun down at 4° C to pellet cell debris. The supernatant was then transferred and measured for protein levels. Protein concentration was measured with the BCA-protein assay (Pierce, Rockford, IL) in a spectrophotometer (Beckman, Indianapolis, IN), and calculated with comparison to a bovine serum albumin (BSA) standard curve. Equivalent protein extracts per lane were then separated on 12% acrylamide gel and transferred to polyvinylidenediflouride (PVDF; Millipore, cat # IPVH00010) membrane using transfer apparatus. The membranes were blocked with 5% milk and incubated with primary antibody overnight at 4° C and followed by anti-mouse or anti- rabbit horseradish peroxidase (HRP)-conjugated secondary for 1h at room temperature or overnight at 4° C. Bands were revealed by addition of a chemiluminescent reagent Western lightning™ Plus-ECL (Perkin Elmer, Waltham, MA) and system (Omega) as previously described (Mairet-Coello et al., 2009). Quantification of signal was conducted with ImageJ.

Primary antibodies utilized for these studies included phospho-p44/42 map kinase (Thr202/Tyr204) (1:1000, Cell signaling, Beverly, MA; cat# 9101S), Total p44/42 map kinase (1:1000, Cell signaling, Beverly, MA; cat# 9102S), and GAPDH (1:25000, Meridian Life Science, Memphis, TN).

Supplemental References

BARTLETT, C. W., FLAX, J. F., FERMANO, Z., HARE, A., HOU, L., PETRILL, S. A., BUYSKE, S. & BRZUSTOWICZ, L. M. 2012. Gene x gene interaction in shared etiology of autism and specific language impairment. *Biol Psychiatry,* 72**,** 692-9.

BARTLETT, C. W., HOU, L., FLAX, J. F., HARE, A., CHEONG, S. Y., FERMANO, Z., ZIMMERMAN-BIER, B., CARTWRIGHT, C., AZARO, M. A., BUYSKE, S. & BRZUSTOWICZ, L. M. 2014. A genome scan for loci shared by autism spectrum disorder and language impairment. *Am J Psychiatry,* 171**,** 72-81.

LU, N. & DICICCO-BLOOM, E. 1997. Pituitary adenylate cyclase-activating polypeptide is an autocrine inhibitor of mitosis in cultured cortical precursor cells. *Proc Natl Acad Sci U S A,* 94**,** 3357-62.

MAIRET-COELLO, G., TURY, A. & DICICCO-BLOOM, E. 2009. Insulin-like growth factor-1 promotes G(1)/S cell cycle progression through bidirectional regulation of cyclins and cyclin-dependent kinase inhibitors via the phosphatidylinositol 3-kinase/Akt pathway in developing rat cerebral cortex. *J Neurosci,* 29**,** 775-88.

SEKI, T., YUASA, S. & FUKUDA, K. 2012. Generation of induced pluripotent stem cells from a small amount of human peripheral blood using a combination of activated T cells and Sendai virus. *Nat Protoc,* 7**,** 718-28.

SIMONS VIP, C. 2012. Simons Variation in Individuals Project (Simons VIP): a genetics-first approach to studying autism spectrum and related neurodevelopmental disorders. *Neuron,* 73**,** 1063-7.

WILLIAMS, M., PREM, S., ZHOU, X., MATTESON, P., YEUNG, P. L., LU, C.-W., PANG, Z., BRZUSTOWICZ, L., MILLONIG, J. H. & DICICCO-BLOOM, E. 2018. Rapid Detection of Neurodevelopmental Phenotypes in Human Neural Precursor Cells (NPCs). *JoVE,* 133**,** e56628.
