# Supplementary material for: Autism NPCs from both idiopathic and CNV 16p11.2 deletion patients exhibit dysregulation of proliferation and mitogenic responses
Source: Stem Cell Reports. 2022 May 26;17(6):1380–94. doi: 10.1016/j.stemcr.2022.04.019 (PMC9214070; doi:10.1016/j.stemcr.2022.04.019)
Supplement: Document S2. Article plus supplemental information [file mmc2.pdf]

Stem Cell Reports
Article
OPEN ACCESS

Autism NPCs from both idiopathic and CNV 16p11.2 deletion patients exhibit dysregulation of proliferation and mitogenic responses

Robert Connacher,[1,2,10] Madeline Williams,[1,2,10] Smrithi Prem,[1,2] Percy L. Yeung,[3] Paul Matteson,[4] Monal Mehta,[2,4] Anna Markov,[5] Cynthia Peng,[6] Xiaofeng Zhou,[1] Courtney R. McDermott,[1,2] Zhiping P. Pang,[1,3] Judy Flax,[7] Linda Brzustowicz,[7] Che-Wei Lu,[3,8] James H. Millonig,[1,4,*] and Emanuel DiCicco-Bloom[1,9,*]

[1]Department of Neuroscience and Cell Biology, Rutgers Robert Wood Johnson Medical School, Piscataway, NJ, USA
[2]Graduate Program in Neuroscience, Rutgers University, Piscataway, NJ, USA
[3]Child Health Institute of New Jersey, Rutgers University, New Brunswick, NJ, USA
[4]Center for Advanced Biotechnology and Medicine, Rutgers University, Piscataway, NJ, USA
[5]Department of Molecular Biology and Biochemistry, Rutgers University, Piscataway, NJ, USA
[6]Department of Cell Biology and Neuroscience, Rutgers University, Piscataway, NJ, USA
[7]Department of Genetics, Rutgers University, Piscataway, NJ, USA
[8]Department of Obstetrics, Gynecology, and Reproductive Sciences, Rutgers Robert Wood Johnson Medical School, New Brunswick, NJ, USA
[9]Department of Pediatrics, Rutgers Robert Wood Johnson Medical School, New Brunswick, NJ, USA
[10]These authors contributed equally
*Correspondence: diciccem@rwjms.rutgers.edu (E.D.-B.), millonig@cabm.rutgers.edu (J.H.M.)
https://doi.org/10.1016/j.stemcr.2022.04.019

SUMMARY

Neural precursor cell (NPC) dysfunction has been consistently implicated in autism. Induced pluripotent stem cell (iPSC)-derived NPCs from two autism groups (three idiopathic [I-ASD] and two 16p11.2 deletion [16pDel]) were used to investigate if proliferation is commonly disrupted. All five individuals display defects, with all three macrocephalic individuals (two 16pDel, one I-ASD) exhibiting hyperproliferation and the other two I-ASD subjects displaying hypoproliferation. NPCs were challenged with bFGF, and all hyperproliferative NPCs displayed blunted responses, while responses were increased in hypoproliferative cells. mRNA expression studies suggest that different pathways can result in similar proliferation phenotypes. Since 16pDel deletes *MAPK3*, P-ERK was measured. P-ERK is decreased in hyperproliferative but increased in hypoproliferative NPCs. While these P-ERK changes are not responsible for the phenotypes, P-ERK and bFGF response are inversely correlated with the defects. Finally, we analyzed iPSCs and discovered that 16pDel displays hyperproliferation, while idiopathic iPSCs were normal. These data suggest that NPC proliferation defects are common in ASD.

INTRODUCTION

Autism spectrum disorder (ASD) is a heterogeneous neurodevelopmental disorder characterized by difficulties with social interactions and communication and the presence of repetitive and restricted behaviors. Most ASD cases are idiopathic, having no known genetic cause, and only 15%–20% of ASD cases are caused by known mutations such as copy number variants (CNVs) or monogenic mutations (De La Torre-Ubieta et al., 2016). Although ASD neuropathology studies have not uncovered consistent defects, overall dysregulation of early brain development, including abnormal proliferation, has been implicated. Altered proliferation is consistent with reported changes in cortical and cerebellar neuronal numbers, macrocephaly, an imbalance in excitatory-inhibitory neurons, and focal cortical dysplasias (Amaral et al., 2008; Chomiak et al., 2013; Varghese et al., 2017). Further, several studies indicate that genetically identified autism risk genes are expressed in the midfetal cerebral cortex and specifically in radial glial cells, which are cortical neural precursor cells (NPCs; Willsey et al., 2013; Grove et al., 2019; Satterstrom et al., 2020). While cortical NPC proliferation abnormalities may contribute to ASD etiology, few studies have investigated this directly. Importantly, transcriptome studies indicate that induced pluripotent stem cell (iPSC)-derived NPCs resemble fetal forebrain, making them a relevant system to investigate this important question (Brennand et al., 2014, 2015).

Macrocephaly is observed in about 20% of idiopathic ASD cases and with certain CNVs, including 16p11.2 deletion. Structural imaging studies indicate that macrocephaly frequently results from increased brain mass, especially involving frontal and parietal lobes and the cerebellum. Individuals with 16p11.2 CNV deletion display macrocephaly ~17% of the time (Steinman et al., 2016). The CNV either deletes or duplicates 28 genes, including *MAPK3*, which encodes ERK1. Both the deletion and the duplication are correlated with increased autism risk (Devlin and Scherer, 2012; Niarchou et al., 2019) and display mirror growth phenotypes. The deletion (16pDel) often leads to macrocephaly and macrosomia, while the duplication (16pDup) exhibits microcephaly and small stature (Shinawi et al., 2010; Qureshi et al., 2014). This correlation of CNV dosage to brain size suggests not only that the genes directly influence neurogenesis, but also that "too few" or "too many" neurons

This is an open access article under the CC BY license (http://creativecommons.org/licenses/by/4.0/).

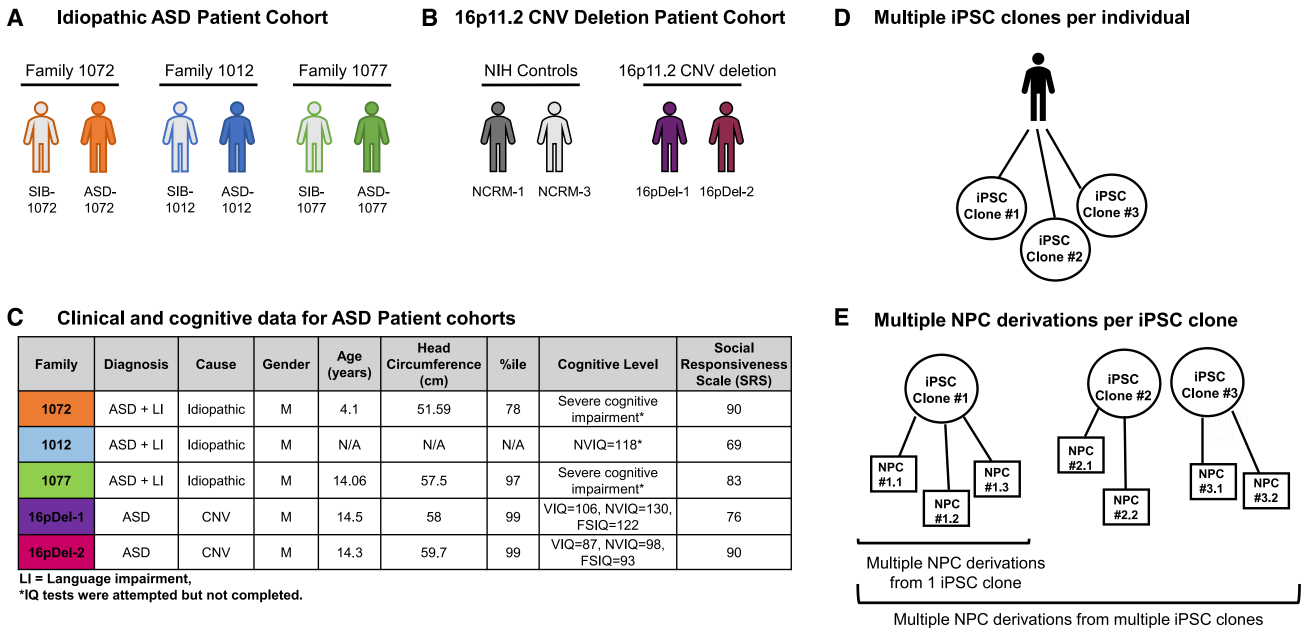

Family	Diagnosis	Cause	Gender	Age (years)	Head Circumference (cm)	%ile	Cognitive Level	Social Responsiveness Scale (SRS)
1072	ASD + LI	Idiopathic	M	4.1	51.59	78	Severe cognitive impairment*	90
1012	ASD + LI	Idiopathic	M	N/A	N/A	N/A	NVIQ=118*	69
1077	ASD + LI	Idiopathic	M	14.06	57.5	97	Severe cognitive impairment*	83
16pDel-1	ASD	CNV	M	14.5	58	99	VIQ=106, NVIQ=130, FSIQ=122	76
16pDel-2	ASD	CNV	M	14.3	59.7	99	VIQ=87, NVIQ=98, FSIQ=93	90

LI = Language impairment,
*IQ tests were attempted but not completed.

Figure 1. Patient datasets and study design

(A) For the idiopathic autism (I-ASD) dataset, iPSCs were generated from three families (family 1072, orange; 1012, blue; and 1077, green). All families had one male child diagnosed with autism and a clinically determined unaffected brother (SIB).
(B) For the 16pDel ASD dataset, iPSCs were obtained for two male individuals with autism bearing 16p11.2 deletion (purple and maroon). iPSCs from male unaffected individuals were obtained from the NIH (light gray and dark gray).
(C) Clinical data for I-ASD and 16pDel individuals are shown, including patient diagnosis, age at cell collection, Social Response Scale score (SRS), cognitive level, patient head circumference (HC), and HC percentile.
(D) The studies utilized between two and five randomly selected iPSC clones per individual.
(E) Hypothetical example demonstrating how each iPSC clone was used to derive multiple NPC lines.
LI, language impairment; N/A, not available. *See supplementary information for cognitive assessments. See Figure S1 for iPSC characterization and Figures S2 and S3 for NPC marker expression and quantification, respectively.

lead to atypical development. Previous mouse 16pDel studies do not demonstrate macrocephaly, but cortical NPCs do display hyperproliferation. This is correlated with phosphorylated ERK1 (P-ERK) signaling due to the deletion of *Mapk3* (Pucilowska et al., 2015, 2018). While iPSC models of idiopathic, macrocephalic ASD individuals have been reported, and they exhibit increased NPC proliferation (Mariani et al., 2015, Marchetto et al., 2017), these studies are not extensive and have not been extended to CNVs like 16pDel. Thus, studies utilizing human iPSC-derived NPCs from both idiopathic and genetically defined datasets like 16pDel would be ideal for studying possible proliferative phenotypes that may contribute to ASD.

In this study we investigated the proliferation of iPSC-derived NPCs from three idiopathic and two 16pDel ASD subjects and controls. Our findings indicate that proliferation is dysregulated in all five ASD individuals. We find hyperproliferation in macrocephalic individuals from both the 16pDel and the idiopathic (I-ASD) subgroups, while the other individuals exhibit hypoproliferation. Interestingly, the proliferation phenotype is inversely correlated with P-ERK levels and response to basic fibroblast growth factor (bFGF), a mitogen that stimulates the ERK pathway. In examining whether proliferation dysregulation is specific to the cells of the brain, we find that the I-ASD cohort shows no differences in their iPSCs. In contrast, iPSC proliferation in 16pDel ASD subjects parallels the increases seen in NPC proliferation. In aggregate, our findings suggest that idiopathic and 16pDel subgroups share a common phenotype of dysregulated NPC proliferation that for our datasets is inversely correlated with P-ERK levels and bFGF stimulation.

RESULTS

Study design for robust and reproducible measures of proliferation

To generate an I-ASD dataset, male autism probands and sex-matched, unaffected siblings from three families were chosen from the larger New Jersey Language and Autism Genetics Study (NJLAGS) (Figure 1A) (Bartlett et al., 2012, 2014). NJLAGS families were recruited for family members with ASD, plus a separate family member with a language

disorder called language-based learning impairment (LLI) that affects only language development. These families thus have one proband with ASD and another proband with LLI. This strategy of recruiting for two language disorders reduces phenotypic and potentially genetic heterogeneity. Importantly, all family members were phenotyped extensively and were evaluated for ASD and LLI by the same set of clinicians (see supplemental information). Thus, the unaffected sibling control for each family is diagnostically determined to not have ASD or LLI. Among the families, ASD-1072 has a normal head circumference, whereas ASD-1077 has a head circumference in the 97th percentile that is consistent with macrocephaly. No data were available for the head circumference of ASD-1012 (Figure 1C).

For the 16pDel cohort, we selected from RUCDR Infinite Biologics the only two deletion males diagnosed with autism by the Simons VIP cohort for whom iPSCs were created (see supplemental information). Both males (16pDel-1, 16pDel-2) had a head circumference at the 99th percentile (Figure 1C). For sex-matched controls, we selected two unrelated iPSC lines from the NIH Regenerative Medicine Program (NCRM-1, NCRM-3) (Figure 1B).

For each subject, multiple iPSC clones were generated. To ensure pluripotency and quality control, iPSC lines were karyotyped and assessed for expression of molecular makers of pluripotency (Figure S1). A minimum of two and up to five iPSC clones were used to induce into NPCs for each individual (Figure 1D), with the exception of NIH controls, for which only one iPSC clone was available. In addition, multiple NPC inductions were conducted for each iPSC clone (Figure 1E), especially when iPSC clone numbers were limited. To ensure quality control of NPCs, all lines were routinely immunostained for NPC markers (Nestin, SOX2, Pax6) prior to use for any experiments (see supplemental experimental procedures and Figures S2 and S3). NPC derivations were excluded from use if Nestin and/or Sox2 cell expression was <85% or Pax6 was <60%. Exact numbers of iPSC clones used to derive NPCs as well as numbers of NPC inductions per individual for all experiments are reported in Table S1.

All I-ASD individuals display dysregulated proliferation

To rigorously define proliferation in human NPCs, we employed a multi-tiered strategy consisting of simple and reproducible assays followed by more in-depth analyses to further characterize neurogenesis. We examined multiple measures of cell proliferation and cell death: total DNA synthesis by [^{3}H]thymidine labeling, S-phase labeling index, cell enumeration assays, and apoptotic marker cleaved caspase-3 immunocytochemisty (ICC). Using this strategy, we found that all I-ASD subjects display NPC proliferative differences compared with unaffected sibling controls. I-ASD individuals from two different families (families 1072 and 1012) exhibited reductions in NPC proliferation, whereas in the third macrocephalic ASD subject (family 1077), NPCs exhibited an increase in proliferation.

In the first I-ASD family examined, family 1072, we conducted numerous blinded experiments (31 for the sibling control and 36 for the ASD individual), comparing multiple separate derivations of NPCs derived from four sibling iPSC clones with those from five ASD iPSC clones. The ASD NPCs exhibited a significant 65% reduction in DNA synthesis at 48 h by ^{3}H labeling (Figure 2A). To see if changes in DNA synthesis had an impact on cell production, cell numbers were enumerated and family 1072 ASD NPCs displayed 62% and 65% reductions at 4 and 6 days, respectively (Figure 2B). In examining mechanisms underlying reduced cell proliferation, decreases in total DNA synthesis were paralleled by a 40% reduction in cells entering S phase at 48 h (Figure 2C), and ASD NPCs displayed a 55% increase in number of cells expressing cleaved caspase-3 (CC3)$^+$ at 24 h (Figure 2D). These data suggest that both a smaller proliferative population and an increase in cell death contribute to the NPC proliferation deficits in family 1072.

Similar to family 1072, ASD-1012 NPCs also exhibited a decrease in DNA synthesis, by 20% at 48 h (Figure 2E), and a 40% decrease in cell numbers at 6 days (Figure 2F). However, there was no difference in the proportion of cells entering S phase (Figure 2G), but ASD-1012 NPCs displayed a 22% increase in cell death (Figure 2H), suggesting potentially different mechanisms for the reduced cell numbers in families 1012 and 1072.

In marked contrast to these two hypoproliferative families, macrocephalic ASD-1077 NPCs displayed a 60% increase in DNA synthesis (Figure 2I) that was paralleled by a 30% increase in cell numbers at 6 days (Figure 2J). There were no differences in the proportion of cells entering S phase at 48 h (Figure 2K) nor in the proportion of cells dying at 24 h ($p = 0.0771$; Figure 2L). Since differences in cell numbers appeared only at 6 days despite increases in DNA synthesis at 48 h, these observations suggest that changes in longer-term cell survival or differentiation may play a role, which could be explored in future analyses.

In sum, all I-ASD NPCs display a proliferation phenotype (two hypoproliferative and one hyperproliferative) compared with their respective sibling. Notably, all three I-ASD individuals are not related to one another and remain genetically undefined, yet they converge on proliferative defects.

Two macrocephalic autism 16pDel individuals exhibit NPC hyperproliferation

To inquire if NPC proliferation is also altered in the 16pDel cohort, we compared NPCs from the 16pDel individuals

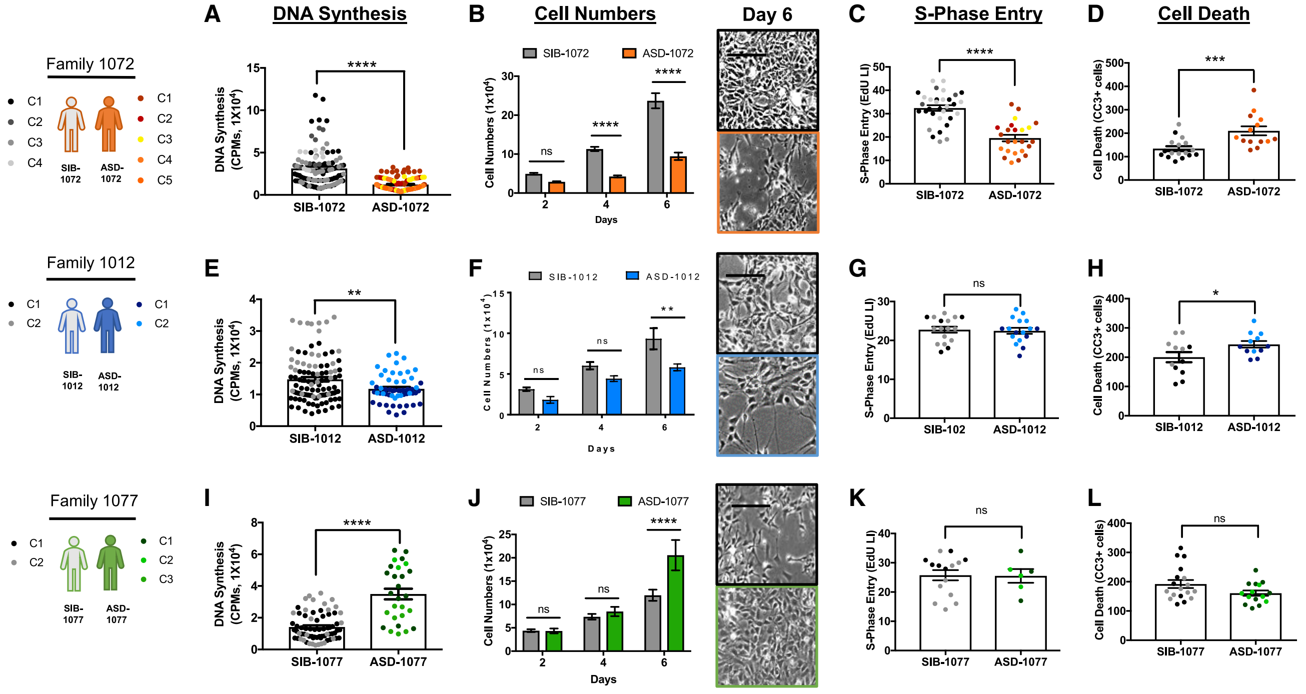

Figure 2. Idiopathic ASD NPCs from three families display dysregulated proliferation phenotypes

(A–D) At 48 h, (A) Family 1072 ASD exhibited a 65% reduction in DNA synthesis in comparison to SIB and (B) significantly reduced cell numbers at 4 and 6 days. Representative images of day 6 cultures are shown. Scale bars: 50 μm. (C) Family 1072 ASD also displayed a 40% reduction in S-phase entry (labeling index) at 48 h (see Figure S4 for images), and (D) at 24 h, a 55% increase in number of cells expressing cleaved caspase-3 (CC3). Different colored data points represent different iPSC clones used for NPC derivations.

(E–H) Family 1012 ASD exhibited (E) a 20% reduction in 48 h DNA synthesis and (F) at 6 days displayed a 40% reduction in cell numbers shown in representative images. (G) Family 1012 ASD exhibited no difference in S-phase entry at 48 h, while (H) at 24 h displayed a 22% increase in cells expressing CC3.

(I–L) Family 1077 ASD, who has macrocephaly, exhibited (I) a 60% increase in DNA synthesis and (J) a 30% increase in cell numbers at day 6, shown in representative images. (K) Family 1077 ASD displayed no change in S-phase entry at 48 h, and (L) at 24 h, no difference in numbers of cells expressing CC3. For the three sib pairs, the sample sizes were number of individuals, number of iPSC clones, number of NPC derivations, number of experiments, and number of wells, and the ranges for each sib pair were, for Family 1072 (A–D), SIB, 1, 2–4, 2–5, 6–31, and 17–105, and ASD, 1, 2–5, 3–9, 5–36, and 14–118; for Family 1012 (E–H), SIB, 1, 1–2, 1–5, 2–21, and 5–106, and ASD, 1, 1–2, 1–8, 2–14, and 6–56; and for Family 1077 (I–L), SIB, 1, 2, 2–5, 5–25, and 15–74, and ASD, 1, 2–3, 2–4, 2–9, and 6–27.

See Table S1 for iPSC and NPC sample size details. Data represent the mean ± SEM (*$p < 0.05$, **$p < 0.01$, ***$p < 0.001$, ****$p < 0.0001$).

with two sex-matched genetically typical controls. Both 16pDel individuals are macrocephalic and exhibit a significant increase in DNA synthesis at 48 h (Figure 3A). Specifically, a 90% increase was observed for 16pDel-1 and 36% increase for 16pDel-2. This 90% increase for 16pDel-1 was significantly different from that of 16pDel-2, suggesting that variability in phenotype can exist among individuals with the same CNV (Figure S5).

This increase in early DNA synthesis was paralleled by a 55% increase in cell numbers after 6 days for 16pDel-1, and 28% for 16pDel-2 (Figure 3B). Single-cell analysis indicated that both 16pDel-1 and 16pDel-2 NPCs exhibited increased S-phase entry at 48 h, with 15% and 19% increases, respectively, compared with NIH NPCs (Figure 3C). On the other hand, neither of the 16pDels' NPCs exhibited differences in $CC3^+$ cells compared with NIH controls at 24 h (Figure 3D). Our finding of increased NPC proliferation that correlates with macrocephaly supports previous reports (Mariani et al., 2015; Marchetto et al., 2017). These data also show for the first time that human NPCs from 16pDel individuals with macrocephaly display increased proliferation, evidenced by increases in DNA synthesis, cell numbers, and EdU labeling index.

Dysregulation of proliferation in autism NPCs correlates inversely with mitogenic response to basic fibroblast growth factor

Finding that both 16pDel and I-ASD cohorts exhibited altered proliferation, we tested if these phenotypes might be correlated with changes in response to mitogenic

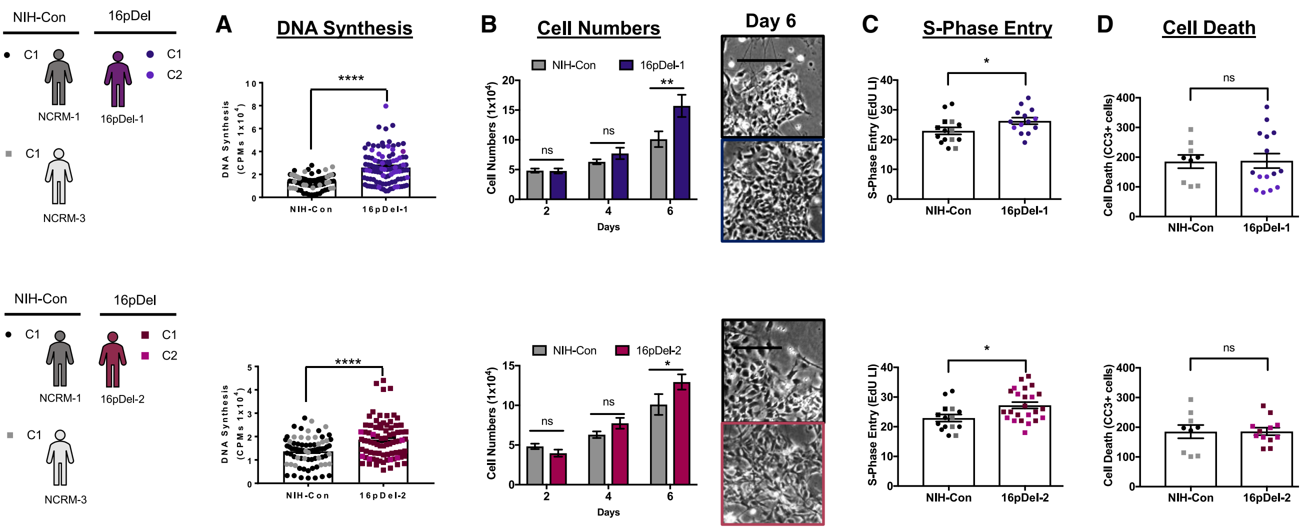

Figure 3. NPCs from two individuals with 16p11.2 CNV deletion (16pDel), autism, and macrocephaly exhibit hyperproliferation in comparison with NIH controls

(A–D) 16pDel-1 NPCs (A) exhibited a 90% increase in DNA synthesis, whereas 16pDel-2 exhibited a 36% increase in DNA synthesis compared with NIH controls at 48 h. (B) After 6 days, 16pDel-1 and 16pDel-2 exhibited 55% and 28% increases, respectively, in cell numbers. Representative images are shown. Scale bars: 50 μm. (C) 16pDel-1 and 16pDel-2 exhibited 15% and 19% increases, respectively, in 48 h S-phase entry. (D) 16pDel-1 and 16pDel-2 exhibited no differences in cells expressing CC3 at 24 h.

Data represent the mean ± SEM (*p < 0.05, **p < 0.01, ****p < 0.0001). See Figure S4 for Edu labeling and Figure S5 for NIH control and 16pDel comparisons. The sample sizes were number of individuals, number of iPSC clones, number of NPC derivations, number of experiments, and number of wells, and the ranges for each individual were 16pDel-1, 1, 2, 2–5, 4–31, and 10–108; NCRM, 2, 2, 2–9, 3–21, and 9–75. See Table S1 for iPSC and NPC sample size details.

signaling, which affects both cell proliferation and death. We challenged NPCs with a developmentally relevant mitogen, bFGF (FGF2), that is required for normal control of cortical progenitor proliferation (Wagner et al., 1999; Li and DiCicco-Bloom, 2004; Thisse and Thisse, 2005; Stevens et al., 2010). Following stimulation of NPCs for 48 h with a range of bFGF concentrations (0.03–10 ng/mL), we measured total DNA synthesis. NPC response to bFGF is expressed as a percentage of control (Figure 4). While all individuals responded to bFGF stimulation and displayed a dose-response profile, differences in response magnitude were observed (Figure 4). Notably, NPCs that shared a hyperproliferative phenotype across both datasets (ASD-1077, 16pDel-1, 16pDel-2; Figures 2 and 3) exhibited a less robust response to bFGF stimulation. Specifically, the ASD-1077 NPC response to bFGF was blunted by ~20%–30% compared with the sib control (Figure 4A). 16pDel-1 displayed an ~15%–30% reduction in response and 16pDel-2 displayed ~15%–20% diminished response to bFGF compared with NIH controls (Figures 4B and 4C).

In contrast, ASD NPCs that exhibited hypoproliferation (I-ASD-1072 and -1012) displayed an increase in DNA synthesis sensitivity and response to bFGF stimulation (Figures 4D and 4E). Both ASD-1072 and ASD-1012 displayed a 15%–20% larger response to bFGF in comparison with sibs. Together, these data indicate that NPC proliferation phenotypes are observed across our two ASD cohorts, and within our datasets, bFGF stimulation is inversely correlated with the proliferative defect. Differences in proliferation and mitogenic response may reflect alterations in the components of the FGF signaling pathway, including FGF receptors, receptor adaptor proteins, and activation of downstream kinases.

Molecular analysis of I-ASD and 16pDel NPCs

mRNA expression analysis

The above analysis suggests that there are overlapping proliferation phenotypes for 16pDel and I-ASD but also individual-specific differences, implying that underlying mechanisms may be different. To examine this further, we next measured the mRNA expression levels for a series of proliferative NPC markers. We investigated if each ASD individual had his own individualized gene expression pattern or, alternatively, if there were similarities among all ASD cases or within the subgroups (I-ASD and 16pDel). A multiplex, high-throughput Quantiplex panel was developed for 21 genes that are used as markers for human NPC proliferation. NPCs from the two I-ASD families with the greatest differences in proliferation (families 1072 and 1077) were examined. Family 1077 revealed no significant changes

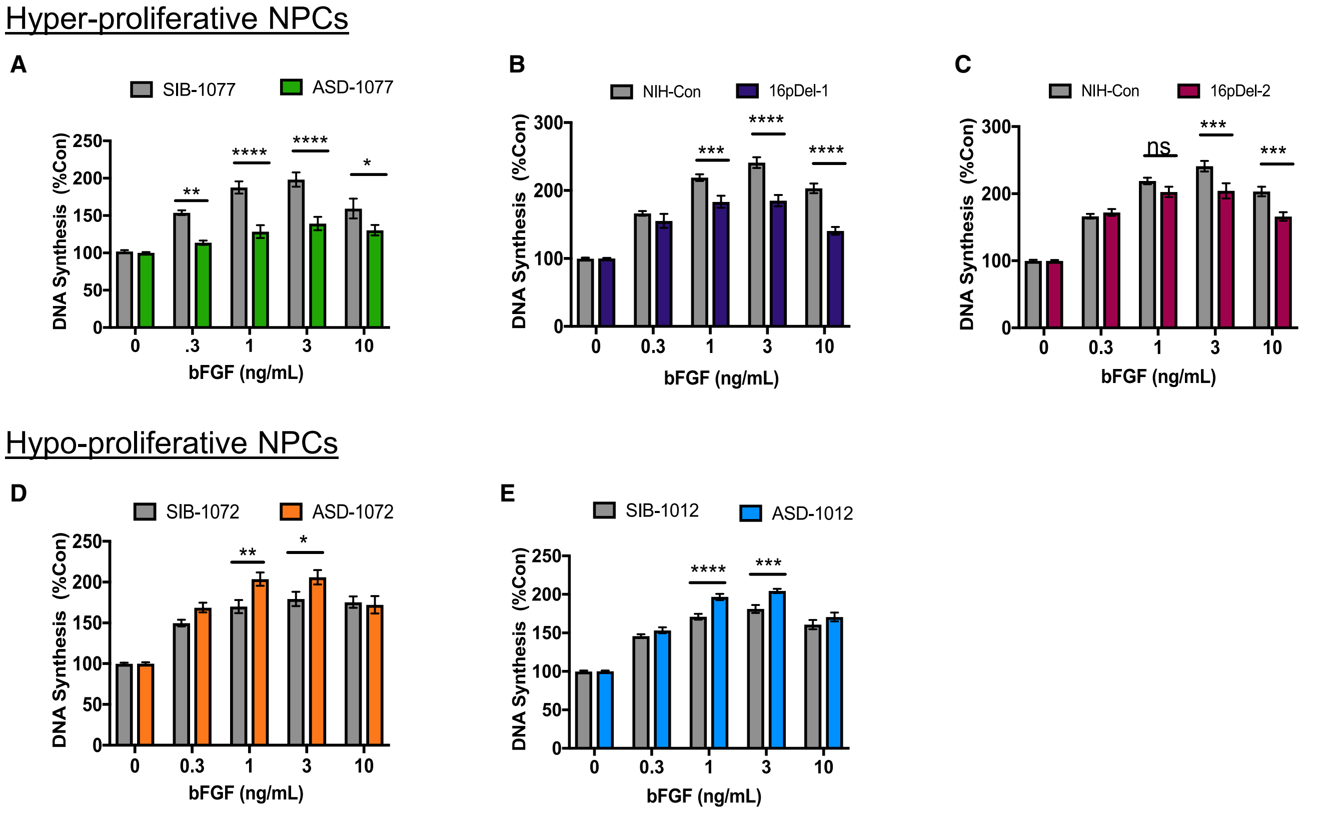

Figure 4. ASD NPCs exhibit altered mitogenic sensitivity and response to bFGF that correlates inversely with proliferation phenotypes

DNA synthesis was assessed at 48 h of culture incubation.

(A–C) Hyperproliferative NPCs exhibit reduced sensitivity and response to bFGF mitogenic stimulation. (A) Family 1077 ASD displayed ~20%–30% reduction in responses to bFGF over the 0.3 to 3 ng/mL dose range. (B) 16pDel-1 displayed 16%–30% reduction in responses to bFGF over 1 to 10 ng/mL. (C) 16pDel-2 displayed 15%–18% reduction in responses to bFGF over 3 to 10 ng/mL.

(D and E) Hypoproliferative NPCs exhibit enhanced sensitivity and response to bFGF mitogenic stimulation. (D) Family 1072 ASD displayed ~20% increase in responses to bFGF over 1 to 3 ng/mL. (E) Family 1012 ASD displayed 15% increase in responses to bFGF over 1 to 3 ng/mL bFGF. The sample sizes were number of individuals, number of iPSC clones, number of NPC derivations, number of experiments, and number of wells, and the ranges for each individual were control, 1, 1–2, 2–3, 3–6, and 9–18; and ASD, 1, 2–3, 2–4, 5–6, and 14–21.

See Table S1 for iPSC and NPC sample size details. Differences in bFGF responses did not reflect differential cell-cell contact under control or growth factor conditions; see Figure S6. Data represent the mean ± SEM (*p < 0.05, **p < 0.01, ***p < 0.001, ****p < 0.0001).

(Figure 5A), while family 1072 had only two minimally significantly differences (*ID2* and *METRN*) compared with sibling controls (Figure 5B). In contrast, the 16pDel NPCs displayed remarkable gene expression changes. Both 16pDel-1 and 16pDel-2 individuals had 12 and 15 significant differences, respectively, with eight mRNAs significantly changed in both 16pDel individuals (Figures 5C and 5D). Interestingly, 16pDel NPCs display significant mRNA reductions in both *NCAM* and *PAX6* as well as increases in *S100*. This may suggest an increased probability of glial fate. However, at 48 h, our cells immunostained only with NPC markers (Figures S2 and S3) and neither cells nor protein extracts expressed a glial marker, GFAP or S100, by ICC or western (data not shown). Thus, the marker analysis demonstrates similarities within groups but also individual differences, supporting the idea that the mechanisms underlying the common proliferation defects may vary between subgroups and individuals.

P-MAPK/ERK levels

Next, we investigated ERK levels and whether they correlated with the proliferation phenotypes. The 16p deletion includes *MAPK3*, which encodes ERK1. Given that ERK signaling affects cell growth and proliferation, we measured levels of phosphorylated and total ERK1 protein. We first examined NPCs with a hyperproliferative phenotype. As expected, NPCs from both 16pDel individuals exhibited a 50% reduction in total ERK1 protein and P-ERK1 (Figures 6A and 6A′). There was no difference in ERK2. Not surprisingly,

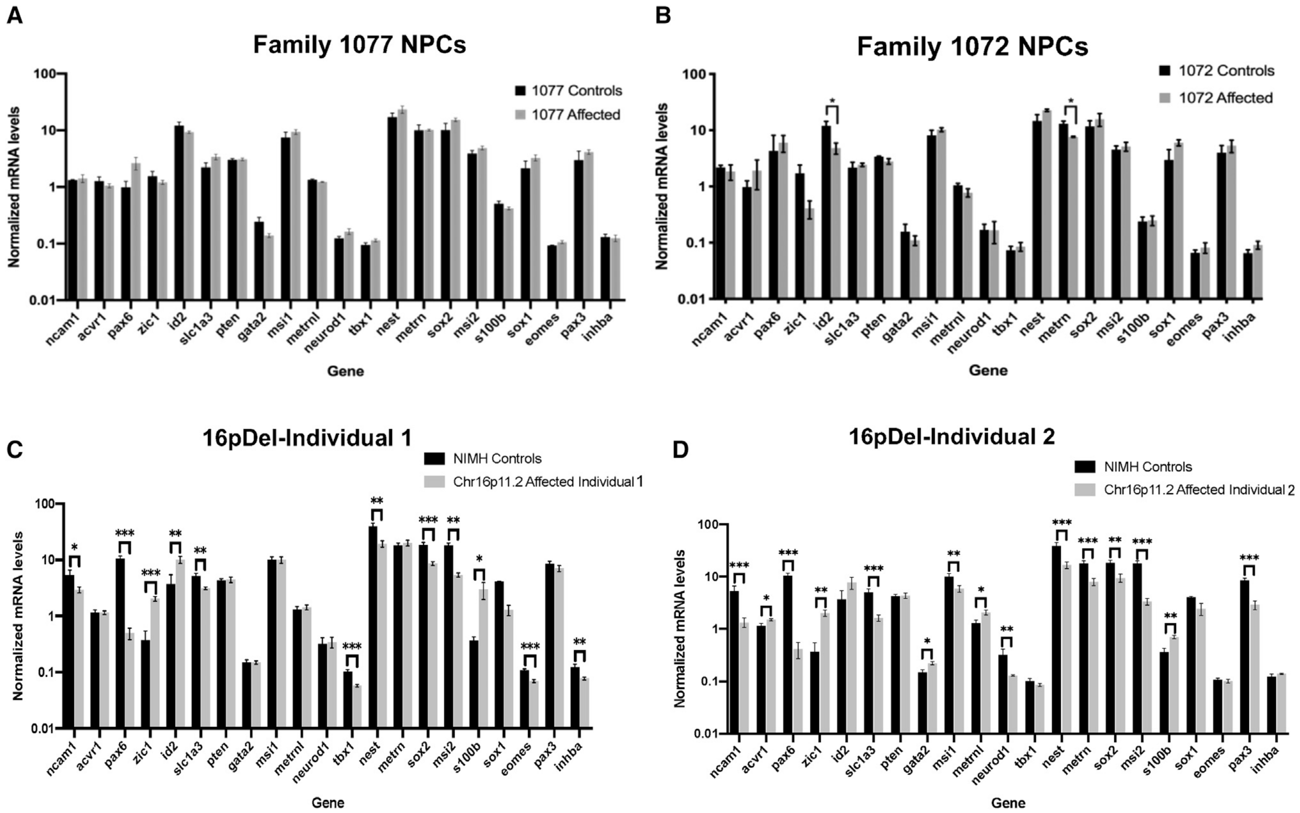

Figure 5. Gene expression of 24 NPC markers in I-ASD families and 16pDel NPCs

(A–D) NPC mRNA levels were measured using a multiplex Quantiplex panel. (A) For Family 1077, no significant differences were observed compared with the sib control, while (B) for Family 1072, two minimally significant differences were observed. For both families, three iPSC clones for both sib and I-ASD were analyzed. For 16pDel NPCs, multiple significant changes in mRNA levels were observed compared with NIH controls. (C) For 16pDel-1, two iPSC clones were analyzed and two different NIH individuals were used as controls. (D) For 16pDel-2, three iPSC clones were analyzed and two different NIH individuals were used as controls (*$p < 0.05$, **$p < 0.01$, ***$p < 0.001$; Student t test). See Table S1 for iPSC and NPC details.

when the ratio of P-ERK1 to total ERK1 was examined, it was not different between control and 16pDel NPCs. In family 1077 I-ASD, however, there were no differences in the levels of P-ERK1 (Figures 6B and 6B′), suggesting that other signaling mechanisms may contribute to this phenotype in this family. Nevertheless, for the two 16pDel individuals examined in this study, decreased ERK and P-ERK1 levels were correlated with 16pDel NPC hyperproliferation.

Given the reductions in P-ERK1 in hyperproliferative 16pDel NPCs, we wondered if there may be similar correlations in the hypoproliferative I-ASD families (ASD-1072 and -1012). Interestingly, the opposite signaling pattern was observed: a 50% increase in the levels of both total and normalized P-ERK1 (Figures 6C, 6C′, 6D, and 6D′). We further investigated the inverse correlation of the ERK pathway and proliferation by administration of small molecules. While there are no small molecules that work directly on the ERK pathway, other studies have used Fisetin as an agonist and PD98059 as an antagonist (Maher et al., 2011; Alessi et al., 1995). We titrated these small molecules on 16pDel and NIH control NPCs. No consistent change in P-ERK levels or proliferation was observed. These experiments do not support any causal effect between P-ERK levels and cell proliferation (data not shown), even though P-ERK levels are inversely correlated with the type of proliferation defect in four of the five autism NPCs across two different datasets.

Dysregulation of proliferation extends only to 16pDel iPSCs and not to I-ASD iPSCs

Finally, we assayed for proliferation phenotypes in additional cell types such as iPSCs. Interestingly, none of the three I-ASD iPSCs displayed significant changes in DNA synthesis at 48 h (Figure 7A) nor in cell numbers at 3 days (Figure 7B) compared with sibling control iPSCs. This suggests that the I-ASD dysregulation of proliferation is not global.

In contrast, proliferation of the iPSCs from the two 16pDel individuals exhibited increased DNA synthesis at

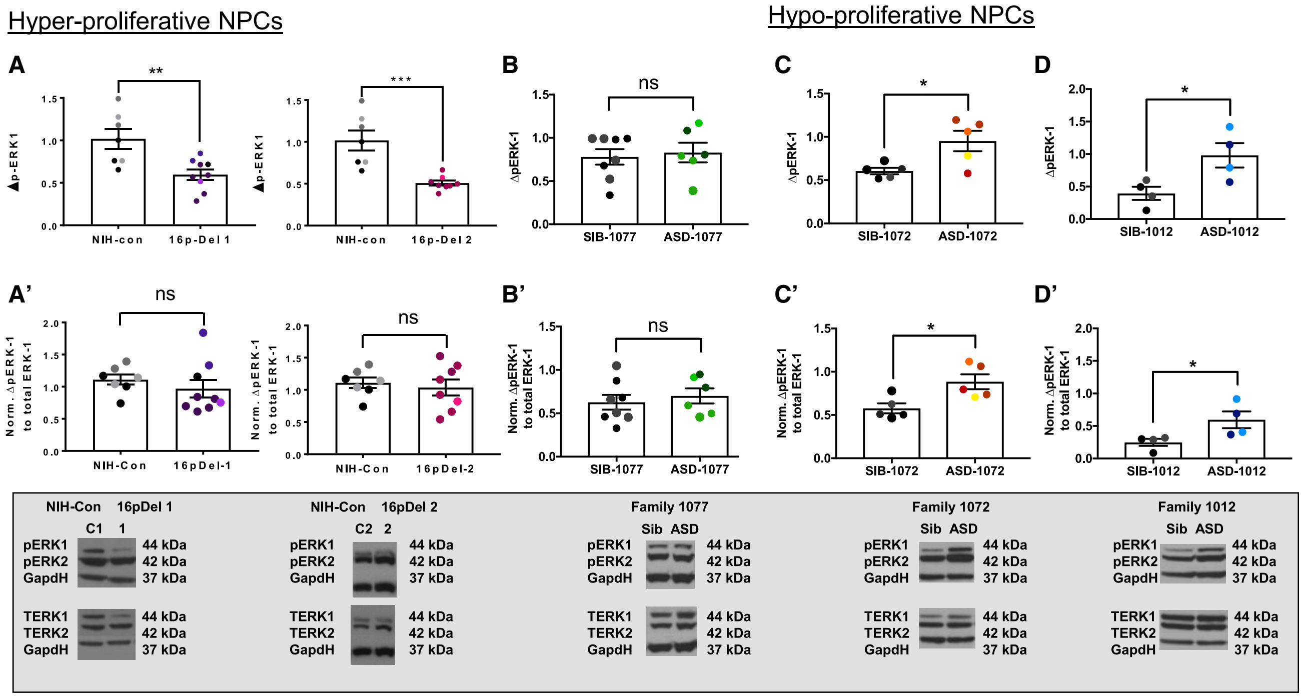

Figure 6. Absolute levels of intracellular P-ERK1 signaling inversely correlate with ASD NPC proliferation

(A and A′) Hyperproliferative 16pDel-1 and 16pDel-2 displayed ~50% reduction in P-ERK1/Gapdh levels. Normalized P-ERK1 levels for 16pDel-1 and 16pDel-2 exhibited no significant change.

(B and B′) Family 1077 exhibited unchanged P-ERK1/Gapdh and normalized P-ERK1 levels.

(C, C′, D, and D′) (C and C′) Hypoproliferative family 1072 and (D and D′) family 1012 exhibited increased P-ERK1/Gapdh and normalized P-ERK1 levels. Representative western blot images of phosphorylated ERK1/2, total ERK, and Gapdh loading control are provided for each control and ASD individual. Different colored data points represent different iPSC clones, as shown in Figures 2 and 3, and described in Figure 2 legend. Note: western blot films were cropped to align loading control and ERK bands. The sample sizes were number of individuals, number of iPSC clones, number of NPC derivations, number of experiments, and number of wells, and the ranges for each individual were control, 1–2, 2–3, 3–5, 2–4, and 4–8; and ASD, 1, 2–4, 2–4, 2–4, and 4–6.

See Table S1 for iPSC and NPC sample size details. Data represent the mean ± SEM (*p < 0.05, **p < 0.01, ***p < 0.001).

48 h, with 16pDel-1 increasing by 360% and 16pDel-2 by 148% (Figure 7C). This is similar to the 16pDel NPC results (Figure 3A). However, unlike the NPCs, we did not observe an increase in iPSC cell numbers at 3 days in culture (Figure 7D), suggesting that more complex mechanisms may be involved. These data indicate that the 16p deletion affects proliferation more broadly, unlike I-ASD, where the proliferative defects have been observed only in NPCs.

DISCUSSION

Dysregulated proliferation is a common phenotype in our sample of five ASD individuals

In this study, we employed a rigorous approach to assess neurogenesis utilizing 53 NPC derivations from 24 distinct iPSC lines of 10 individuals, to compare the proliferation phenotypes between an idiopathic ASD subgroup and the genetically defined 16pDel model. Proliferation defects were observed in all five ASD NPC lines across both subgroups, which is interesting in light of their clinical and genetic heterogeneity. We also uncovered hyperproliferation defects in macrocephalic individuals from both subgroups, with this study being the first to report proliferative defects in 16pDel NPCs. These data suggest that dysregulation of proliferation is a common defect in ASD, manifesting as either "too little" or "too much."

Our proliferation results are supported by other studies. Proliferation defects have been reported previously using iPSC-derived NPCs for both idiopathic ASD and other CNVs, such as 7q11.23 (Mariani et al., 2015; Marchetto et al., 2017; Chailangkarn et al., 2016; Li et al., 2017; Turkalj et al., 2020; Prem et al., 2020; see Connacher et al., 2018, for a review). Consistent with these data, numerous genetic studies have suggested that altered proliferation of cortical NPCs may be a potential cellular mechanism for autism risk (Krishnan et al., 2016; Packer, 2016; Grove et al., 2019; Satterstrom et al., 2020). For example, 102

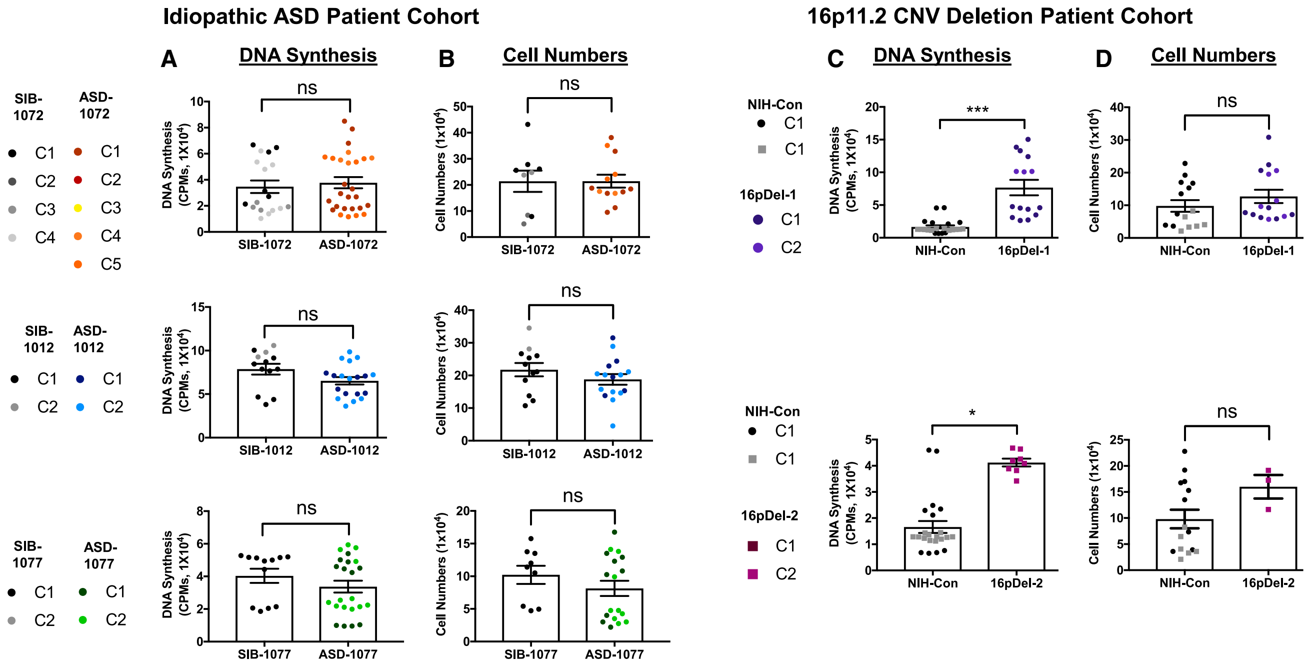

Figure 7. I-ASD iPSCs exhibit no difference in proliferation, whereas 16pDel iPSCs show an increase in DNA synthesis

(A and B) Families 1072, 1012, and 1077 I-ASD iPSCs exhibit no differences (A) in DNA synthesis at 48 h (B) or in cell numbers at day 3.

(C) 16pDel-1 iPSCs exhibited a 362% increase in DNA synthesis at 48 h, while 16pDel-2 showed a 148% increase in DNA synthesis.

(D) Neither 16pDel-1 nor 16pDel-2 iPSCs displayed a significant difference in cell numbers at day 3.

Data represent the mean ± SEM. The sample sizes were number of individuals, number of iPSC clones, number of experiments, and number of wells, and the ranges for each individual were control, 1, 1–4, 3–10, and 9–33; and ASD, 1, 1–4, 1–11, and 3–42. See Table S1 for iPSC sample size details (*$p < 0.05$, ***$p < 0.001$).

high-confidence autism risk genes have been identified, and a majority of them are expressed in embryonic forebrain NPCs at 23 weeks of gestation (Satterstrom et al., 2020). Furthermore, altered proliferation has recently been reported to be a common phenotype when the neurodevelopmental functions of multiple ASD high-confidence risk genes were examined (Willsey et al., 2021). Our study now adds further evidence that NPC proliferation dysregulation is observed in ASD across multiple forms of ASD.

While we observe consistent proliferation phenotypes, the cellular mechanisms are likely to be different between subgroups and ASD individuals. I-ASD NPCs from two families (1072, 1012) with normal head circumference exhibited significantly decreased proliferation. Family 1072 NPCs demonstrated a much greater decrease in proliferation that was paralleled by a reduction in S-phase entry and an increase in cell death, while for family 1012, a smaller decrease in proliferation correlated with a significant increase in cell death, with no difference in cells engaged in S phase. Understanding the mechanisms responsible for the decrease is an issue for future study.

Importantly, our results also add to the mounting evidence that autism NPCs from those with macrocephaly exhibit a hyperproliferation phenotype. While macrocephaly is well described in individuals who carry the 16pDel, this is the first evidence that their derived NPCs exhibit a proliferation phenotype. While both males have the same CNV deletion, they exhibit differing levels of increased proliferation, highlighting the heterogeneity of autism even within a genetically defined subgroup. Although the macrocephalic I-ASD family 1077 individual also displayed hyperproliferation, the mechanism is likely different. Indeed, compared with respective controls, family 1077 NPCs had no changes in the levels of P-ERK1 nor in NPC molecular markers, while 16pDel exhibited many mRNA differences and decreased P-ERK. Further, our findings of increased proliferation in 16pDel NPCs are in contrast to a previous study that did not find changes in proliferation or S-phase entry (Deshpande et al., 2017). While exact causes remain undefined, there are a variety of methodological differences between the studies, including iPSCs from different 16pDel individuals, NPC derivation protocols, culture duration/passage, and medium composition, that could explain the difference.

The idea that either too little or too much of a neurodevelopmental process may contribute to autism neuropathogenesis has broad support across multiple studies. For

example, both deletion and duplication lead to neurodevelopmental phenotypes of Rett syndrome and MECP2 duplication syndrome (Ramocki et al., 2009); the FXS-related gene, FMR1 (Auerbach et al., 2011; Hickey et al., 2013; Arsenault et al., 2016); and the 16p11.2 CNV (Horev et al., 2011; Niarchou et al., 2019). Further, mirror cellular and synaptic phenotypes have also been commonly reported in autism. For example, bidirectional changes in both dendritic spine densities and cell numbers in specific brain regions have been shown in multiple neuropathological studies of idiopathic autism (Amaral et al., 2008; Hutsler and Zhang, 2010; Wegiel et al., 2010, 2014; Varghese et al., 2017).

Altered mitogenic responses correlate inversely with baseline proliferation

NPCs from all idiopathic and 16pDel ASD cases displayed altered mitogenic responsiveness to bFGF, an important regulator of neurodevelopment (Tao et al., 1996; Wagner et al., 1999; Cheng et al., 2001, 2002; Li and DiCicco-Bloom, 2004; Stevens et al., 2010). Notably, the altered mitogenic response was inversely correlated with baseline NPC proliferation. In the hypoproliferative group, ASD individuals (1072, 1012) displayed an increased sensitivity and response to bFGF stimulation, whereas faster proliferators (16pDel-1 and -2, family 1077) displayed a blunted response to bFGF stimulation. This may suggest a couple of different mechanisms: (1) a ceiling effect whereby hyperproliferating NPCs are already proliferating at a high rate and cannot be further stimulated or (2) alterations in the FGF receptors or downstream signaling cascade that could either enhance or dampen pathway activation. bFGF has pleiotropic effects during development and on stem cell proliferation that can depend on growth factor concentrations (Garcia-Maya et al., 2006). One target of FGF receptor activation, membrane-linked docking/scaffolding adaptor protein FRS2α, determines the degree to which FGF activates MAP/ERK kinases and PI3K pathways. This protein is essential for FGF-induced MAP/ERK kinase stimulation and proliferation in response to low (but not high) growth factor concentrations (Hadari et al., 2001), and it regulates neurogenesis in the embryonic forebrain ventricular zone (Sato et al., 2010). These and other downstream regulators of bFGF activity will be fertile ground for future studies of mechanisms underlying NPC mitogenic responses and proliferation.

Potential role of levels of phosphorylated ERK1 in proliferative phenotypes

Alterations in signaling pathways often underpin proliferative changes in neurodevelopmental disorders. While many pathways are relevant, gene ontology studies from the Simons Foundation Autism Research Initiative (SFARI) and others have identified calcium and MAPK signaling as central nodes in ASD (Wen et al., 2016; Levitt and Campbell, 2009; Packer, 2016). Mutations in components of the MAPK signaling pathway, termed the RASopathies, produce numerous neurodevelopmental disorders, including autism (Adviento et al., 2014; Levitt and Campbell, 2009), and the *MAPK3* gene has been suggested to contribute to the head size changes in 16p11.2 individuals (Shinawi et al., 2010).

Given the proposed role of *MAPK3*/ERK1 deletion in the 16p11.2 CNV phenotype, we examined potential contributions to ASD NPC proliferation by measuring P-ERK1/2 protein levels. Our studies indicate that in four of the five ASD individuals, the absolute levels of P-ERK1 correlated inversely with the levels of NPC proliferation. In hypoproliferative I-ASD NPCs, we observed increased levels of P-ERK1 protein at baseline, a change that may underlie their increased sensitivity to bFGF stimulation. Conversely, in hyperproliferative NPCs from 16pDel individuals, we observed ~50% reduction in P-ERK1 levels, as expected given the hemizygous genetic status of CNV deletion carriers. This reduction in absolute P-ERK1 protein levels may potentially contribute to the blunted responses to bFGF in the 16pDel cohort. It is notable that, unlike the 16pDel mouse model (Pucilowska et al., 2015), we did not detect a change in the P-ERK1/total ERK1 protein ratio. Classically, the tyrosine kinase field considers intracellular signaling to depend primarily on the ratio of P-ERK protein to total ERK protein (normalized to total ERK), when describing levels of activity. However, conceptually, cellular signaling and response may also depend on the absolute amount of P-ERK1, because this may have an impact on signaling dynamics and kinetics, as well as complex interpathway interactions (Ersahin et al., 2015). Of note, additional experiments using ERK agonist Fisetin or the ERK antagonist PD98059 did not alter P-ERK levels in a predictable way and did not support the concept that alterations in P-ERK1 levels directly contribute to dysregulated proliferation in the ASD cases examined. However, this is only one of many mechanisms by which proliferation could be altered. Within 16pDel, several other genes (e.g., KCTD13, MVP) have also been suggested to play roles (Golzio et al., 2012; Escamilla et al., 2017; Arbogast et al., 2019; Kizner et al., 2020). Future studies using pharmacologic and molecular tools can investigate where the signaling dysregulation originates and the potential functional significance of P-ERK1 differences to the NPC proliferation phenotypes.

Proliferative abnormalities are differentially expressed in the iPSCs of distinct ASD subgroups

While we observed proliferative dysregulation in all NPCs, this was not the case for the iPSC clones from which the

NPCs were derived. In the three I-ASD families, iPSC proliferation was not different between ASD probands and unaffected sibling controls. In contrast, in 16pDel families, iPSC DNA synthesis was increased compared with the controls. While sample sizes were limited, these initial studies raise the possibility that molecular mechanisms that contribute to ASD may be active at different stages during development yet produce a common final phenotype. That is, in some cases, the defects may manifest within the neural lineage, such as suggested here for I-ASD, whereas in other cases, such as the 16p11.2 CNV, abnormalities may affect multiple developmental stages, cell lineages, and organ systems. Such multi-cell lineage dysfunction is also supported by clinical studies. In 16pDel individuals, ASD, epilepsy, spinal anomalies, diverse organ system congenital defects, and obesity are highly penetrant comorbid features (Shinawi et al., 2010; Steinman et al., 2016), suggesting this CNV affects multiple cell lineages and types.

Conclusions

In summary, we used a multi-tiered strategy and a rigorous and reproducible sample design to characterize neurodevelopment in ASD patient-derived cells. We identified alterations in proliferation and FGF mitogenic responses in NPCs from both idiopathic and genetic autism cohorts, suggesting that dysregulated proliferation may be a common phenotype in multiple forms of autism. Further, we identified for the first time dysregulation of proliferation in 16pDel NPCs, and provided additional evidence supporting a correlation of hyperproliferation in iPSC-derived cells with patient macrocephaly. The 16p11.2 genetic form of ASD may also have a more broadly disrupted process of proliferation, as abnormalities are observed in both iPSCs and NPCs, results that differ from those in I-ASD. Overall, these observations suggest that disruption of proliferation control during development may be one mechanism contributing to ASD pathogenesis.

EXPERIMENTAL PROCEDURES

iPSC generation and culture conditions

I-ASD iPSC lines were created by Dr. Lu as reported previously (Yi et al., 2012; Wu et al., 2012). $CD4^+$ T cells were infected and reprogrammed using standard Sendai methods (Seki et al., 2012; see supplemental information for details).

iPSC-derived neural precursor cell generation and culture conditions

NPCs were generated using neural expansion medium (Thermo Fisher, A1647801) as detailed in Williams et al. (2018) (see supplemental information for details). To verify NPC derivation, newly induced NPCs from each clone were routinely immunostained for precursor markers (Sox2, Pax6, Nestin) (see Figure S2) before use and were excluded if Nestin and/or Sox2 expression was < 85%, Pax6 was < 60%, or NPCs expressed the iPSC marker Oct4 or glial proteins (GFAP, S100b).

Proliferation assays and protein quantitation

Proliferation and apoptosis assays (^{3}H labeling, cell counting, EdU, and activated caspase-3 ICC) were performed as detailed in Williams et al. (2018). All NPC line comparisons, which used multiple iPSC clones and NPC derivations, were performed in parallel cultures or in the same week, using the same reagents and starting cell densities, to minimize technical variations (see supplemental information for details).

Western blotting was performed as described previously (Mairet-Coello et al., 2009). NPCs (1×10^6 cells/dish) from passages P3 to P8 were plated in 35 mm dishes and analyzed at 48 h (details provided in the supplemental information).

NPC mRNA Quantiplex expression analysis

RNA was isolated from I-ASD, 16pDel, and control NPCs using standard protocols, and 250 ng of total RNA was used for mRNA expression analysis. A QuantiGene Plex Assay that included 24 genes (21 genes involved in NPC proliferation and 3 controls) was designed and ordered through Invitrogen and used with standard protocols on triplicate samples (see supplemental information).

Statistics

Statistical Package GraphPad Prism version 7.0d (GraphPad Software, San Diego, CA, USA) was employed. Statistical testing involved two-tailed Student's t tests or two-way ANOVA with Sidak's multiple comparison test where specified. Data are expressed as the mean ± SEM Outliers were determined with the ROUT test and removed from analysis. In each figure legend, the number of iPSC clones used, number of total NPC derivations, number of experiments, and number of wells are detailed for each individual. For all statistical tests, sample size was the number of individual data points including all technical and biological replicates (total number of wells).

Data and code availability

Approved researchers can obtain the Simons Searchlight population dataset described in this study (https://www.sfari.org/resource/simons-searchlight/) by applying at https://base.sfari.org.

SUPPLEMENTAL INFORMATION

Supplemental information can be found online at https://doi.org/10.1016/j.stemcr.2022.04.019.

AUTHOR CONTRIBUTIONS

Conceptualization, J.H.M., E.D.-B., J.F., L.B., C.W.L., and Z.P.P.; methodology, J.H.M., P.M., P.L.Y., E.D.-B., and M.W.; formal analysis, M.W., R.C., S.P., P.M., P.L.Y., and M.M.; investigation, R.C., M.W., S.P., M.M., P.L.Y., C.W.L., A.M., C.P., X.Z., and C.R.M.; writing – original draft, M.W., R.C., M.M., J.H.M., and E.D.-B.; visualization, M.W., R.C., S.P., and M.M.; writing – review & editing, M.W., R.C., M.M., J.H.M., and E.D.-B.; funding acquisition, J.H.M. and E.D.-B.; resources, J.F., L.B., C.W.L., and P.L.Y. created

and validated all I-ASD and sib iPSC lines used in this study, and NPC lines were generated by P.L.Y., P.M., M.M., S.P., M.W., and R.C.; supervision, J.H.M., E.D.-B., P.M., and C.W.L.

CONFLICT OF INTEREST

The authors declare no competing interests.

ACKNOWLEDGMENTS

This work was supported by the New Jersey Governor's Council for Medical Research and Treatment of Autism (CAUT13APS010, CAUT14APL031, CAUT15APL041, CAUT19APL014) and the Nancy Lurie Marks Family Foundation for E.D.-B. and J.H.M.; the NJ Health Foundation (PC 63-19) for J.H.M.; the Mindworks Charitable Lead Trust and Jewish Community Foundation of Greater MetroWest for E.D.-B.; an Autism Science Foundation Undergraduate summer research grant for C.P. and E.D.-B. and Rutgers School of Graduate Studies for S.P. and E.D.-B.; NIH grants (R01 MH70366, RC1 MH088288) and the New Jersey Governor's Council for Medical Research and Treatment of Autism (CAUT12APS006, CAUT15APL026) for L.B.; a Robert Wood Johnson Foundation grant (74260) for Z.P.P.; and the Child Health Institute of New Jersey. The Brzustowicz lab expresses their sincere thanks to the NJLAGS study families, whose interest in ASD research motivated them to participate in hours of evaluation to support our research. A special thanks to the many professionals and research aides who administered and checked the testing battery. We are also grateful to all of the families at the participating Simons Searchlight sites, as well as the Simons Searchlight Consortium. We appreciate obtaining access to the phenotypic data and cellular biospecimens on SFARI Base. We thank RUCDR Infinite Biologics for providing Simons VIP 16p.11.2 deletion and NIH iPSCs, as well as technical support and guidance throughout this study.

Received: June 26, 2020
Revised: April 29, 2022
Accepted: April 30, 2022
Published: May 26, 2022

REFERENCES

Adviento, B., Corbin, I.L., Widjaja, F., Desachy, G., Enrique, N., Rosser, T., Risi, S., Marco, E.J., Hendren, R.L., Bearden, C.E., Rauen, K.A., and Weiss, L.A. (2014). Autism traits in the rasopathies. J. Med. Genet. *51*, 10–20. https://doi.org/10.1136/jmedgenet-2013-101951.

Alessi, D.R., Cuenda, A., Cohen, P., Dudley, D.T., and Saltiel, A.R. (1995). Pd 098059 is A specific inhibitor of the activation of mitogen-activated protein kinase kinase in vitro and in vivo. J. Biol. Chem. *270*, 27489–27494. https://doi.org/10.1074/jbc.270.46.27489.

Amaral, D.G., Schumann, C.M., and Nordahl, C.W. (2008). Neuroanatomy of autism. Trends Neurosci. *31*, 137–145. https://doi.org/10.1016/j.tins.2007.12.005.

Arbogast, T., Razaz, P., Ellegood, J., McKinstry, S.U., Erdin, S., Currall, B., Aneichyk, T., Lerch, J., Qiu, L.R., Rodriguiz, R.M., et al. (2019). Kctd13-Deficient mice display short-term memory impairment and sex-dependent genetic interactions. Hum. Mol. Genet. *28*, 1474–1486. https://doi.org/10.1093/hmg/ddy436.

Arsenault, J., Gholizadeh, S., Niibori, Y., Pacey, L.K., Halder, S.K., Koxhioni, E., Konno, A., Hirai, H., and Hampson, D.R. (2016). Fmrp expression levels in mouse central nervous system neurons determine behavioral phenotype. Hum. Gene Ther. *27*, 982–996. https://doi.org/10.1089/hum.2016.090.

Auerbach, B.D., Osterweil, E.K., and Bear, M.F. (2011). Mutations causing syndromic autism define an Axis of synaptic pathophysiology. Nature *480*, 63–68. https://doi.org/10.1038/nature10658.

Bartlett, C.W., Flax, J.F., Fermano, Z., Hare, A., Hou, L., Petrill, S.A., Buyske, S., and Brzustowicz, L.M. (2012). Gene × gene interaction in shared etiology of autism and specific language impairment. Biol. Psychiatry *72*, 692–699. https://doi.org/10.1016/j.biopsych.2012.05.019.

Bartlett, C.W., Hou, L., Flax, J.F., Hare, A., Cheong, S.Y., Fermano, Z., Zimmerman-Bier, B., Cartwright, C., Azaro, M.A., Buyske, S., and Brzustowicz, L.M. (2014). A genome scan for loci shared by autism spectrum disorder and language impairment. Am. J. Psychiatry *171*, 72–81. https://doi.org/10.1176/appi.ajp.2013.12081103.

Brennand, K., Savas, J.N., Kim, Y., Tran, N., Simone, A., Hashimoto-Torii, K., Beaumont, K.G., Kim, H.J., Topol, A., Ladran, I., Abdelrahim, M., Matikainen-Ankney, B., Chao, S., Mrksich, M., Rakic, P., Fang, G., Zhang, B., Yates, J.R., and Gage, F.H. (2015). Phenotypic differences in hipsc npcs derived from patients with schizophrenia. Mol. Psychiatry *20*, 361–368. https://doi.org/10.1038/mp.2014.22.

Brennand, K.J., Landek-Salgado, M.A., and Sawa, A. (2014). Modeling heterogeneous patients with a clinical diagnosis of schizophrenia with induced pluripotent stem cells. Biol. Psychiatry *75*, 936–944. https://doi.org/10.1016/j.biopsych.2013.10.025.

Chailangkarn, T., Trujillo, C.A., Freitas, B.C., Hrvoj-Mihic, B., Herai, R.H., Yu, D.X., Brown, T.T., Marchetto, M.C., Bardy, C., McHenry, L., et al. (2016). A human neurodevelopmental model for Williams Syndrome. Nature *536*, 338–343. https://doi.org/10.1038/nature19067.

Cheng, Y., Black, I.B., and Dicicco-Bloom, E. (2002). Hippocampal granule neuron production and population size are regulated by levels of Bfgf. Eur. J. Neurosci. *15*, 3–12. https://doi.org/10.1046/j.0953-816x.2001.01832.x.

Cheng, Y., Tao, Y., Black, I.B., and Dicicco-Bloom, E. (2001). A single peripheral injection of basic Fibroblast growth factor (Bfgf) stimulates granule cell production and increases cerebellar growth in newborn rats. J. Neurobiol. *46*, 220–229. https://doi.org/10.1002/1097-4695(20010215)46:3<220::aid-neu1004>3.0.co;2-p.

Chomiak, T., Turner, N., and Hu, B. (2013). What we have learned about autism Spectrum disorder from valproic acid. Patholog Res. Int. *2013*, 1–8. https://doi.org/10.1155/2013/712758.

Connacher, R.J., Dicicco-Bloom, E., and Millonig, J.H. (2018). Using human induced neural precursor cells to define early neurodevelopmental defects in syndromic and idiopathic autism. Curr. Pharmacol. Rep. *4*, 422–435. https://doi.org/10.1007/s40495-018-0155-0.

De La Torre-Ubieta, L., Won, H., Stein, J., and Geschwind, D. (2016). Advancing the understanding of autism disease

mechanisms through genetics. Nat. Med. *22*, 345–361. https://doi.org/10.1038/nm.4071.

Deshpande, A., Yadav, S., Dao, D.Q., Wu, Z.Y., Hokanson, K.C., Cahill, M.K., Wiita, A.P., Jan, Y.N., Ullian, E.M., and Weiss, L.A. (2017). Cellular phenotypes in human ipsc-derived neurons from a genetic model of autism Spectrum disorder. Cell Rep. *21*, 2678–2687. https://doi.org/10.1016/j.celrep.2017.11.037.

Devlin, B., and Scherer, S.W. (2012). Genetic architecture in autism Spectrum disorder. Curr. Opin. Genet. Dev. *22*, 229–237. https://doi.org/10.1016/j.gde.2012.03.002.

Ersahin, T., Tuncbag, N., and Cetin-Atalay, R. (2015). The pi3k/akt/mtor interactive pathway. Mol. Biosyst. *11*, 1946–1954. https://doi.org/10.1039/c5mb00101c.

Escamilla, C.O., Filonova, I., Walker, A.K., Xuan, Z.X., Holehonnur, R., Espinosa, F., Liu, S., Thyme, S.B., López-García, I.A., Mendoza, D.B., et al. (2017). Kctd13 deletion reduces synaptic transmission via increased RhoA. Nature. *551*, 227–231. https://doi.org/10.1038/nature24470.

Garcia-Maya, M., Anderson, A.A., Kendal, C.E., Kenny, A.V., Edwards-Ingram, L.C., Holladay, A., and Saffell, J.L. (2006). Ligand concentration is a driver of divergent signaling and pleiotropic cellular responses to Fgf. J. Cell Physiol. *206*, 386–393. https://doi.org/10.1002/jcp.20483.

Golzio, C., Willer, J., Talkowski, M.E., Oh, E.C., Taniguchi, Y., Jacquemont, S., Reymond, A., Sun, M., Sawa, A., Gusella, J.F., et al. (2012). Kctd13 is A major driver of mirrored neuroanatomical phenotypes of the 16p11.2 copy number variant. Nature *485*, 363–367. https://doi.org/10.1038/nature11091.

Grove, J., Ripke, S., Als, T., Mattheisen, M., Walters, R., Won, H., Pallesen, J., Agerbo, E., Andreassen, O.A., Anney, R., et al. (2019). Identification of common genetic risk variants for autism spectrum disorder. Nat. Genet. *51*, 431–444. https://doi.org/10.1038/s41588-019-0344-8.

Hadari, Y.R., Gotoh, N., Kouhara, H., Lax, I., and Schlessinger, J. (2001). Critical role for the docking-protein Frs2 alpha in Fgf receptor-mediated signal transduction pathways. Proc. Natl. Acad. Sci. U S A *98*, 8578–8583. https://doi.org/10.1073/pnas.161259898.

Hickey, S.E., Walters-Sen, L., Mosher, T.M., Pfau, R.B., Pyatt, R., Snyder, P.J., Sotos, J.F., and Prior, T.W. (2013). Duplication of the Xq27.3-Q28 region, including the Fmr1 gene, in an X-linked hypogonadism, gynecomastia, intellectual disability, short stature, and obesity syndrome. Am. J. Med. Genet. A. *161*, 2294–2299. https://doi.org/10.1002/ajmg.a.36034.

Horev, G., Ellegood, J., Lerch, J.P., Son, Y.E.E., Muthuswamy, L., Vogel, H., Krieger, A.M., Buja, A., Henkelman, R.M., and Wigler, M. (2011). Dosage-dependent phenotypes in models of 16p11.2 lesions found in autism. Proc. Natl. Acad. Sci. U S A *108*, 17076–17081. https://doi.org/10.1073/pnas.1114042108.

Hutsler, J.J., and Zhang, H. (2010). Increased dendritic spine densities on cortical projection neurons in autism spectrum disorders. Brain Res. *1309*, 83–94. https://doi.org/10.1016/j.brainres.2009.09.120.

Kizner, V., Naujock, M., Fischer, S., Jäger, S., Reich, S., Schlotthauer, I., Zuckschwerdt, K., Geiger, T., Hildebrandt, T., Lawless, N., et al. (2020). Crispr/Cas9-Mediated knockout of the neuropsychiatric risk gene Kctd13 causes developmental deficits in human cortical neurons derived from induced pluripotent stem cells. Mol. Neurobiol. *57*, 616–634. https://doi.org/10.1007/s12035-019-01727-1.

Krishnan, A., Zhang, R., Yao, V., Theesfeld, C.L., Wong, A.K., Tadych, A., Volfovsky, N., Packer, A., Lash, A., and Troyanskaya, O.G. (2016). Genome-wide prediction and functional characterization of the genetic basis of autism Spectrum disorder. Nat. Neurosci. *19*, 1454–1462. https://doi.org/10.1038/nn.4353.

Levitt, P., and Campbell, D.B. (2009). The genetic and neurobiologic compass points toward common signaling dysfunctions in autism Spectrum disorders. J. Clin. Invest. *119*, 747–754. https://doi.org/10.1172/jci37934.

Li, B., and Dicicco-Bloom, E. (2004). Basic Fibroblast growth factor exhibits dual and rapid regulation of Cyclin D_1 and $p27^{KIP1}$ to stimulate proliferation of rat cerebral cortical precursors. Dev. Neurosci. *26*, 197–207. https://doi.org/10.1159/000082137.

Li, Y., Cao, J., Chen, M., Li, J., Sun, Y., Zhang, Y., Zhu, Y., Wang, L., and Zhang, C. (2017). Abnormal neural progenitor cells differentiated from induced pluripotent stem cells partially mimicked development of Tsc2 neurological abnormalities. Stem Cell Rep. *8*, 883–893. https://doi.org/10.1016/j.stemcr.2017.02.020.

Mairet-Coello, G., Tury, A., and Dicicco-Bloom, E. (2009). Insulin-like growth factor-1 promotes G(1)/S cell cycle progression through bidirectional regulation of Cyclins and Cyclin-dependent kinase inhibitors via the phosphatidylinositol 3-kinase/akt pathway in developing rat cerebral cortex. J. Neurosci. *29*, 775–788. https://doi.org/10.1523/jneurosci.1700-08.2009.

Marchetto, M.C., Belinson, H., Tian, Y., Freitas, B.C., Fu, C., Vadodaria, K.C., Beltrao-Braga, P.C., Trujillo, C.A., Mendes, A.P.D., Padmanabhan, K., et al. (2017). Altered proliferation and networks in neural cells derived from idiopathic autistic individuals. Mol. Psychiatry *22*, 820–835. https://doi.org/10.1038/mp.2016.95.

Mariani, J., Coppola, G., Zhang, P., Abyzov, A., Provini, L., Tomasini, L., Amenduni, M., Szekely, A., Palejev, D., Wilson, M., et al. (2015). Foxg1-Dependent dysregulation of gaba/glutamate neuron differentiation in autism Spectrum disorders. Cell *162*, 375–390. https://doi.org/10.1016/j.cell.2015.06.034.

Maher, P., Dargusch, R., Bodai, L., Gerard, P.E., Purcell, J.M., and Marsh, J.L. (2011). Erk activation by the polyphenols Fisetin and resveratrol provides neuroprotection in multiple models of huntington's disease. Hum. Mol. Genet. *20*, 261–270. https://doi.org/10.1093/hmg/ddq460.

Niarchou, M., Chawner, S.J.R.A., Doherty, J.L., Maillard, A.M., Jacquemont, S., Chung, W.K., Green-Snyder, L., Bernier, R.A., Goin-Kochel, R.P., Hanson, et al. (2019). Psychiatric disorders in children with 16p11.2 deletion and duplication. Transl. Psychiatry *9*, 8. https://doi.org/10.1038/s41398-018-0339-8.

Packer, A. (2016). Neocortical neurogenesis and the etiology of autism Spectrum disorder. Neurosci. Biobehav. Rev. *64*, 185–195. https://doi.org/10.1016/j.neubiorev.2016.03.002.

Prem, S., Millonig, J.H., and Dicicco-Bloom, E. (2020). Dysregulation of neurite outgrowth and cell migration in autism and other

neurodevelopmental disorders. Adv. Neurobiol. *25*, 109–153. https://doi.org/10.1007/978-3-030-45493-7_5.

Pucilowska, J., Vithayathil, J., Pagani, M., Kelly, C., Karlo, J.C., Robol, C., Morella, I., Gozzi, A., Brambilla, R., and Landreth, G.E. (2018). Pharmacological inhibition of Erk signaling rescues pathophysiology and behavioral phenotype associated with 16p11.2 chromosomal deletion in mice. J. Neurosci. *38*, 6640–6652. https://doi.org/10.1523/jneurosci.0515-17.2018.

Pucilowska, J., Vithayathil, J., Tavares, E.J., Kelly, C., Karlo, J.C., and Landreth, G.E. (2015). The 16p11.2 deletion mouse model of autism exhibits altered cortical progenitor proliferation and brain Cytoarchitecture linked to the Erk mapk pathway. J. Neurosci. *35*, 3190–3200. https://doi.org/10.1523/jneurosci.4864-13.2015.

Qureshi, A.Y., Mueller, S., Snyder, A.Z., Mukherjee, P., Berman, J.I., Roberts, T.P.L., Nagarajan, S.S., Spiro, J.E., Chung, W.K., Sherr, E.H., and Buckner, R.L. (2014). Opposing brain differences in 16p11.2 deletion and duplication carriers. J. Neurosci. *34*, 11199–11211. https://doi.org/10.1523/jneurosci.1366-14.2014.

Ramocki, M.B., Peters, S.U., Tavyev, Y.J., Zhang, F., Carvalho, C.M.B., Schaaf, C.P., Richman, R., Fang, P., Glaze, D.G., Lupski, J.R., and Zoghbi, H.Y. (2009). Autism and other neuropsychiatric symptoms are prevalent in individuals with Mecp2 duplication Syndrome. Ann. Neurol. *66*, 771–782. https://doi.org/10.1002/ana.21715.

Sato, T., Shimazaki, T., Naka, H., Fukami, S.I., Satoh, Y., Okano, H., Lax, I., Schlessinger, J., and Gotoh, N. (2010). FRS2α regulates Erk levels to control a self-renewal target Hes1 and proliferation of FGF-responsive neural stem/progenitor cells. Stem Cells *28*, 1661–1673. https://doi.org/10.1002/stem.488.

Satterstrom, F.K., Kosmicki, J.A., Wang, J., Breen, M.S., De Rubeis, S., An, J.Y., Peng, M., Collins, R., Grove, J., Klei, L., et al. (2020). Large-scale Exome sequencing study implicates both developmental and functional changes in the neurobiology of autism. Cell *180*, 568–584. https://doi.org/10.1016/j.cell.2019.12.036.

Seki, T., Yuasa, S., and Fukuda, K. (2012). Generation of induced pluripotent stem cells from a small amount of human peripheral blood using a combination of activated T cells and Sendai virus. Nat. Protoc. *7*, 718–728. https://doi.org/10.1038/nprot.2012.015.

Shinawi, M., Liu, P., Kang, S.H.L., Shen, J., Belmont, J.W., Scott, D.A., Probst, F.J., Craigen, W.J., Graham, B.H., Pursley, A., et al. (2010). Recurrent reciprocal 16p11.2 rearrangements associated with global developmental delay, behavioural problems, dysmorphism, epilepsy, and abnormal head size. J. Med. Genet. *47*, 332–341. https://doi.org/10.1136/jmg.2009.073015.

Steinman, K.J., Spence, S.J., Ramocki, M.B., Proud, M.B., Kessler, S.K., Marco, E.J., Green Snyder, L., D'angelo, D., Chen, Q., Chung, W.K., and Sherr, E.H. (2016). 16p11.2 deletion and duplication: characterizing neurologic phenotypes in a large clinically ascertained cohort. Am. J. Med. Genet. A. *170*, 2943–2955. https://doi.org/10.1002/ajmg.a.37820.

Stevens, H.E., Smith, K.M., Maragnoli, M.E., Fagel, D., Borok, E., Shanabrough, M., Horvath, T.L., and Vaccarino, F.M. (2010). Fgfr2 is required for the development of the medial prefrontal cortex and its connections with limbic circuits. J. Neurosci. *30*, 5590–5602. https://doi.org/10.1523/jneurosci.5837-09.2010.

Tao, Y., Black, I.B., and Dicicco-Bloom, E. (1996). Neurogenesis in neonatal rat brain is regulated by peripheral injection of basic Fibroblast growth factor (bfgf). J. Comp. Neurol. *376*, 653–663. https://doi.org/10.1002/(sici)1096-9861(19961223)376:4<653::aid-cne11>3.0.co;2-n.

Thisse, B., and Thisse, C. (2005). Functions and regulations of Fibroblast growth factor signaling during embryonic development. Dev. Biol. *287*, 390–402. https://doi.org/10.1016/j.ydbio.2005.09.011.

Turkalj, L., Mehta, M., Matteson, P., Prem, S., Williams, M., Connacher, R.J., Dicicco-Bloom, E., and Millonig, J.H. (2020). Using ipsc-based models to understand the signaling and cellular phenotypes in idiopathic autism and 16p11.2 derived neurons. Adv. Neurobiol. *25*, 79–107. https://doi.org/10.1007/978-3-030-45493-7_4.

Varghese, M., Keshav, N., Jacot-Descombes, S., Warda, T., Wicinski, B., Dickstein, D.L., Harony-Nicolas, H., De Rubeis, S., Drapeau, E., Buxbaum, J.D., and Hof, P.R. (2017). Autism Spectrum disorder: neuropathology and animal models. Acta Neuropathol. *134*, 537–566. https://doi.org/10.1007/s00401-017-1736-4.

Wagner, J.P., Black, I.B., and Dicicco-Bloom, E. (1999). Stimulation of neonatal and adult brain neurogenesis by subcutaneous injection of basic fibroblast growth factor. J. Neurosci. *19*, 6006–6016. https://doi.org/10.1523/jneurosci.19-14-06006.1999.

Wegiel, J., Flory, M., Kuchna, I., Nowicki, K., Ma, S.Y., Imaki, H., Wegiel, J., Cohen, I.L., London, E., Wisniewski, T., and Brown, W.T. (2014). Stereological study of the neuronal number and volume of 38 brain subdivisions of subjects diagnosed with autism reveals significant alterations restricted to the striatum, amygdala and Cerebellum. Acta Neuropathol. Commun. *2*, 141. https://doi.org/10.1186/s40478-014-0141-7.

Wegiel, J., Kuchna, I., Nowicki, K., Imaki, H., Wegiel, J., Marchi, E., Ma, S.Y., Chauhan, A., Chauhan, V., Bobrowicz, T.W., de Leon, M., Louis, L.A.S., Cohen, I.L., London, E., Brown, W.T., and Wisniewski, T. (2010). The neuropathology of autism: defects of neurogenesis and neuronal migration, and dysplastic changes. Acta Neuropathol. *119*, 755–770. https://doi.org/10.1007/s00401-010-0655-4.

Wen, Y., Alshikho, M.J., and Herbert, M.R. (2016). Pathway network analyses for autism reveal multisystem involvement, major overlaps with other diseases and convergence upon mapk and calcium signaling. PLoS One *11*, E0153329. https://doi.org/10.1371/journal.pone.0153329.

Williams, M., Prem, S., Zhou, X., Matteson, P., Yeung, P.L., Lu, C.-W., Pang, Z., Brzustowicz, L., Millonig, J.H., and Dicicco-Bloom, E. (2018). Rapid detection of neurodevelopmental phenotypes in human neural precursor cells. Jove *133*, E56628.

Willsey, A.J., Sanders, S.J., Li, M., Dong, S., Tebbenkamp, A.T., Muhle, R.A., Reilly, S.K., Lin, L., Fertuzinhos, S., Miller, J., et al. (2013). Coexpression networks implicate human midfetal deep cortical projection neurons in the pathogenesis of autism. Cell *155*, 997–1007. https://doi.org/10.1016/j.cell.2013.10.020.

Willsey, H., Crt, E., Xu, Y., Everitt, A., Sun, N., Wang, B., Dea, J., Schmunk, G., Zaltsman, Y., Teerikorpi, N., et al. (2021). Parallel in vivo analysis of large-effect autism genes implicates cortical neurogenesis and Estrogen in risk and resilience. Neuron *109*, 1409–1804. https://doi.org/10.1016/j.neuron.2021.03.030.

Wu, D.T., Seita, Y., Zhang, X., Lu, C.W., and Roth, M.J. (2012). Antibody-directed lentiviral gene transduction for live-cell monitoring and selection of human ips and hes cells. PLoS One *7*, E34778. https://doi.org/10.1371/journal.pone.0034778.

Yi, L., Lu, C., Hu, W., Sun, Y., and Levine, A.J. (2012). Multiple roles of P53-related pathways in somatic cell reprogramming and stem cell differentiation. Cancer Res. *72*, 5635–5645. https://doi.org/10.1158/0008-5472.can-12-1451.

Stem Cell Reports, Volume *17*

Supplemental Information

Autism NPCs from both idiopathic and CNV 16p11.2 deletion patients exhibit dysregulation of proliferation and mitogenic responses

Robert Connacher, Madeline Williams, Smrithi Prem, Percy L. Yeung, Paul Matteson, Monal Mehta, Anna Markov, Cynthia Peng, Xiaofeng Zhou, Courtney R. McDermott, Zhiping P. Pang, Judy Flax, Linda Brzustowicz, Che-Wei Lu, James H. Millonig, and Emanuel DiCicco-Bloom

SUPPLEMENTAL INFORMATION

Supplemental Figures

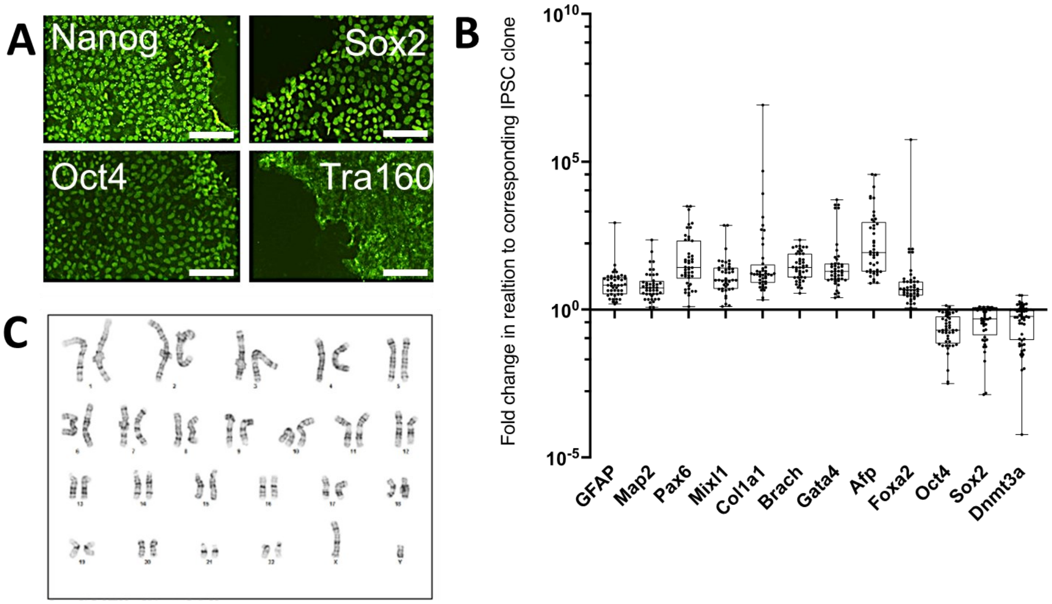

Figure S1. Characterization of iPSCs. Related to Figure 1: A) iPSCs colonies were immunostained for pluripotency markers (Tra-160, Nanog, Oct4, Sox2) as shown for a representative I-ASD individual. Scale bars: 50 µm B) Embryoid body (EB) Quanti-Plex panel demonstrates that cells express mature lineage markers for endo-, meso- and ectoderm (*GFAP*, *MAP2*, *PAX6*, *MIXL1*, *COL1A1*, *BRACH*, *GATA4*, *AFP*, *FOXA2*) and down-regulate pluripotency genes (*DNMT3A*, *OCT4*, *SOX2*). These results indicate that the iPSC clone is pluripotent. C) Representative normal karyotype of I-ASD iPSC clone.

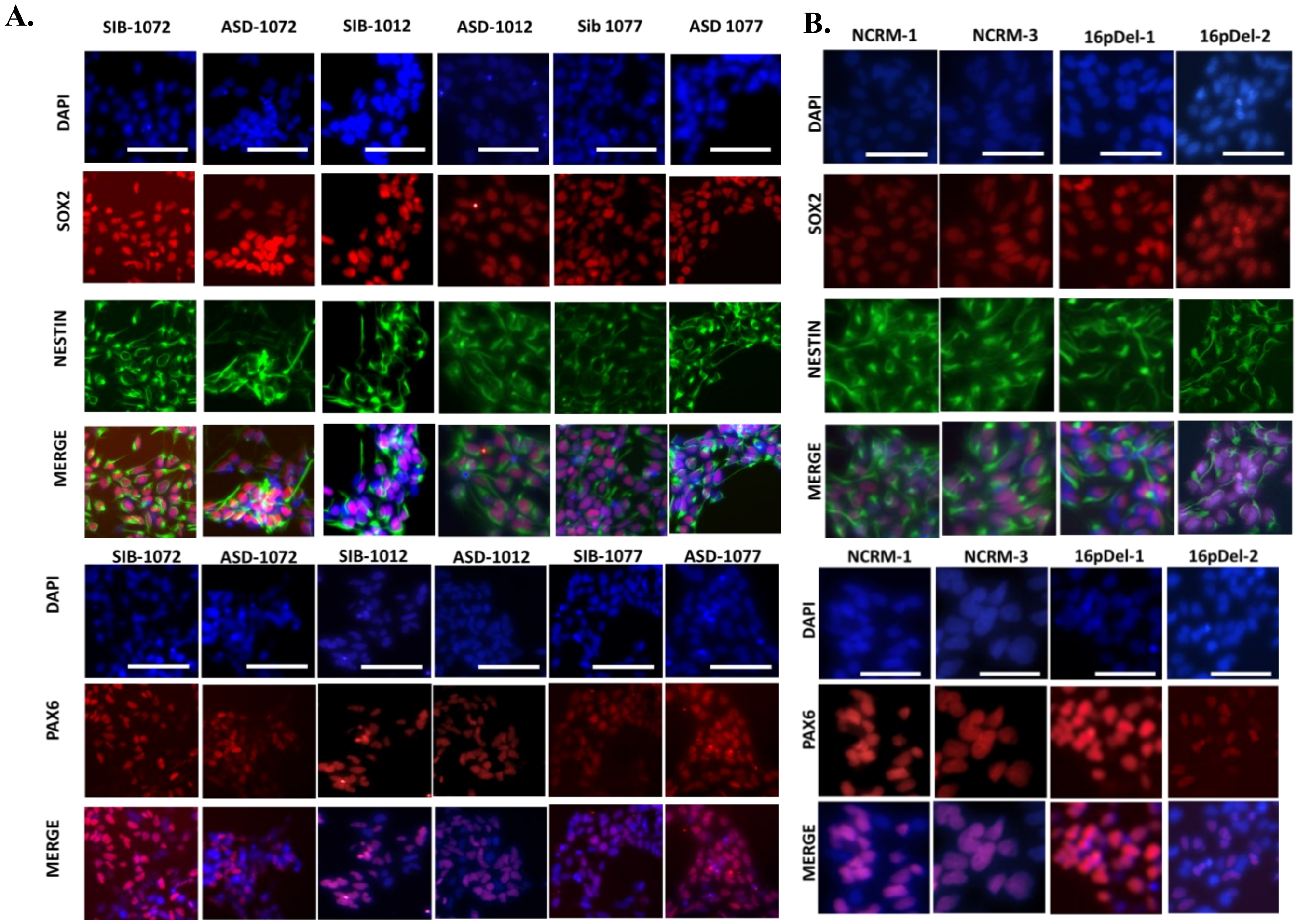

Figure S2. Representative ICC images of NPCs from control and ASD NPCs characterizing precursor marker expression. Related to Figure 1:

A and B) To ensure rigor and reproducibility, each NPC induction was routinely immunostained (100,000 NPCs per 24-well plate) with a series of NPC markers (Sox2, NESTIN, PAX6). In A, 20x magnification representative images of I-ASD affected and control sibs for families 1072, 1012, and 1077 are shown. Images for all three markers are presented along with DAPI nuclear staining and appropriate merged images. In B, identical immunostainings are shown for the 16pDel datasets (16pDel-1 and -2) and NIH controls (NCRM-1 and -3). Note: Brightness of images was increased to better visualize cells. Scale bars: 50 µm.

A.

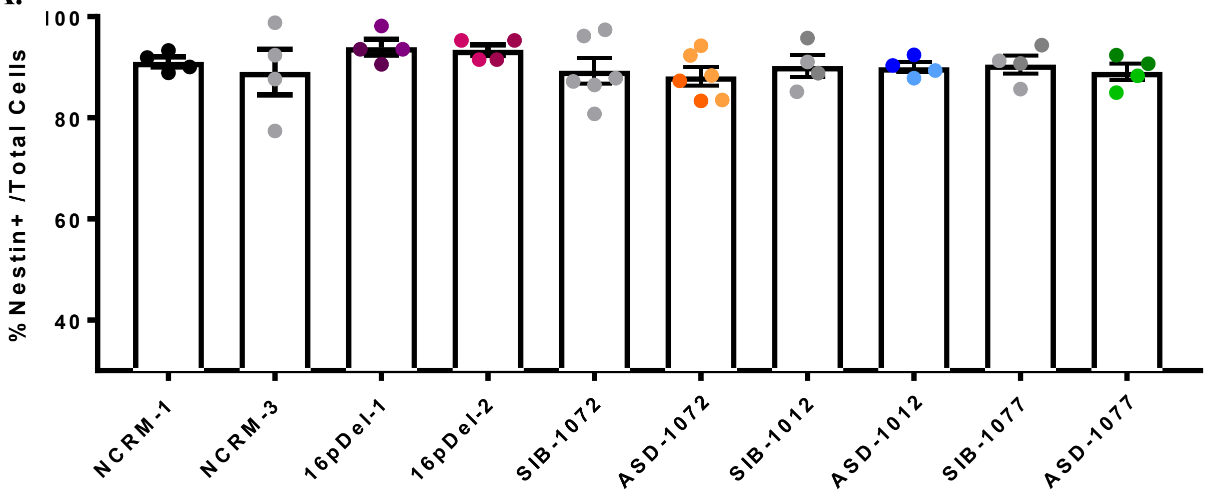

B.

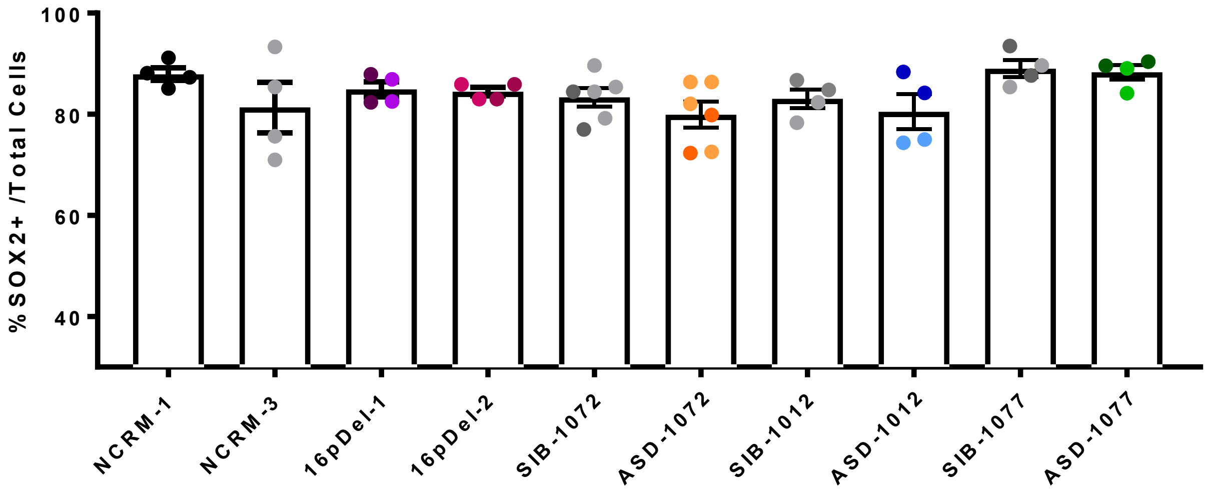

Figure S3. ICC Quantification for Nestin and SOX2 expression in control and ASD NPCs. Related to Figure 1 & Table S1:

A) Quantification of ICC images stained for Nestin. B) Quantification of ICC images stained for SOX2. All experiments were immunostained 48 hrs. Assessment of ICC stain from 35 mm dishes (50,000 cells/dish) was conducted for two clones per person and two dishes per clone for a total of 4 dishes per individual. NCRM-1 and NCRM-3 were limited to 1 clone per person and served as normal controls for 16pDel individuals. Cell counts were assessed at 20x magnification in 3 - 1cm horizontal strips across the upper, middle, and lower thirds of each dish with the 3 values analyzed as mean +/- SEM. See **Table S1** for iPSC and NPC sample size details.

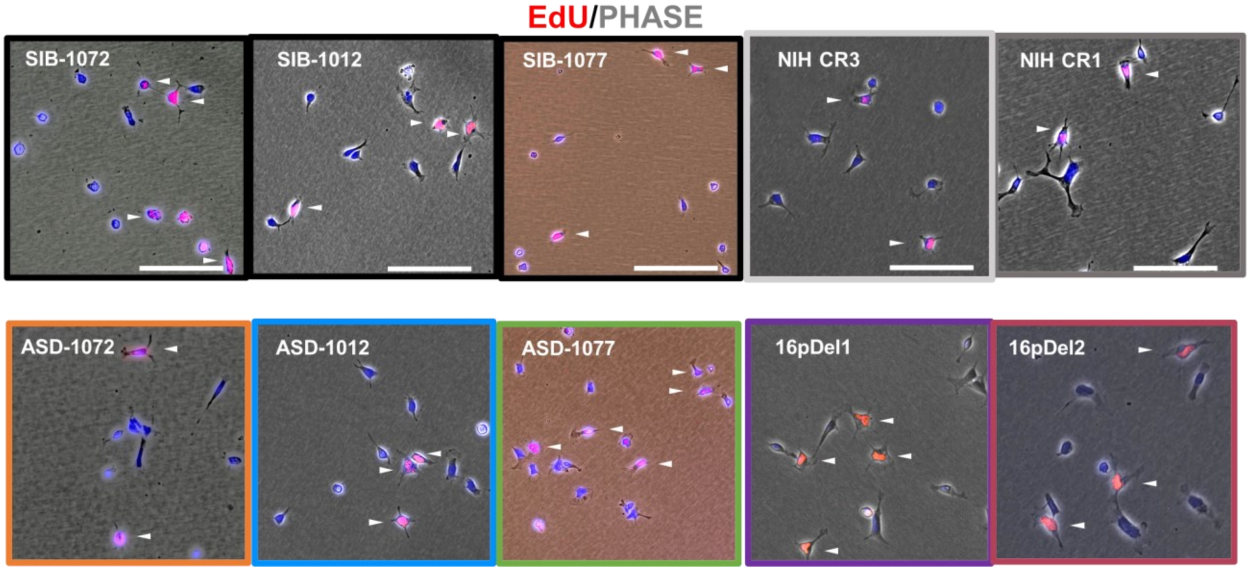

<u>Figure S4. Representative phase/ EdU images from control and ASD NPCs. Related to Figures 2, 3 & Table S1.</u>

Images of merged Phase/EdU labeling for Sib control, I-ASD, NIH control, and I6pDel NPCs demonstrating altered S phase labeling for I-ASD and 16pDel NPCs (bottom row) compared to controls (top row)(I-ASD: Families 1072, 1012 and 1077; 16pDel: 16pDel-1 &-2) in 35 mm plates (100,000 cells/ dish) at 48 hrs. White arrowheads denote viable EdU (Red) positive NPCs in each image, counterstained with DAPI (Blue). Note: Brightness of images was increased to better visualize cells. Scale bars: 50 µm. Please refer to Figure 2 C, G, K and Figure 3C for corresponding quantification of EdU experiments. See **Table S1** for iPSC and NPC sample size details.

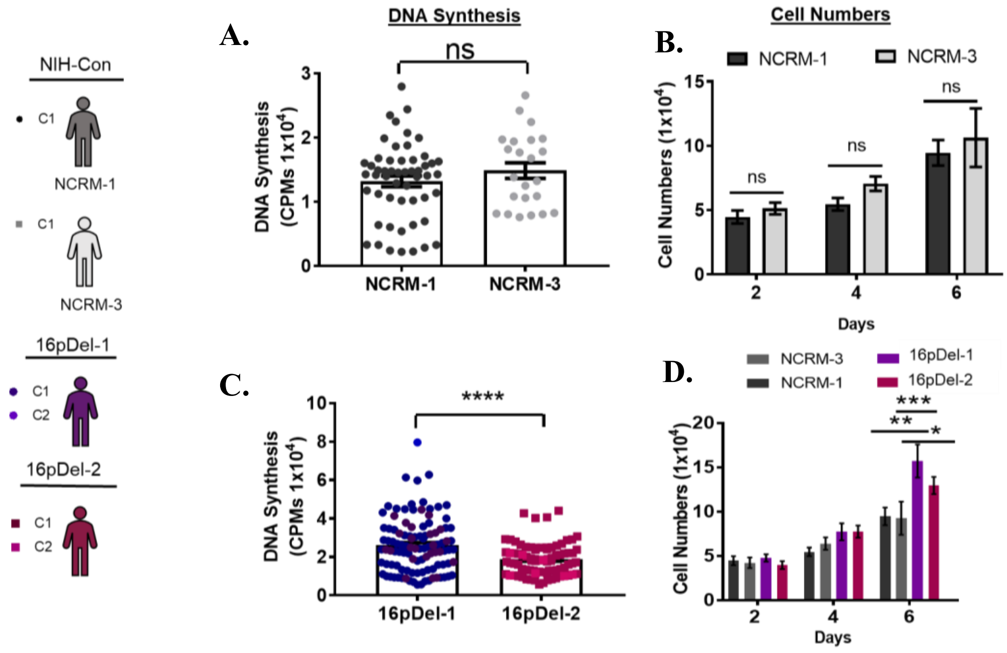

Figure S5. No differences in proliferation observed between NPCs from two NIH controls yet patient specific differences are observed in 16p11.2 CNV deletion NPCs. Related to Figure 3 and Table S1:

A) NPCs derived from NIH controls lines (NCRM-1 and NCRM-3) display non-significant differences in DNA synthesis and B) Enumeration of cell numbers at 48h. C) 16pDel-1 and 16pDel-2 NPCs exhibit a significant difference at 48h in DNA synthesis under untreated culture conditions D) Enumeration of cell numbers at 48h reveal significant increases of 16pDel NPCs to individual controls and between 16pDel-1 and 16PDel-2 by 6 days. See **Table S1** for iPSC and NPC sample size details.

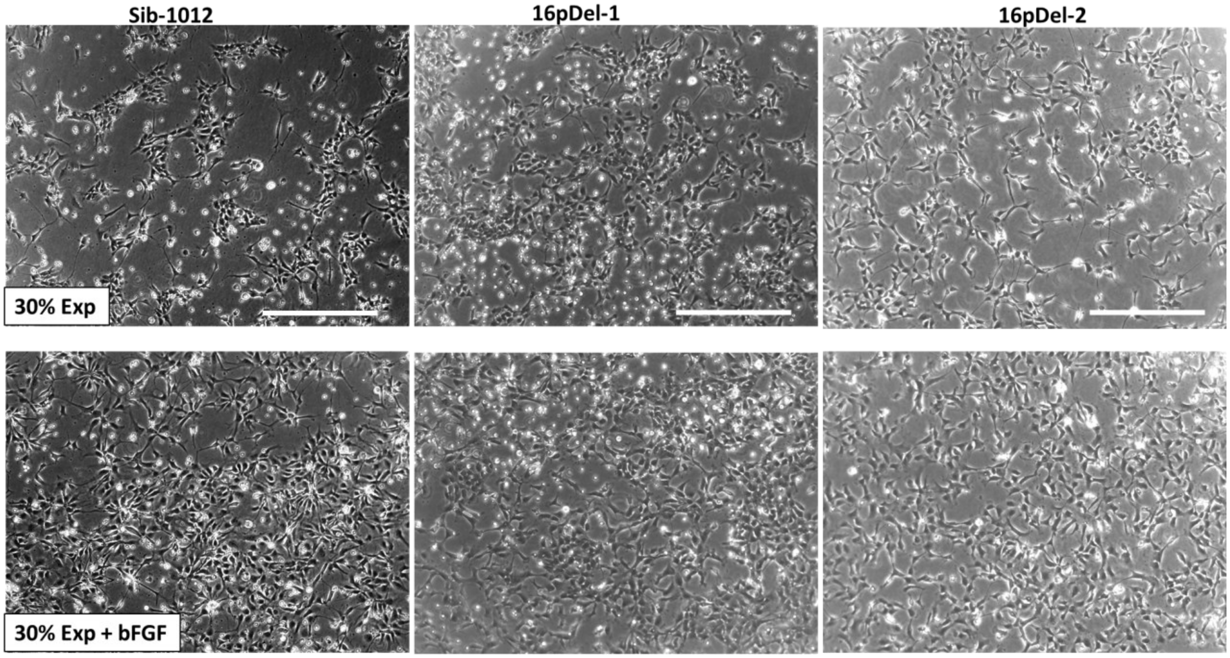

Figure S6. Representative phase images from control and ASD NPCs demonstrating that the cells do not reach confluency in the absence or presence of bFGF prior to 3H incorporation analysis. Related to Figure 4.

Phase images of Sib and 16pDel NPCs grown in 24 well plates are shown at 48hrs, demonstrating NPC density immediately prior to harvesting to assess 3H incorporation levels. Representative phase images of Sib-1012, 16pDel-1 and 16pDel-2 NPCs demonstrate that NPCs did not reach confluency +/- bFGF in 30% Expansion media. Scale bars: 250 µm

Supplemental Table 1:

Figure	Subfigure		
	**A**	**B**	**C**
**2**	SIB-1072<br>n=1/4/5/31/105	SIB-1072<br>n=1/4/5/18/39	SIB-1072<br>n=1/4/5/11/32
	ASD-1072<br>n=1/5/9/36/118	ASD-1072<br>n=1/5/9/24/51	ASD-1072<br>n=1/4/5/9/24
	**D**	**E**	**F**
	SIB-1072<br>n=1/2/2/6/17	SIB-1012<br>n=1/2/5/21/106	SIB-1012<br>n=1/2/5/9/18
	ASD-1072<br>n=1/2/3/5/14	ASD-1012<br>n=1/2/8/14/56	ASD-1012<br>n=1/2/6/7/14
	**G**	**H**	**I**
	SIB-1012<br>n=1/1/1/2/5	SIB-1012<br>n=1/2/3/4/12	SIB-1077<br>n=1/2/5/25/74
	ASD-1012<br>n=1/1/1/2/6	ASD-1012<br>n=1/2/3/4/12	ASD-1077<br>n=1/3/4/9/27
	**J**	**K**	**L**
	SIB-1077<br>n=1/2/2/12/24	SIB-1077<br>n=1/2/2/5/15	SIB-1077<br>n=1/2/2/6/18
	ASD-1077<br>n=1/3/4/8/16	ASD-1077<br>n=1/2/2/2/6	ASD-1077<br>n=1/2/2/5/15

Figure	Subfigure				
	**A**	**B**	**C**	**D**	**E**
**3**	NIH Con<br>n=2/2/9/21/75	NIH Con<br>n=2/2/5/8/16<br>(NCRM-1 n= 1/1/3/4/8, NCRM-3 n= 1/1/2/4/8)	NIH Con<br>n=2/2/4/5/15	NIH Con<br>n=2/2/2/3/9	
	16pDel-1<br>n=1/2/3/31/108	16pDel-1<br>n=1/2/4/7/14	16pDel-1<br>n=1/2/3/5/14	16pDel-1<br>n=1/2/2/5/15	
	16pDel-2<br>n=1/2/4/23/81	16pDel-2<br>n=1/2/3/5/10	16pDel-2<br>n=1/2/5/8/24	16pDel-2<br>n=1/2/3/4/12	
**4**	SIB-1077<br>n=1/2/3/6/16	NIH Con: NCRM-1 n= 1/1/2/3/10<br>NIH Con: NCRM-3 n=1/1/2/3/9	NIH Con: NCRM-1 n= 1/1/2/3/10<br>NIH Con: NCRM-3 n=1/1/2/3/9	SIB-1072<br>n=1/2/2/4/13	SIB-1012<br>n=1/2/2/6/18
	ASD-1077<br>n=1/2/3/5/14	16pDel-1<br>n=1/2/3/6/23	16pDel-2<br>n=1/2/3/6/23	ASD-1072<br>n=1/3/4/5/14	ASD-1012<br>n=1/2/2/5/21
**5**	SIB-1072: 3 iPSC clones	SIB-1072: 3 iPSC clones	NIH: 2 individuals	NIH: 2 individuals	
	ASD-1072: 3 iPSC clones	ASD-1072: 3 iPSC clones	16pDel-1: 2 iPSC clones	16pDel-2: 3 iPSC clones	
**6**	NIH Con<br>n=2/2/4/2/4	SIB-1077 n=1/2/5/4/8	SIB-1072<br>n=1/3/3/4/5	SIB-1012<br>n=1/2/3/2/4	

	16pDel-1 n=1/2/2/4/6	ASD-1077 n=1/4/4/4/6	ASD-1072 n=1/4/4/4/5	ASD-1012 n=1/2/4/2/4	
	16pDel-2 n=1/2/2/4/6				
**7**	SIB-1072 n=1/4/10/33	SIB-1072 n=1/4/11/23	NIH Con n=2/2/7/27	NIH Con n=2/2/5/15	
	SIB-1012 n=1/2/4/13	SIB-1012 n=1/2/4/12			
	SIB-1077 n=1/1/4/12	SIB-1077 n=1/1/3/9	16pDel-1 n=1/2/4/15	16pDel-1 n=1/2/5/15	
	ASD-1072 n=1/4/11/42	ASD-1072 n=1/4/10/21			
	ASD-1012 n=1/2/5/19	ASD-1012 n=1/2/5/16	16pDel-2 n=1/1/2/8	16pDel-2 n=1/1/1/3	
	ASD-1077 n=1/2/6/23	ASD-1077 n=1/2/6/18			

Table S1. Tabulation of NPC and IPSC experiments. Relating to Figures 2-7: For cells relating Fig 2-6, The n-values represent the numbers of individuals/# of iPSC clones/# of NPC derivations/# of experiments/# of wells. For cells relating to Fig 7, The n-values represent the numbers of individuals/# of iPSC clones /# of experiments/# of wells. Bolded cells indicate Figures and Subfigures.

Supplemental Experimental Procedures

Dataset information

Idiopathic Autism (I-ASD)

I-ASD iPSCs were derived from the New Jersey Language and Autism Genetics Study (NJLAGS) dataset generated by the lab of Linda Brzustowicz MD. Recruited NJLAGS families have at least one family member diagnosed with autism and at least one other family member diagnosed with Language-based Learning Impairment (LLI). Delays in other domains are not observed clinically. All family members were diagnosed by the same clinical team using the same validated clinical instruments. Autism was diagnosed by ADOS and ADI-R and DSM-IV, while LLI was diagnosed by Clinical Evaluation of Language Fundamentals-4, Comprehensive Test of Phonological Processing, Gray Oral Reading Tests-4, Woodcock Reading Mastery Tests-Revised and Weschler Abbreviated Scale of Intelligence or the Developmental Abilities Scale (Bartlett et al., 2012, Bartlett et al., 2014). Unaffected family members were clinically evaluated but did not reach diagnostic criteria for autism or LLI.

In examination of clinical phenotypes, ASD-1072 has severe cognitive impairment; an IQ test was attempted but not completed. ADOS and ADI-R revealed language comprehension limited to a small number of single words as well as occasional echolalic/scripted speech. The Social Response Scale (SRS) score was 90 denoting a severe social impairment. ASD-1072 had a head circumference at the 78th percentile at the time of measurement (4.1 years) indicating a head size in the normal range. For Family 1012, ASD-1012 has comprehension limited to a small number of single words, a nonverbal IQ (NVIQ) of 118, and an SRS score of 69 (mild/moderate range of social impairment). No data was available for the head circumference of Family 1012 proband (Figure 1C). For Family 1077, ASD-1077 has severe cognitive impairment; an IQ test was attempted but not completed. ADOS and ADI-R revealed language comprehension limited to single words and directions that are part of his routines with almost no language production. The SRS score was 83 (severe social impairment). ASD-1077 had a head circumference of 97th percentile at the time of measurement (14.06 years), which is consistent with a diagnosis of macrocephaly. Unaffected individuals were diagnostically determined to not have any language/learning impairment or ASD phenotype.

16pDel Dataset

For the 16pDel cohort, iPSCs were available from RUCDR Infinite Biologics for two males. These two male individuals were chosen from a larger cohort of 115 deletion carrier families within the Simons Foundation Autism Research Initiative (SFARI), Simons Variation in Individual project (Simons VIP) collection (Simons Vip, 2012), now renamed as Simons Searchlight. ASD inclusion criteria for 16pDel probands required they meet ADI-R and ADOS score cutoff criterion for autism spectrum disorder or autism (some individuals were clinically assessed using DSM-IV criteria). The probands were assessed on verbal and nonverbal cognitive abilities as described previously (Simons Vip, 2012). The first male (16pDel-1) exhibited Autism Spectrum Disorder (Asperger's Disorder), with a full scale IQ (FSIQ) of 122, (non-verbal IQ [NVIQ] 130, verbal IQ [VIQ] 106), head circumference at the 99th percentile at 14.5 years, consistent with macrocephaly, and comorbid expressive language disorder, anxiety, and microphthalmia with a SRS score of 76. The second male (16pDel-2) exhibited autism, with FSIQ score of 93 (NVIQ 98, VIQ 87) and a head circumference at the 99th percentile at age 14.3 years which is consistent with a macrocephaly diagnosis. He also displayed numerous other developmental phenotypes including coordination disability, developmental delay, cerebral palsy, ADD/ADHD, articulation disorder, and repetitive/expressive language disorder and a SRS score of 90.

Two iPSC clones per 16pDel individual were obtained from RUCDR Infinite Biologics. Given that genetically matched siblings for 16pDel individuals were not available, two sex matched research grade iPSC control stem cell lines (NCRM-1 and NCRM-3) were obtained from the NIH Regenerative Medicine Program (RMP) via RUCDR Infinite Biologics. iPSCs were generated from CD34+ umbilical cord blood cells from individuals who were karyotypically normal at birth, using episomal plasmid reprogramming methods. Only one iPSC clone was available for each NIH control, but a minimum of two NPC inductions were conducted for rigor and reproducibility. Subsequent cellular analyses for all assays revealed that NCRM-1 and NCRM-3 were not statistically different from one another (Supplemental Figure 5). Thus, all 16pDel comparison studies in this paper were made against a compilation of both NIH controls, specified as "NIH-Con".

iPSC generation and culture conditions

To generate I-ASD iPSCs, cryopreserved CPLs from the NJLAGS dataset were obtained from RUCDR. CPLs were thawed, grown in RPMI, 15% heat inactivated FBS, 1x glutamine and 1x Pen/Strep. $CD4^+$ T cells were negatively selected using Dynabead Untouched Human CD4 T cell kit (Life Technologies). The remaining $CD4^+$ T cells were then activated and expanded with CD3/CD28 Dynabeads (Life Technologies) with 250U/ml of IL2 for 2-3 days. 80,000 $CD4^+$ T cells were then infected and reprogrammed using a non-integrating Sendai virus expressing hOct4, hSox2, hKlf4, and hc-Myc (CytoTune) as specified by manufacturer's instructions (Seki et al., 2012). Infection occurred for 24 hours and then infected cells were plated onto irradiated MEFs and grown in KOSR media (DMEM/F12, Knockout serum replacement, non-essential amino acids, 1x glutamine and 20ng/ml human bFGF) for 2-4 weeks.

After 2-3 weeks iPSC colonies were readily visible for the affected and unaffected same sex sibling for the three I-ASD families (Family 1072, Family 1077, and Family 1012). Colonies were then picked and plated in 1x Geltrex (Thermofisher) coated wells, cultured in mTeSR media (Stemcell Technologies), passed two times and then cryopreserved. 5-50 clones were frozen for each of the six individuals. For subsequent phenotyping/neural differentiation, 3 clones from each individual were expanded, cells undergoing spontaneous differentiation were removed from each clone and the clones were passed for at least 10 passages (P10) to ensure removal of the non-integrating reprogramming Sendai virus.

To maintain iPSC lines, cells were cultured on Matrigel (Corning, 354277) and in mTeSR™1 media (Stem Cell Technologies, 85850) that was changed daily. After cells reached 70–90% confluence, they were treated with 0.5 mM EDTA diluted in 1XPBS for a minimum of 10 min. When iPSCs lifted, they were centrifuged at 150xg for 5 min, re-suspended in media, and plated at 250,000 cells/6 well plate. For the first 24h, cells were incubated with 10μM ROCK Inhibitor, Y27632 solution (Stem Cell Technologies, 72302).

To verify the pluripotency of the iPSC lines, we first performed live Tra-160 ICC after P5 followed by staining for additional iPSC markers (Tra-160, Sox2, Nanog, Oct4) on fixed cells after P10 (see Immunocytochemistry section). EB assays were then performed using standard procedures. Briefly, EBs were formed using aggre-well plates (Stem Cell Technology) and then transferred to untreated tissue culture wells. EBs were grown in EB formation media (Stem Cell Technology) and differentiated for 2-3 weeks. Next QuantiPlex mRNA expression analysis was performed for ectoderm, mesoderm, endoderm and iPSC markers (*GFAP*, *MAP2*, *PAX6*, *MIXL1*, *COL1A1*, *BRACH*, *GATA4*, *AFP*, *FOXA2*, *OCT4*, *SOX2*, *DNMT3A*). Clones were karyotyped or CGH arrays were performed and demonstrated no microscopic rearrangements are observed. For our studies iPSC clones only up to P20 were used.

16p11.2 iPSC lines were derived from CPLs and fibroblasts and RUCDR reprogrammed these cells using episomal (16pDel-1) or Sendai virus 2.0 methods (16pDel-2)(Simons Vip, 2012) respectively. Given that genetically matched siblings for 16pDel individuals were not available, sex matched iPSC lines were obtained from the NIH Regenerative Medicine Program.

Generation of hiPSC-derived neural precursor cells and culture maintenance
To generate NPCs from iPSCs, 2.5 – 3.0 x 10^4 cells/cm^2 iPSCs were plated in one well of a 6 well plate in mTeSR™1 media with 5 μM Y27632 solution for 24h. After 24h, media was replaced with Neural Induction Media (ThermoFisher Scientific, A1647801). Media was changed every other day for 7 days. After 7 days, cells were passaged and considered passage 0 (P0). For more information see manufacturers protocol and (Williams et al., 2018): ThermoFisher: GIBCO Induction of Neural Stem Cells from Human Pluripotent Stem Cells Using PSC Neural Induction Medium: https://tools.thermofisher.com/content/sfs/manuals/MAN0008031.pdf

In order to maintain lines, cells were cultured on Matrigel and maintained in Neural Expansion Media (ThermoFisher Scientific, A1647801). Media was changed every 48h until cells reached confluence at which time they were dissociated using Accutase (Gibco, A11105-01) at 37°C for 10 min, re-suspended with 1XPBS (3 – 5 mL), centrifuged at 300×g for 5 min, re-suspended in media, and counted before plating at a density of 1.0 - 1.5x10^5 cells/cm^2 per well of a 6 well plate. For the first 24h of passages 0 – 3, cells were incubated with 5 μM Y27632 solution.

In order to establish Quality Control (QC) of the iPSC-derived NPCs, we routinely performed immunostaining for Nestin, Pax6, and Sox2 for each induction and clone as a requirement for using them in experiments. QC was performed on passage 3 cells plated at 100,000 cells/24 well plates, as shown in Figures S2. Additional QC of NPCs

was also performed by quantifying cells at low density (50,000 cell/ 35 mm dish), as shown as S3 above (see ICC below for protocol). NPCs were discarded if marker immunostaining revealed Nestin or Sox2 <85% or Pax6 < 60%. Quantification of total cells (DAPI positive) as well as cells immunopositive for aforementioned marker(s) was assessed at 20x magnification in 3 – 1 cm horizontal strips across the upper, middle, and lower thirds of each dish/well. A mean +/- SEM was then calculated. For approved clones, continued monitoring of NPCs was performed and if changes in cellular morphology or cell growth were observed, then the ICC staining was conducted again at the later passage. Any NPCs failing the above immunostaining criterion were discarded.

In order to differentiate NPCs into neurons, oligodendrocytes, or astrocytes, NPCs were cultured according to manufacturers instructions www.thermofisher.com/us/en/home/references/protocols/neurobiology/neurobiology-protocols/differentiating-neural-stem-cells-into-neurons-and-glial-cells.html
Briefly NPCs were differentiated into neurons by culturing in 1x Neurobasal media, 2% B-27 serum free supplement and 2mM GlutaMAX-I supplement on PDL coated wells. NPCs were differentiated into oligodendrocytes by culturing in 1x Neurobasal media, 2% B-27 serum free supplement, 2mM GlutaMAX-I supplement and 30 ng/ml of T3 on PDL coated wells. NPCs were differentiated into astrocytes by culturing in 1x DMEM, 1% N-2 supplement, 2mM GlutaMAX-I supplement and 1% FBS on Matrigel coated wells. Successful differentiation into the 3 lineages was assessed by ICC to confirm cell identity: TuJ1, Tau, MAP2 (neurons), GFAP (astrocytes), Olig2 and O4 (oligodendrocytes). After culture, cells were fixed and underwent ICC experiments for assessment of appropriate differentiation markers and absence of pluripotent markers (Please see below for ICC protocols).

NPC mRNA QuantiPlex expression analysis.

RNA was extracted from multiple clones for I-ASD, 16pDel, and control NPCs using AMRESCO's RiboZol™ RNA Extraction Reagent using the protocol for adherent cells. Total RNA was quantified and diluted to 250 ng using RNase-free water. A QuantiGene Plex Gene Expression Assay was designed and ordered through Invitrogen with the capability to assay up to 50 mRNAs at the same time for multi-plexing and high throughput capability. The panel includes 24 genes: 3 standards for normalizing mRNA expression (Ubc, B2m, Hprt) and 21 genes involved in NPC neurogenesis (Ncam1 (Neural Cell Adhesion Molecule 1), Acvr1 (Activin A Receptor Type 1), Pax6 (Paired Box 6), Zic1 (Zic Family Member 1), Id2 (Inhibitor Of DNA Binding 2), Slc1a3 (Solute Carrier Family 1 Member 3), Pten (Phosphatase And Tensin Homolog), Gata2 (GATA Binding Protein 2), Msi1 (Musashi RNA Binding Protein 1), Metrnl (Meteorin Like, Glial Cell Differentiation Regulator), NeuroD1 (Neuronal Differentiation 1), Tbx1 (T-Box Transcription Factor 1), Nes (Nestin), Metrn (Meteorin, Glial Cell Differentiation Regulator), Sox2 (SRY-Box Transcription Factor 2), Msi2 (Musashi RNA Binding Protein 2), S100b (S100 Calcium Binding Protein B), Sox1 (SRY-Box Transcription Factor 1), Eomes (Eomesodermin), Pax3 (Paired Box 3), and Inhba (Inhibin Subunit Beta A).

To quantify the mRNA, the following protocol was followed (http://assets.thermofisher.com/TFS-Assets/BID/Reference-Materials/MAN0017862_quantigene-plex-gene-expression-assay-user-guide.pdf). All samples were run in triplicate and all reagents were prepared on the day of the experiment. For each experiment the transcript count was averaged for the triplicate technical replicates, which was then normalized to the geometric mean of the 3 standard controls (Ubc, B2m, Hprt). For each run, comparative analyses of Family 1072 or 1077 I-ASD families were made to their same-sex sibling controls. The standard error of the mean (SEM) was then calculated on the normalized technical replicates for each mRNA. Biological replicates (different clones) were then averaged and SEM determined. Finally, paired Student's t-test was used to calculate significance between ASD and control NPCs.

In addition, the Coefficient of Variation (CV), a measure of assay precision, was calculated for the technical replicates of each mRNA. Briefly, CV is the average background-subtracted signal (AVG) divided by the standard deviation (SD). High CV% (over 15%) is indicative of low assay precision so these samples were removed from the final analysis. Only 5 of the 441 samples tested were removed for poor quality and high CV values. Samples within the range of 0-14% CV were included in the analysis.

Immunocytochemistry (ICC)

After 4% PFA fixation for 20 min at RT, NPCs were permeabilized with 0.3% Triton X-100 in PBS for 10 min. Then NPCs were blocked with 5% normal goat serum (NGS) for 1h before overnight incubation with primary antibodies specific to: pluripotent stem cells: Sox2 (1:1000, Abcam, ab92494); Oct4 (1:250, Santa Cruz, Sc-5279), Tra-160 (1:100 ThermoFisher), Nanog (1:100 ThermoFisher); neural precursor_markers: Nestin (1:2000–1:5000,

R&D Systems, MAB1259), Pax6 (1:300, Covance, PRB-278P), neuronal markers: β-III tubulin (TuJ1, 1:2000–1:5000, Covance, MMS-435P), Tau (1:500, Santa Cruz, Sc-5587), oligodendrocyte markers: Olig2 (1:200, Santa Cruz, sc-293163,); GalC (1:1000, Abcam, ab137750), and astrocyte markers: Glial fibrillary acidic protein (GFAP, 1:1000, Dako, G9269). Staining was visualized by using FITC- or Texas Red-conjugated fluorescent secondary antibodies (Mairet-Coello et al., 2009).

NPC culture conditions

NPC culture conditions are described in Materials and Methods and in Williams et al., 2018 but some additional experimental procedures are detailed below. 30% Expansion Media was prepared by diluting 100% Expansion Media (ThermoFisher-GIBCO: Induction of Neural Stem Cells from Human Pluripotent Stem Cells Using PSC Neural Induction Medium) by 70%, using 1:1 DMEM/F12 + Neurobasal solution (Williams et al., 2018). Basic Fibroblast Growth Factor (bFGF; FGF2; Peprotech, 100-18B) was added directly to media at plating. For coating plates, 24 well plates (Nunc, ThermoFisher, 142475) or 35 mm dishes (Corning, CLS430165) were coated with 0.1 mg/mL filter-sterilized poly-D-lysine (PDL, Sigma, P0899) for 20 min at room temperature (RT) before washing twice with dH2O for 5 min each. Then dishes were incubated overnight at RT or for 1h in 37°C incubator with 5 µg/mL laminin (LN, Invitrogen, 23017-015) diluted in 1XPBS. After incubation, dishes were washed twice with 1XPBS for 5 min each before adding appropriate media without or with growth factors. All plates and dishes for experiments were coated under these PDL/LN. Proliferation assays involving iPSCs used Matrigel coated plates according to manufacturer's protocol: https://www.stemcell.com/coating-plates-with-matrigel-for-pluripotent-stem-cell-culture.html https://www.stemcell.com/coating-plates-with-matrigel-for-pluripotent-stem-cell-culture.html

For each NPC assay comparison, cells derived from an individual came from at least 2 NPC inductions, and 3 experiments performed across passages 3 to 6. For each iPSC assay comparison, cells derived from 2-5 clones were tested except for the NIH controls where only one clone was available. To control for potential batch variability, ASD/control experiments were run either in parallel sister cultures or contemporaneously, employing the same reagents within the same week, allowing for comparisons of I-ASD or 16pDel to their respective controls. Additionally, comparisons of control and I-ASD or 16pDel NPCs were routinely set up on the same week, often the same day and after counting dissociated cells for subsequent assays, cells were plated at same starting concentration (100,000 cells/well in wells containing 30% Expansion media, or 30% Expansion media containing 10mg/ml bFGF).

DNA synthesis assay using tritiated [^{3}H]-thymidine incorporation

NPCs or iPSCs ($1x10^5$ cells/well and $2.5x10^4$ cells/well, respectively) from control and ASD subjects were plated in triplicate or quadruplicate into 24 well plates coated with poly-D-Lysine/laminin or Matrigel, respectively. 48h post-plating, cells were incubated with 0.5 µCi/mL of tritiated [^{3}H]-thymidine (PerkinElmer, NET027E001) for the final 2h. An automatic harvester collected cells onto glass fiber filters and tritiated [^{3}H]-thymidine incorporation was assessed using scintillation spectroscopy (Lu and DiCicco-Bloom, 1997).

In parallel cultures with the DNA synthesis assay, NPC's were routinely plated at $1x10^5$ cells/well in 24 well plates and incubated at 37C for 48 hrs. These ICC experiments were routinely conducted to confirm quality of NPC cultures based on appropriate expression of NPC markers (Please see ICC protocols above).

Enumeration of cell numbers

NPCs or iPSCs ($5x10^4$ cells/well and $2.5x10^4$ cells/well, respectively)were plated in duplicate or triplicate wells of a 24 well plate. To perform counting, cells were enzymatically dissociated with Accutase and quantified every 2 days for a 6-day period (or for iPSCs, once a day for 3 days), via hemocytometer in the presence of Trypan Blue (1:10, Sigma, 15250061) to ensure only live cells were included.

NPC S-phase entry using EdU incorporation

In parallel cultures with the DNA synthesis assay, cells were plated at $5x10^5$ cells/cm^2 in 35 mm dishes and incubated at 37C for 48 hrs. At 46 h cells were incubated with 5 mM EdU (ThermoFischer, C10337) for 2 h, dissociated using Accutase, and re-plated at $1x10^4$ cells/ cm^2 in new coated 35 mm dishes to allow for single cell analysis. 2 h post-plating, cells were fixed with 4% paraformaldehyde (PFA), assayed using an EdU Click-It reaction, and imaged using fluorescence microscopy. The mitotic index was assessed blind in 10 systematically random fields (10X), counting between 150-250 cells per dish in three dishes per group (Williams et al., 2018).

Cell death or Cleaved Caspase 3 expressing cells
$5x10^4$ cells/well were plated in triplicate in a 24 well plate and fixed with 4% PFA at 24 h. ICC for apoptotic marker, cleaved caspase-3 (CC-3, 1:5000, Cell Signaling Technology, 9661), was performed and visualized using biotinylated secondary antibody and Vectastain ABC Kit (1:100; Vector Laboratories**,** PK-4000). The horseradish peroxidase reaction was detected with 0.05% diaminobenzidine (DAB) and 0.02% H_2O_2. The reaction was stopped by washing 2X with 1XPBS. Cell death was assessed by systematically counting the total number of CC-3 positive cells in 3 X 0.3 cm rows per well via bright field microscopy at 32X.

Protein Collection and Western Blotting of human NPCs

P3-P8 NPCs were plated in 35 mm dishes at a density of $1X10^6$ cells/dish and incubated. At 48h, cells were washed twice with ice-cold PBS followed by addition of M-PER (ThermoFisher, 78501) lysis buffer containing 1x HALT protease inhibitor consisting of AEBSF, aprotinin, bestatin, E-64, leupeptin and pepstatin A. Lysed samples were sonicated on ice using an Ultrasonics Sonicator (Qsonica, LLC, Newtown CT) and subsequently spun down at 4° C to pellet cell debris. The supernatant was then transferred and measured for protein levels. Protein concentration was measured with the BCA-protein assay (Pierce, Rockford, IL) in a spectrophotometer (Beckman, Indianapolis, IN), and calculated with comparison to a bovine serum albumin (BSA) standard curve. Equivalent protein extracts per lane were then separated on 12% acrylamide gel and transferred to polyvinylidenediflouride (PVDF; Millipore, cat # IPVH00010) membrane using transfer apparatus. The membranes were blocked with 5% milk and incubated with primary antibody overnight at 4 ^{o} C and followed by anti-mouse or anti- rabbit horseradish peroxidase (HRP)-conjugated secondary for 1h at room temperature or overnight at 4 ^{o} C. Bands were revealed by addition of a chemiluminescent reagent Western lightning™ Plus-ECL (Perkin Elmer, Waltham, MA) and system (Omega) as previously described (Mairet-Coello et al., 2009). Quantification of signal was conducted with ImageJ.

Primary antibodies utilized for these studies included phospho-p44/42 map kinase (Thr202/Tyr204) (1:1000, Cell signaling, Beverly, MA; cat# 9101S), Total p44/42 map kinase (1:1000, Cell signaling, Beverly, MA; cat# 9102S), and GAPDH (1:25000, Meridian Life Science, Memphis, TN).

Supplemental References

BARTLETT, C. W., FLAX, J. F., FERMANO, Z., HARE, A., HOU, L., PETRILL, S. A., BUYSKE, S. & BRZUSTOWICZ, L. M. 2012. Gene x gene interaction in shared etiology of autism and specific language impairment. *Biol Psychiatry,* 72**,** 692-9.

BARTLETT, C. W., HOU, L., FLAX, J. F., HARE, A., CHEONG, S. Y., FERMANO, Z., ZIMMERMAN-BIER, B., CARTWRIGHT, C., AZARO, M. A., BUYSKE, S. & BRZUSTOWICZ, L. M. 2014. A genome scan for loci shared by autism spectrum disorder and language impairment. *Am J Psychiatry,* 171**,** 72-81.

LU, N. & DICICCO-BLOOM, E. 1997. Pituitary adenylate cyclase-activating polypeptide is an autocrine inhibitor of mitosis in cultured cortical precursor cells. *Proc Natl Acad Sci U S A,* 94**,** 3357-62.

MAIRET-COELLO, G., TURY, A. & DICICCO-BLOOM, E. 2009. Insulin-like growth factor-1 promotes G(1)/S cell cycle progression through bidirectional regulation of cyclins and cyclin-dependent kinase inhibitors via the phosphatidylinositol 3-kinase/Akt pathway in developing rat cerebral cortex. *J Neurosci,* 29**,** 775-88.

SEKI, T., YUASA, S. & FUKUDA, K. 2012. Generation of induced pluripotent stem cells from a small amount of human peripheral blood using a combination of activated T cells and Sendai virus. *Nat Protoc,* 7**,** 718-28.

SIMONS VIP, C. 2012. Simons Variation in Individuals Project (Simons VIP): a genetics-first approach to studying autism spectrum and related neurodevelopmental disorders. *Neuron,* 73**,** 1063-7.

WILLIAMS, M., PREM, S., ZHOU, X., MATTESON, P., YEUNG, P. L., LU, C.-W., PANG, Z., BRZUSTOWICZ, L., MILLONIG, J. H. & DICICCO-BLOOM, E. 2018. Rapid Detection of Neurodevelopmental Phenotypes in Human Neural Precursor Cells (NPCs). *JoVE,* 133**,** e56628.
